# Gut microbial ecology and exposome of a healthy Pakistani cohort

**DOI:** 10.1186/s13099-024-00596-x

**Published:** 2024-01-22

**Authors:** Farzana Gul, Hilde Herrema, Mark Davids, Ciara Keating, Arshan Nasir, Umer Zeeshan Ijaz, Sundus Javed

**Affiliations:** 1 Department of Biosciences, COMSATS University Islamabad, Islamabad, 45550 Pakistan; 2 Department of Experimental Vascular Medicine, Amsterdam University Medical Centers, Location AMC, Amsterdam, The Netherlands; 3 School of Biodiversity, One Health & Veterinary Medicine, Graham Kerr Building, University of Glasgow, Glasgow, G12 8QQ UK; 4 Water & Environment Research Group, Mazumdar-Shaw Advanced Research Centre, University of Glasgow, Glasgow, G11 6EW UK; 5 Department of Molecular and Clinical Cancer Medicine, University of Liverpool, Liverpool, L69 7BE UK; 6 National University of Ireland, Galway, University Road, Galway, H91 TK33 Ireland; 7 Present Address: Moderna, Inc., Cambridge, MA USA

## Abstract

**Background:**

Pakistan is a multi-ethnic society where there is a disparity between dietary habits, genetic composition, and environmental exposures. The microbial ecology of healthy Pakistani gut in the context of anthropometric, sociodemographic, and dietary patterns holds interest by virtue of it being one of the most populous countries, and also being a Lower Middle Income Country (LMIC).

**Methods:**

16S rRNA profiling of healthy gut microbiome of normo-weight healthy Pakistani individuals from different regions of residence is performed with additional meta-data collected through filled questionnaires. The current health status is then linked to dietary patterns through ${\chi }^{2}$ test of independence and Generalized Linear Latent Variable Model (GLLVM) where distribution of individual microbes is regressed against all recorded sources of variability. To identify the core microbiome signature, a dynamic approach is used that considers into account species occupancy as well as consistency across assumed grouping of samples including organization by gender and province of residence. Fitting neutral modeling then revealed core microbiome that is selected by the environment.

**Results:**

A strong determinant of disparity is by province of residence. It is also established that the male microbiome is better adapted to the local niche than the female microbiome, and that there is microbial taxonomic and functional diversity in different ethnicities, dietary patterns and lifestyle habits. Some microbial genera, such as, *Megamonas, Porphyromonas, Haemophilus, Klebsiella* and *Finegoldia* showed significant associations with consumption of pickle, fresh fruits, rice, and cheese. Our analyses suggest current health status being associated with the diet, sleeping patterns, employment status, and the medical history.

**Conclusions:**

This study provides a snapshot of the healthy core Pakistani gut microbiome by focusing on the most populous provinces and ethnic groups residing in predominantly urban areas. The study serves a reference dataset for exploring variations in disease status and designing personalized dietary and lifestyle interventions to promote gut health, particularly in LMICs settings.

**Supplementary Information:**

The online version contains supplementary material available at 10.1186/s13099-024-00596-x.

## Background

The gut microbiota harbors the largest microbial community assemblage in humans and is considered vital due to its role in homeostatic regulation of several physiological processes, including metabolism, Short-chain fatty acid (SCFA) production, vitamin synthesis, digestion of certain dietary components and host immunity through prevention of pathogen colonization [1, 2]. Certain factors may induce temporary or permanent alterations in resident gut microbiota leading to gut dysbiosis. These include changes in diet, body mass index, exercise, antibiotic intake, stress and other psychological and environmental factors [3]. Gut dysbiosis is associated with diseases such as inflammatory bowel disease (IBD), *Clostridium difficile* infection [4], rheumatoid arthritis [5] mental health issues (stress, anxiety and depression) [6], autoimmune and allergic disorders as well as certain metabolic diseases like diabetes and obesity [4]. Simple therapeutic interventions for the treatment of some of these diseases through gut microbiome modulation have shown efficacy in a few studies [7, 8]. However, the transnational application of many gut modulation interventions is limited by the sheer diversity of an individual’s gut microbiome, as microbial composition and diversity vary even amongst healthy individuals [9] and is influenced by factors such as genetics, age, sex, and geographical location [3]. It is also known that the composition of gut microbiota remains relatively stable throughout the adult life [10]. Thus, ‘core’ healthy microbiomes may be relatively stable. Therefore, before gut modulation strategies can be widely adopted, we must first obtain a baseline understanding of the diversity of healthy gut microbiomes and define a core microbial community. This may also aid in predicting treatment efficacy through gut microbiome modulation which underscores the importance of microbiome research among healthy populations from diverse ethnicities and geographies.

Projects such as The Human Microbiome Project (HMP) have identified multiple healthy ‘core’ microbiomes [11]. However, the majority of gut microbiome projects focus on Western (American and European) populations [12–14], and some of the most populous countries (Pakistan, India and Bangladesh) in the world remain underrepresented [15]. As geographic location, ethnicity and sociocultural habits also influence gut microbial composition [16], the current global understanding of healthy microbial communities may not be applicable to much of the world’s population.

Pakistan is the fifth most populous country in the world with > 240 million population and multi-ethnic region, culturally diverse, with cultural and dietary influences from neighboring countries like Afghanistan and Iran predominating in the north and south western regions of the country and Indian influences in the eastern regions. Moreover, Pakistan shares genetic characteristics with the Indian population due to historic large-scale population immigration from India to Pakistan during the Indian sub-continent partition in 1947 [17]. Pakistan is one of the underrepresented countries for microbiome research in South Asia [18]*.* Few pilot studies have attempted to publish Pakistani gut microbiome, primarily focusing on the microbial signature in diseases and that too in a specific region missing in-depth analysis of healthy gut microbiome along with exposome [19–21]. Therefore, a comprehensive study discussing geography, ethnicity, dietary patterns and other lifestyle specific variations in microbiome composition and diversity of healthy adult Pakistani population is required.

To bridge this knowledge gap, fecal samples from healthy adult volunteers, aged between 18 and 40 years, belonging to different ethnicities, representing six major geographical regions in Pakistan were characterized based on high-throughput sequencing of 16S rRNA genes. We have applied traditional diversity metrics within the PERMANOVA framework to see if the changes in composition, phylogeny, and function of the microbial communities can be explained by the sources of variation. Using species abundance-occupancy diagrams and coupling them with neutral modelling, we have identified the core microbiome dynamically, considering province- and gender- specific occupancy of observed species. With neutral modelling, we are then able to identify which species are deterministically selected. Using ${\chi }^{2}$ test of independence and *Generalised Linear Latent Variable Model* (GLLVM), we have explored not only the dependencies between the observed categorical variables comprising diet, lifestyle and psychosocial factors, but have also linked them to the individual microbes. Some of the diversity analyses are then repeated for metabolic functions predicted for the microbial communities observed for each sample. These analyses then provide useful insights on the ethnic, ecological, psychosocial, and dietary drivers of gut microbiome composition and diversity in a healthy Pakistani populace.

## Results

### Pakistani core fecal microbiome shows distinctive phyla occupancy based on region and sex

**Fig. 1:**
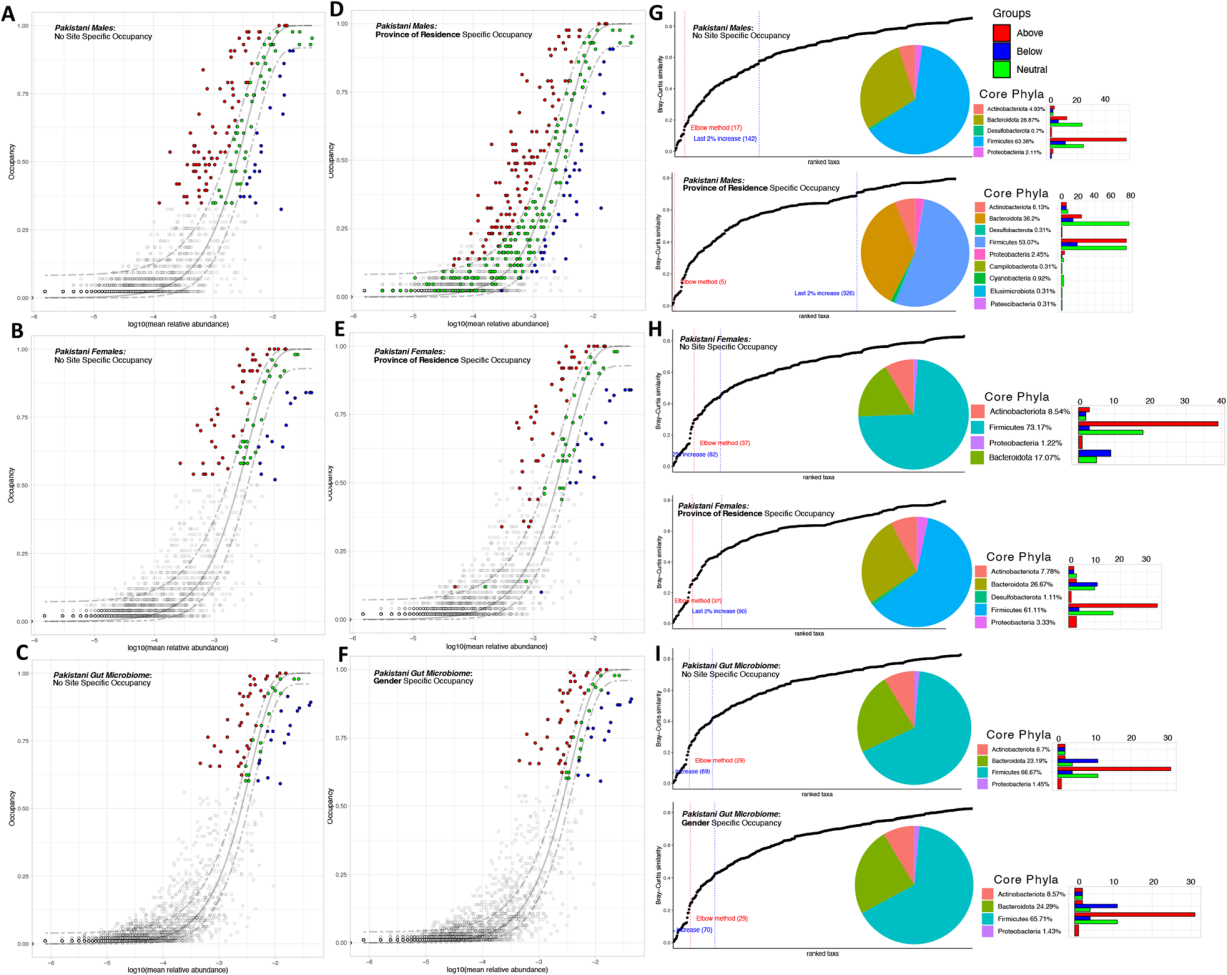
Core microbiome identified through species occupancy abundance diagrams. A stringent occupancy criteria **A**, **B**, **C** is incorporated where we clump all the samples in a single site (*no site specific occupancy)*, and then calculate the ranking of ASVs based on their occupancy and replicate consistency within a single category. Alternatively, we calculate the occupancy and replicate consistency of these ASVs separately (*site specific occupancy*) for each site where for **D**, site represents province of residence for males; for **E**, site represents province of residence for female; and for **F**, site represents the gender. Once we have obtained the rankings depending on which criteria used, Bray–Curtis similarity is calculated for the whole dataset, and then also for only the top-ranked taxa. The contribution of the top-ranked taxa is divided by the total Bray–Curtis similarity to calculate a percent contribution of the prospective core set to beta diversity. The next-ranked taxon is added consecutively to find the point in the ranking at which adding one more taxon offers diminishing returns on explanatory value for beta diversity (**G**, **H**, **I**). The red line represents the stringent “Elbow approach” where the change is maximal between the left and right side of dotted red threshold in terms of first-order differences, and “Last 2% decrease” criteria where ASVs are incorporated in the core subset until there is no more than 2% decrease in beta diversity. In this study, we are only identifying core microbiome (red, green and blue points) using “Last 2% decrease” criteria. Independently, a neutral model is fitted with those ASVs that fall within the 95% confidence interval (shown in green), and those that fall outside the 95% model confidence to be inferred as deterministically assembled, i.e., non-neutral ASVs. Points above the model are selected by the (host) environment (shown in red), and points below the model are dispersal limited (shown in blue). The proportion of core ASVs belonging to different phyla are then shown with a pie chart whilst the count of neutral/non-neutral ASVs are shown with the bar plots

First, core microbiome of Pakistani adults was established by considering different occupancy models, i.e., how we rank the amplicon sequencing variants (ASVs) to be part of the core microbiome. To do so, we consider gender and province of residence specific occupancies and within these groups replicate consistencies to suggest the ranking of ASVs (Fig. 1). By utilizing this ranking, we iteratively construct a core microbiome set stopping when there is diminishing return on explanatory power of beta diversity (Bray–Curtis contribution), i.e., doesn’t increase more than 2%. This dynamic approach is preferred over traditional approach where core microbiome is often based on a crisp threshold of 95% prevalence [22]*.*

We observed gender-based differences in the proportion of ASVs occupying the core microbiome. Without taking any site-specific occupancy (whether province of residence or gender), the minimum occupancy for ASVs being part of the core microbiome was ~ 33% for males (Fig. 1A) and ~ 52% for females (Fig. 1B), and ~ 59% when these are collated as a single Pakistani group (Fig. 1C). When we calculated occupancy separately for province of residency, the minimum occupancy threshold for males dropped down significantly to ~ 2% (Fig. 1D) suggesting that there is a local male microbial niche, where some ASVs are seen only in certain provinces, as shown in Additional file 1: Fig S1. The same was not observed for females (Fig. 1E), where the drop was not very significant (~ 10%). These results suggest male microbiome to be more well adapted to local niche than the female microbiome. When coupling with neutral modeling where the core microbiome was further discretized into three groups [Red (Above), selected by the host environment; Green, neutral; and Blue (Below), driven by dispersal limitation], for males, and no site-specific occupancy, the core microbiome belonged to five major phyla *Actinobacteriota, Bacteroidota, Desulfobacterota, Firmicutes* and *Proteobacteria* (Fig. 1G). Points above the neutral prediction were dominated by *Firmicutes* and *Bacteroidota*. Using province of residence specific occupancies, additional core phyla such as *Campylobacteriota, Cyanobacteria, Elusimicrobiota* and *Patescibacteria* appeared (Fig. 1G). In females with no site-specific occupancy, core phyla were similar to that of males, i.e., composed of *Actinobacteriota, Bacteroidota, Firmicutes* and *Proteobacteria* with absence of *Desulfobacterota.* Sex-based variation was observed between males and females with increased *Firmicutes* abundance in females and lower *Bacteroidota* abundance, as compared to males (Fig. 1H). Meanwhile*, Desulfobacterota* appeared as part of core phyla when province of residence-specific occupancy was used for females. This was mainly dominated by *Desulfovibrio* (ASV_160), which appeared mostly in residents of Islamabad Capital Territory (ICT), whether males or females (Additional file 1: Figs. S1 and S2). Interestingly, when we put all the male and female samples together, the core microbiome remained similar irrespective of whether a gender-specific occupancy model was used or not. In these cases, the core Pakistani phyla were dominated by *Firmicutes,* followed by *Bacteroidota*, *Actinobacteriota,* and *Proteobacteria* (Fig. 1I).

### Microbial taxonomic and functional diversity observed in different ethnicities, dietary patterns and lifestyle habits

In terms of composition, particularly alpha diversity estimates using richness and Shannon entropy, statistically significant differences were observed with various geographical, ethnic, sociodemographic and dietary covariates. Lower microbial richness and Shannon diversity was observed in participants reporting province of birth and residence as Baluchistan and Khyber Pakhtunkhwa (KPK) compared to Sindh, Punjab, ICT and Azad Jammu & Kashmir (AJK) (Additional file 1: Fig. S5). In ethnic groups, Saraiki (n = 4) showed higher diversity as compared to other ethnicities [Punjabi (n = 37), Urdu speaking (n = 6), Kashmiri (n = 9), Pathan (n = 22), Sindhi (n = 6) and Balochi (n = 7)] (Additional file 1: Fig. S5). With regards to diet, participants eating pickle and soft cheese regularly showed significantly lower microbial diversity (Additional file 1: Fig. S4). Interestingly, certain sociodemographic and lifestyle variables also depicted shift in alpha diversity. People who were self-employed had decreased microbial diversity as compared to students and full-time employees (Additional file 1: Fig. S5). A gradual decrease in diversity was also observed with different levels of tiredness reported by the participants (Additional file 1: Fig. S7).

In terms of functional diversities, analyzed using recovered KEGG orthologs and MetaCyc pathways abundances, participants reporting province of residence as KPK showed higher functional diversity for richness and Shannon entropy (Additional file 1: Fig. S10). In participants with province of birth and residence as Punjab and KPK, higher MetaCyc pathways diversity was observed respectively (Additional file 1: Fig. S14). A gradual increase in functional diversity was observed from low to high socioeconomic status (Additional file 1: Fig. S10). On the other hand, a gradual decrease in KEGG orthologs and MetaCyc pathways diversity was observed with different levels of tiredness (Additional file 1: Figs. S11 and S14). Participants reporting regular throat issues also exhibited a decrease in MetaCyc pathway diversity (Additional file 1: Fig. S16).

In terms of beta diversity, marked variation was observed in gender (Additional file 1: Fig. S17) ethnicity, province of birth and residence (Additional file 1: Fig. S20), feelings of wellbeing, tiredness (Additional file 1: Fig. S17), parasitic infection treatment (Additional file 1: Fig. S19), antibiotic intake in childhood, trouble falling asleep and regular throat issues (Additional file 1: Fig. S21). Variation was also observed in some dietary items consumption such as fresh fruits, bread, soft cheese, pickle and honey (Additional file 1: Figs. S18 and S19).

### Current health status of Pakistani adults is associated with diet, employment status, sleeping pattern and medical history

We first analyzed various dependencies between characteristics of study participants, collected through a self-recorded questionnaire provided at the time of sample collection. This was done using ${\chi }^{2}$ test of independence and then the relationships were explored further using Pearson residual to identify what are the attractors and repellents within the observed categories of two variables where the ${\chi }^{2}$ test came out to be significant. The primarily variable was self-reported current health status, against which the relationships were sought. The results are shown in Additional file 1: Figs. S22–S47, and then summarised in Tables 1, 2, and 3 in terms of most significant attractors. Our results showed that poor health status was positively associated with acid reflux and anaemia (Additional file 1: Fig. S22), bad breath (Additional file 1: Fig. S23), gut flare-ups (Additional file 1: Fig. S32), lactose sensitivity, lethargy (Additional file 1: Fig. S33), anxiety (Additional file 1: Fig. S39), stress (Additional file 1: Fig. S40), throat infections (Additional file 1: Fig. S41), disturbed sleeping patterns (Additional file 1: Figs. S42, S44 and S45), and part-time employment status (Additional file 1: Fig. S30). Amongst dietary variables, natural spring water as a drinking water source (Additional file 1: Fig. S28), consumption of beef (Additional file 1: Fig. S24), coffee (Additional file 1: Fig. S26), dry fruits (Additional file 1: Fig. S29), honey (Additional file 1: Fig. S34), and oatmeal correlated with poor health status (Additional file 1: Fig. S36). Good and moderate health status is positively associated with mutton consumption (Additional file 1: Fig. S35) and taking medication to treat constipation (Additional file 1: Fig. S27) respectively. Meanwhile, excellent current health status was positively associated with parasitic infection during childhood (Additional file 1: Fig. S25) or its treatment (Additional file 1: Fig. S36), male sex (Additional file 1: Fig. S29), defecation frequency of twice a day (Additional file 1: Fig. S31), regular exercise (Additional file 1: Fig. S41), and vaccination with hepatitis and polio vaccines (Additional file 1: Figs. S43 and S46).

**Table 1:**
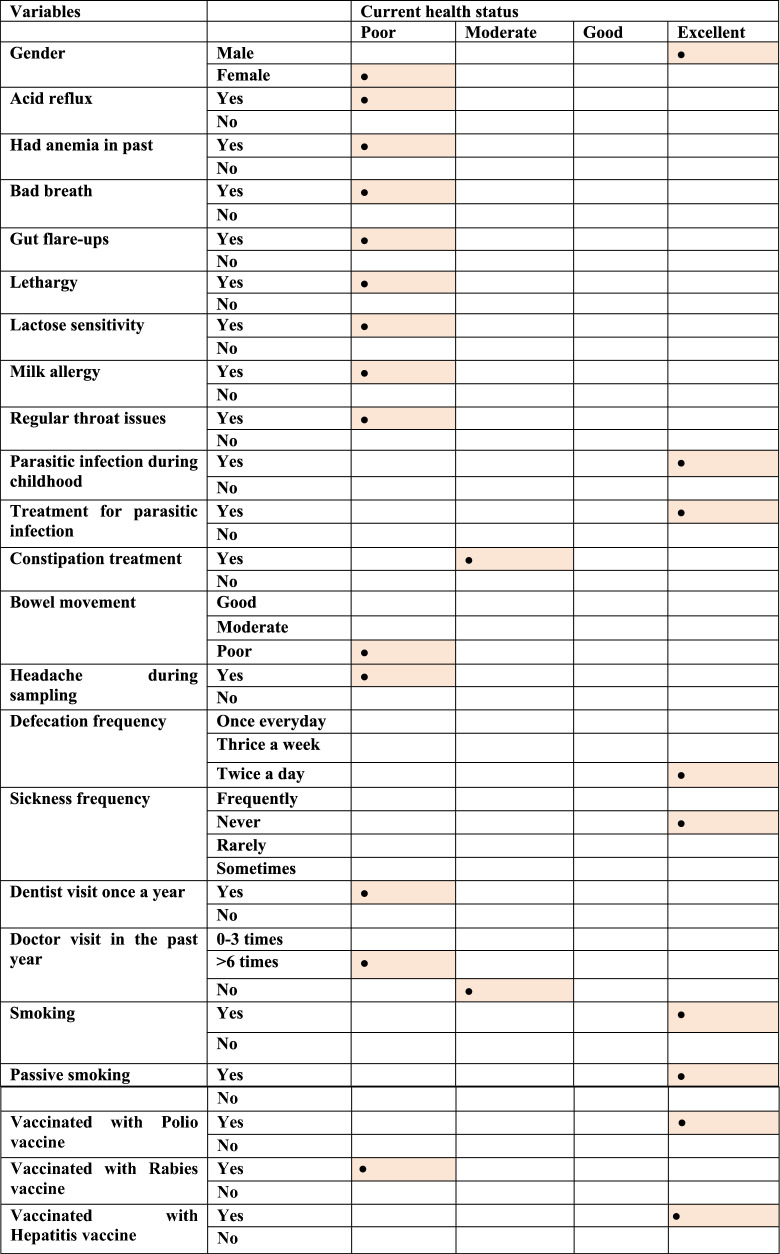
Strongest positive attractors of current health status in respondents categorized under clinical history

The results are based on ${\chi }^{2}$ test of independence using Pearson residuals summarizing results shown in Additional file 1: Figs. S22–S47. The highlighted cells show the strongest relationship recovered between the factors of two variables

**Table 2:**
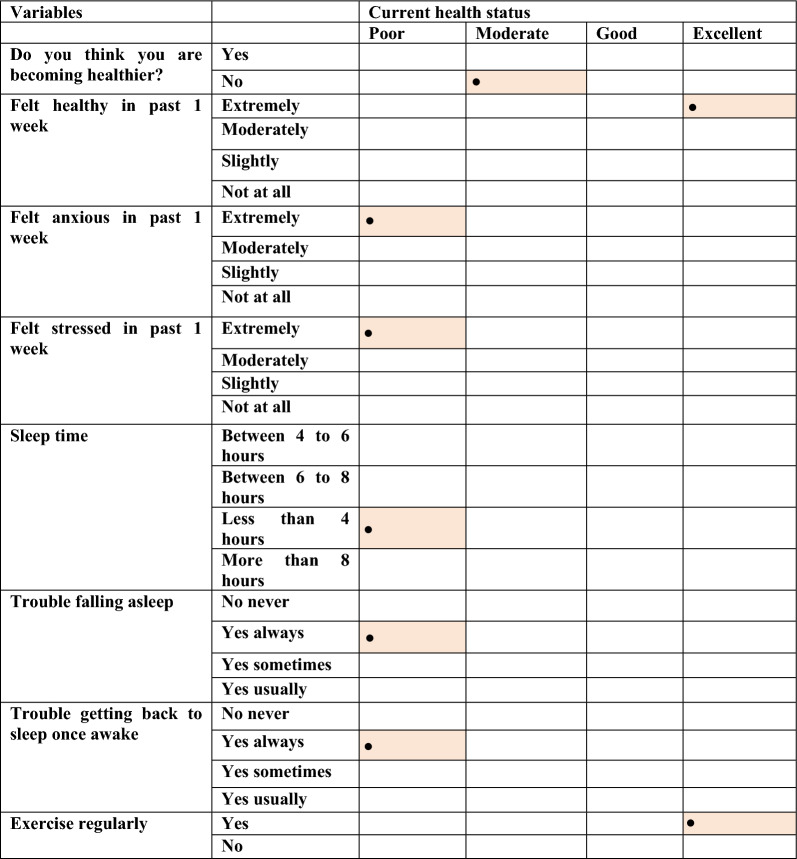
Strongest positive attractors of current health status in respondents categorized under psychological and behavioural patterns

The results are based on ${\chi }^{2}$ test of independence using Pearson residuals summarizing results shown in Additional file 1: Figs. S22–S47. The highlighted cells show the strongest relationship recovered between the factors of two variables

**Table 3:**
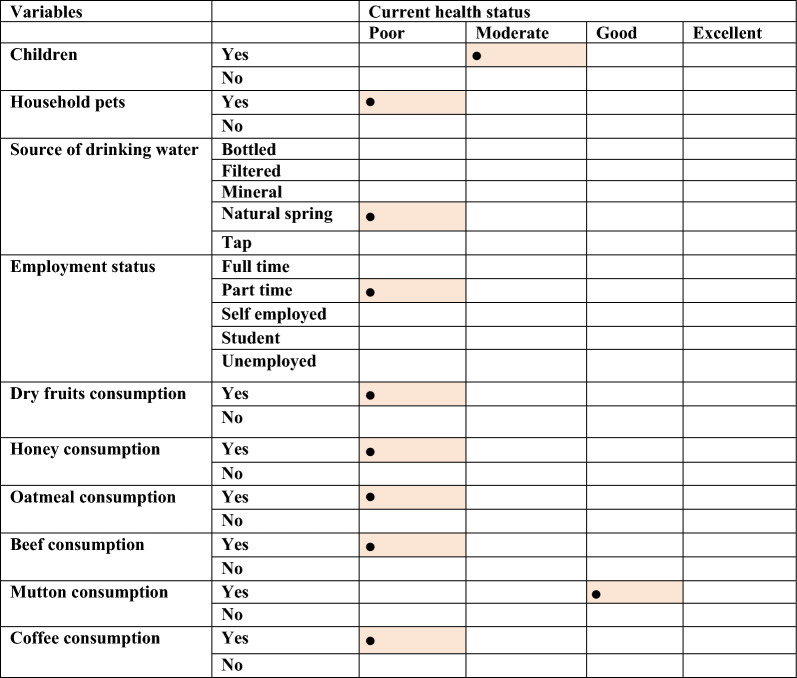
Strongest positive attractors of current health status in respondents categorized under socioeconomic and dietary factors

The results are based on ${\chi }^{2}$ test of independence using Pearson residuals summarizing results shown in Additional file 1: Figs. S22–S47. The highlighted cells show the strongest relationship recovered between the factors of two variables

### Sociodemographic, anthropometric and dietary factors are the key drivers of variation in microbial composition and functions

We next performed PERMANOVA analysis to assess variability in microbiome using different beta diversity indices (i.e., Bray–Curtis, Unweighted UniFrac, weighted UniFrac and Hierarchical Meta-Storms) to ascertain how microbiota, phylogeny, and function changes with dietary habits and lifestyle (Additional file 1: Table S1). Using R^2^ in PERMANOVA, if significant (p < 0.05), represents the variability explained by that variable. The variables which showed strong association for at least three of the beta diversity distance measures have been highlighted in Additional file 1: Table S1. For example, we found that respondents who were given antibiotics during their childhood have shown significant variability in terms of microbial composition (3.3% variability) and phylogeny (7.4% variability). Other significant factors which accounted for variability in microbial composition, phylogeny and function include how people generally felt about their health (1.6% variability in composition) and whether they were recently tired (5.3% variability in phylogeny). The BMI and gender accounted for 1.7% variability in composition, and 1.7% variability in function, respectively. Similarly, consumption of honey (1.6% variability in composition; 3.4% variability in phylogeny), pickle (2.8% variability in composition; 3.3% variability in phylogeny), rice (2.5% variability in phylogeny; 2% variability in function) and soft cheese (2.8% variability in composition; 3.3% variability in phylogeny) were all implicated as significant covariates.

### Key genera implicated with sources of variability

**Fig. 2:**
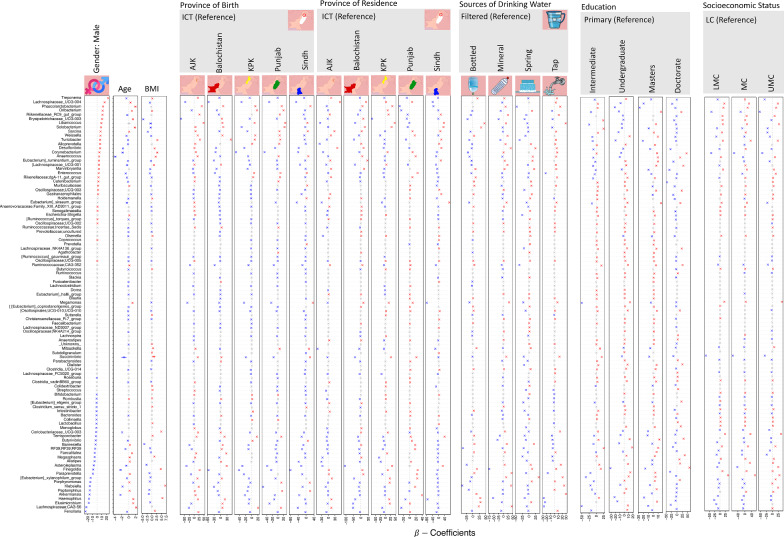
${\varvec{\beta}}$ coefficients returned from GLLVM procedure for covariates considered in this study by considering top 100 most abundant genera incorporating both continuous as well as categorical labelling of samples. Those coefficients which are positively associated with the microbial abundance of a particular species are represented in red colour whilst those that are negatively associated are represented with blue colour, respectively. Where the coefficients are non-significant, i.e., the 95% confidence interval crosses the 0 boundary, they are greyed out. Since the collation of ASVs was performed at Genus level, all those ASVs that cannot be categorized based on taxonomy are collated under “__Unknowns__” category. The acronyms are as follows: (*ICT* Islamabad Capital Territory, *AJK* Azad Jammu & Kashmir, *KPK* Khyber Pakhtunkhwa, *LC* Lower Class, *LMC* Lower Middle Class, *MC* Middle Class, *UMC* Upper Middle Class). Note that the GLLVM procedure additionally calculates the residual covariance matrix of the latent variables in the model which gives an additional co-occurrence relationship between microbes, and is given in Additional file 1: Fig. S47

**Fig. 3:**
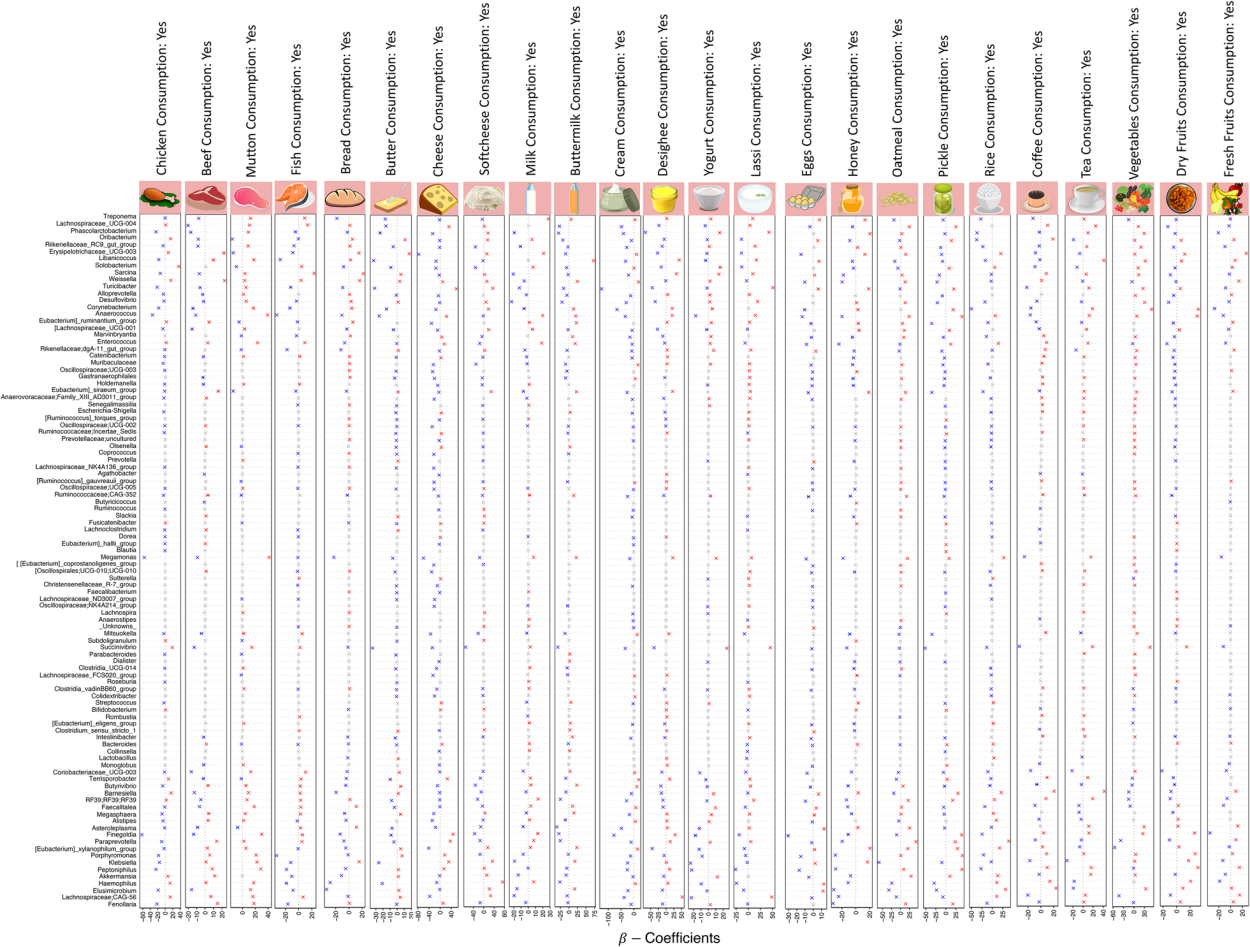
${\varvec{\beta}}$ coefficients for covariates categorized under dietary items

We then analyzed the top 100 most abundant microbial taxa against all sources of variation by fitting a generalized linear latent variable model (GLLVM) to find the covariates that on average caused a substantial change in the abundance of the microbial taxa. These covariates included gender, age, BMI, province of birth and residence, education, source of drinking water, socioeconomic status, different food items consumption and dietary habits with results shown in Figs. 2, 3, and, 4, respectively, and summarized in Additional file 1: Table S2 in terms of top 5 most significantly positive and negatively associated genera for a given covariate (51 genera in total). We then only considered genera associated with the covariates that showed significant changes in alpha or beta diversity (22 genera in total). These covariates are highlighted in Additional file 1: Table S2 with a grey background. Some of the genera which were positively or negatively associated with gender, provinces of birth and residence in comparison to ICT were SCFA producers such as *Lachnospiraceae; CAG-56, Phascolarctobacterium, Turicibacter, Lachnospiraceae_UCG-004, [Eubacterium]_ruminantium_group, Peptoniphilus, [Eubacterium]_siraeum_group, [Eubacterium]_xylanophilum_group*, *Mitsuokella* and *Paraprevotella.* These genera were also positively associated with fresh fruits, soft cheese and honey consumption and negatively associated with pickle consumption. Other genera which were negatively associated with Punjab, Balochistan and KPK in comparison to ICT were mostly non-SCFA producer microbes such as *Solobacterium, Haemophilus, Klebsiella, Elusimicrobium* and *Corynebacterium*. *Haemophilus*, *Klebsiella* and *Succinivibrio* were positively associated with soft cheese consumption and negatively associated with pickle and fresh fruits consumption. *Finegoldia, Asteroleplasma, Erysipelotrichaceae_UCG-003, Elusimicrobium, Megamonas* and *Porphyromonas* were positively associated with pickle, rice and fresh fruits consumption (Additional file 1: Table S2).

**Fig. 4:**
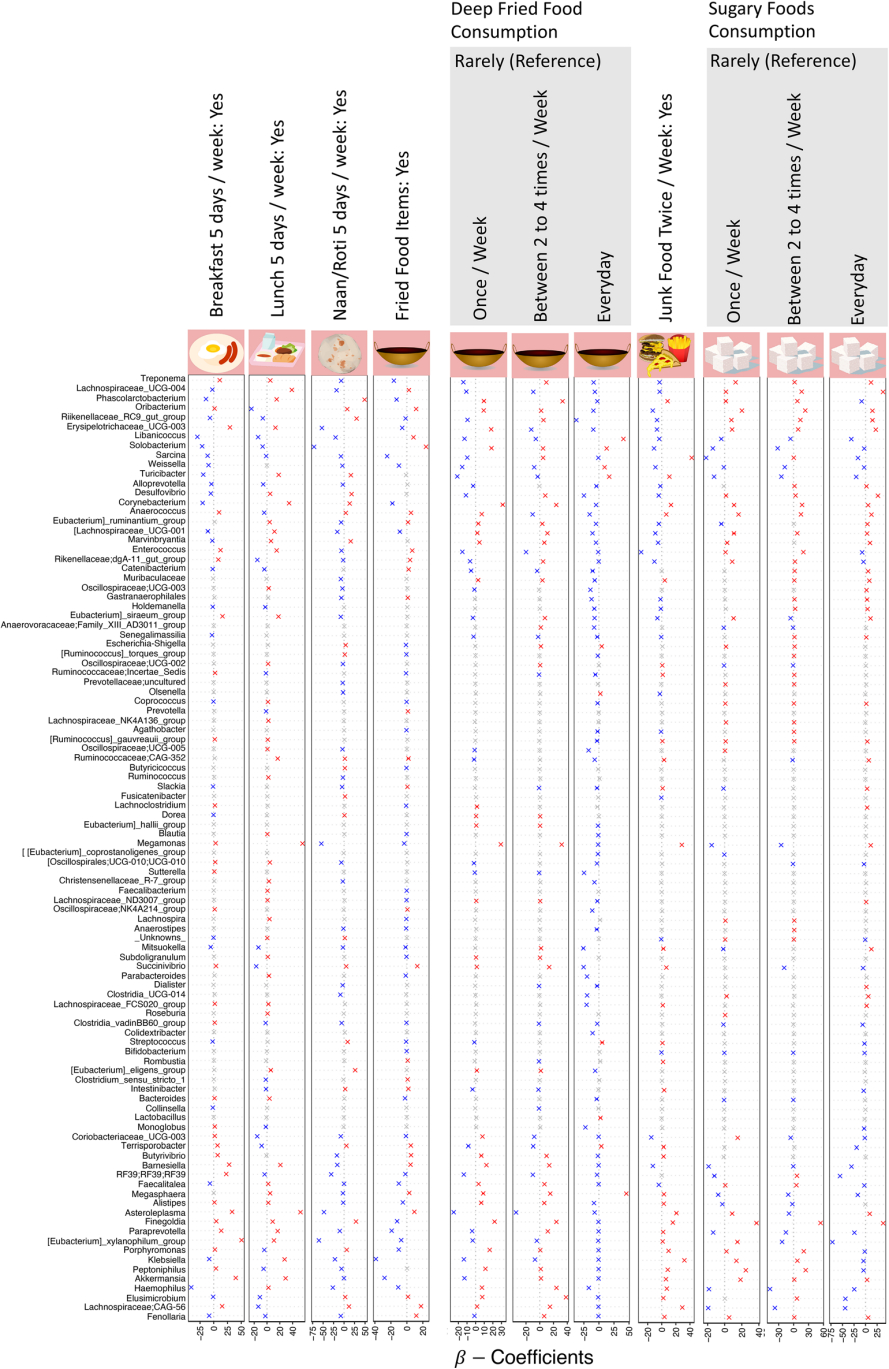
${\varvec{\beta}}$ coefficients for covariates categorized under intake frequency of selected dietary items

## Discussion

Our study provides an extensive examination of the core microbiome of Pakistani population with consideration to the exposome driving the gut microbiomes. Using a dynamic approach based on occupancy models for identifying the core microbiome, our results demonstrated that the gut microbiome of males was more diverse than the gut microbiome of females. Moreover, we observed that the male and female core microbiomes were influenced by place of residence. It has been observed that a larger number of males migrate from their place of birth to urban areas to find better opportunities for education and earning as compared to females [23]. Apart from four major phyla such as *Firmicutes, Bacteroidota, Actinobacteriota* and *Proteobacteria,* we observed *Desulfobacterota* to be part of core microbiome of male and females when grouped on the basis of province of residence. People living in ICT (Islamabad Capital Territory) showed higher abundance of *Desulfobacterota* in comparison to other provinces of residence. *Desulfobacterota* are sulfate-reducing bacteria, associated with gut inflammation and increased immune response [24]. Moreover, lower abundance of *Firmicutes* and higher abundance of *Bacteriodota* was observed in males as compared to females. High or low *Firmicutes* to *Bacteriodetes* ratio is associated with obesity and inflammatory bowel disease respectively [25]. Therefore, it is not surprising that greater incidence of IBD and obesity is observed amongst females [26, 27]. It must be noted that higher *Firmicutes* to *Bacteroidetes* ratio is also consistently reported amongst female individuals in various studies [28, 29] and therefore present a normal physiological response and any association to disease must be determined in context of other risk factors.

We also observed *Campilobacterota, Cyanobacteria, Elusimicrobiota,* and *Patescibacteria* as core phyla in Pakistani males with respect to Province of residence. *Campilobacterota* are associated with development of gastrointestinal diseases such as inflammatory bowel disease (IBD) and Ulcerative colitis [30]. *Cyanobacteria,* and*, Elusimicrobiota,* are also found in groundwater, as well as animal gut and soil, and the latter are associated with nitrogen metabolism [31, 32]*. Patescibacteria* were also detected as part of core microbiome in males with site specific occupancy, which have small genome size are parasitic in nature and found in groundwater in rural regions [33], therefore their prevalence may indicate exposure to rural environments. *Campilobacterota, Elusimicrobiota,* and *Patescibacteria* have been previously reported as phyla prevalent in healthy individuals from west Bengal [34].

Next our focus was to determine how taxonomic and functional diversity of gut microbiome changes in different geographical location (province of birth and residence), ethnicities, dietary patterns and lifestyle habits. We used alpha and beta diversity measures and PERMANOVA to observe the variations in microbiome. Pakistan is a multi-ethnic society, with disparate dietary rituals and lifestyle. For alpha diversity, our results indicate that both birth province and the province of residence as well as different ethnicities explain changes in microbial and functional diversity. Balochistan (province of birth) and KPK (province of residence) showed lower microbial diversity and higher KEGG orthologs and MetaCyc pathway diversity as compared to other provinces demonstrating differences in dietary patterns and cultural habits. People living in Balochistan and KPK consume more meat based diet which is in line with previous observations that meat consumption reduces the richness and microbial diversity [35]. Balochi and Pathan ethnicities showed lower diversity as compared to Saraiki which mostly reside within Punjab province and have variety of food consumption based on meat, vegetables and fruits.

Intake of vegetables and fruits is already reported to be associated with increased microbial diversity [36]. In contrast to previously reported studies [37, 38], pickle and soft cheese consumption were observed to be associated with lower alpha diversity and significant variation in beta diversity in this study. Pickle is considered as a potential source of probiotics and has been reported to reduce the risk of long term diabetes [38, 39]. However, the pickle production process in Pakistan and India is very different from other countries. For instance, the pickled food items are usually fermented in spices before preserving them in oil, whereas common pickling processes in other regions rely on preserving blanched vegetables in vinegar [40]. Similarly, Cheese consumption also reduces the risk of metabolic syndrome and plasma cholesterol level but no evidence of cheese consumption effects on microbial alpha and beta diversity was observed previously [37].

Self-employment and low socioeconomic status were also found to be associated with low microbial and KEGG orthologs diversity and poor health status. Low socioeconomic status has also previously shown to be associated with reduced alpha diversity [41]. It is interesting to note that lower microbial, KEGG orthologs and MetaCyc pathways diversity was observed in respondents reporting extreme tiredness and poor health status. Chronic fatigue has been previously reported to reduce the microbial and functional diversity [42].

In terms of beta diversity, gender, parasitic infection treatment, antibiotic intake in childhood, trouble falling asleep, regular throat issues, bread and fresh fruits consumption were amongst the major factors observed to be causing changes. It is well known that gender plays an important role in shaping gut microbial structure [43]. Studies have reported differences in gut microbial diversity and composition of males and females [44, 45] that could be explained by differences in hormones which impact innate immune responses [43]. Parasitic infection is also known to be associated with alteration in gut microbial diversity that could be due to parasite-induced Th2 immune response which triggers the disturbance in gut microbiota composition and diversity [46]. Antibiotic intake in childhood is reported to induce the changes in gut microbial composition and diversity which persists even after years [47]. Poor sleeping habits which were observed to be associated with poor health status also reduce the microbial diversity and are reported to modify the gut microbial composition which in turn can promote insulin resistance and systemic inflammation [48]. Regular throat issues which were observed to be associated with change in beta diversity are in line with what is previously reported. Studies have shown that respiratory tract infections can reduce the microbial diversity, especially members of *Ruminococcaceae* and *Lachnospiraceae* family which were reduced in respondents reporting regular throat infections. These are SCFA producers and are known to play important role in gut barrier functions [49]. Fruits are an important part of healthy diet and known to increase the microbial composition and diversity. They maintain intestinal mucosal integrity and improve anti-inflammatory properties and insulin sensitivity through short-chain fatty acids (SCFA) production [50].

We then focused our attention on finding the association between the self-reported health status of Pakistani adults with some of the abovementioned variables associated with significant changes in gut microbial diversity. Host dietary and lifestyle patterns can substantially impact the gut microbiome, which in turn can influence status of wellbeing. We found that poor health status was mainly associated with medical history of acid reflux, anemia, gut flare-ups, bad breath, stress, anxiety and lactose sensitivity. Acid reflux is associated with poor health status and previously reported to reduce the microbial diversity [51]. Stress, anxiety and acid reflux are reported to be interlinked, as stress and anxiety are often among the factors associated with acid reflux [52]. Halitosis (bad breath) causing bacteria *Solobacterium* is observed to be present abundantly in our study and is associated with poor health. Bad breath can be due to poor oral hygiene, certain foods, smoking and medical conditions [53]. Drinking unsafe and contaminated water can also compromise the health status. Drinking water source was associated with poor health status and is already known to enrich certain antimicrobial resistance genes in gut microbial communities of Pakistanis [54]. Dry fruits also showed association with poor health which could be explained by the fruits drying procedure. Studies have reported that fruits dried in open air or unhygienic conditions may be contaminated with microorganisms which can cause life threatening health issues [55]. Honey consumption is considered as a natural source of vitamins and polyphenolic compounds that provide health beneficial effects. However, it is reported that honey collected from toxic plants can cause hazardous effects to health [56]. Defecation frequency twice a day and regular exercise were shown to be associated with excellent health status are in line with the previous studies [51, 57].

Finally, we used GLLVM to find the association of microbial taxa with key variables analysed in this study. Amongst the top highly abundant genera, microbial taxa with SCFA producing properties had positive/negative association with gender, province of birth and residence and some food items (soft cheese, fresh fruits, honey, rice and pickle) consumption. For example, *Lachnospiraceae; CAG-56* had a strong positive association with KPK (province of birth) compared to ICT, soft cheese and fresh fruits consumption, and negatively associated with gender (male), AJK and Sindh (province of birth), Punjab and Balochistan (province of residence) as well as honey consumption. *Lachnospiraceae_UCG-004* and *[Eubacterium]_xylanophilum_group* had positive association with gender and AJK, Punjab and balochistan (province of residence) and negative association with all provinces of birth and honey consumption. Whereas *[Eubacterium]_ruminantium_group* were negatively associated with pickle consumption. All these genera belong to family *Lachnospiraceae*, members of which are SCFA producers and known to inhibit intestinal inflammation, maintain the intestinal barrier, and modulate the gut motility [58]. *Lachnospiraceae;CAG* are associated with high fibre diet and complex carbohydrates [59]. Amongst *Lachnospiraceae, [Eubacterium]_xylanophilum_group* has been involved in lipid and glucose metabolism [60]. *Phscolarctobacterium* also showed positive association with gender and honey consumption and negative association with rice and fresh fruits consumption. These are also SCFA producers and studies have reported their higher abundance between age group of 18–40 years. They are also observed to be associated with high fat diet, starchy food and grain consumption [61, 62]. Other SCFA producers include *Peptoniphilus, Mitsuokella, Megamonas* and *Paraprevotella. Peptoniphilus* is an opportunistic pathogen which can cause bloodstream, diabetic skin and soft tissue infections [63]*.* Whereas *Megamonas, Mitsuokella* and *Paraprevotella* are previously reported as part of healthy gut microbiome in Indian population [64, 65]. Other non SCFA producers which showed association with the co-variates included *Solobacterium, Haemophilus, Klebsiella, Elusimicrobium*, *Corynebacterium*. *Finegoldia,* and *Erysipelotrichaceae_UCG-003. Solobacterium* which causes halitosis (foul smell or oral malodour) and oral infections were most abundantly present in people belonging to Balochistan, KPK and Sindh provinces [66]. *Haemophilus, Finegoldia,* and *Corynebacterium* were decreased with fresh fruits consumption. *Haemophilus* are the part of salivary microbiome and some of the species can cause respiratory infections [67, 68]. *Finegoldia* are previously associated with high BMI and sweets consumption [69]. *Corynebacterium* are gram-positive bacilli, including many toxigenic species which cause respiratory tract infections such as diphtheria [70]. A recent study has reported that fresh fruit consumption, especially mangoes can increase the abundance of *Corynebacterium pyruviciproducens* which is considered as immune modulator [71]. *Erysipelotrichaceae_UCG-003* increased with pickle, rice and fresh fruits consumption and is previously observed to be enriched with high fiber diet intake [72].

## Conclusions

This study provides an early snapshot of the healthy core Pakistani gut microbiome by focusing on the most populous provinces and ethnic groups residing in predominantly urban areas. Our interpretations are based on studying the gut microbial profiles of limited sample size of 117 healthy individuals relying on partial sequencing of the 16S rRNA gene. We believe that profiling of less populous ethnic groups and provinces within the country with greater representation from rural areas may provide additional insights into the diversity of healthy Pakistani gut microbiome. We compensate the limitations by using state-of-the-art analytical tools to provide an in-depth exploration of microbial communities in association with current health status, impact of sociodemographic factors and dietary patterns on microbial communities within the healthy gut, suggesting gut microbiome heterogeneity. The study may serve as a reference for exploring variations with disease status and may play a role in designing personalized dietary and lifestyle interventions to promote gut health. Moreover, knowledge about key microbial species in the healthy gut aids in the development of therapeutic strategies to modulate microbiome, such as prebiotics, probiotics, fecal microbiota transplant (FMT), and phage therapies.

## Materials and methods

### Study participants identification and recruitment

117 participants (61 females and 56 males) were initially identified and recruited for the study. All the participants were screened through a questionnaire based on inclusion/exclusion criteria as follows. Participants were aged between 18 and 40 (mean age 28.7 ± 5.45) and belonged to six major geographic regions [Punjab (n = 40), Sindh (n = 6), Balochistan (n = 10), KPK (Khyber Pakhtunkhwa) (n = 15), ICT (Islamabad Capital Territory) (n = 14) and AJK (Azad Jammu & Kashmir) (n = 8)] and major ethnic typification [Punjabi (n = 37), Pathan (n = 22), Kashmiri (n = 9), Balochi (n = 7), Saraiki (n = 4), Sindhi (n = 6) and Urdu speaking (n = 6)]. The major exclusion criteria were age < 18 or > 40, body mass index (BMI) either < 18 or > 30 kg/m^2^, antibiotic or multivitamin intake within the last three months, any prior clinical history of chronic or acute infections or other diseases, pregnant or lactating females, or females with irregular menstrual cycles (i.e., less than 21 or more than 35 days apart). All the participants were asked to mention their province of birth and residence because at the time of sampling, some participants were residing at their place of birth whereas others have relocated to other cities/Provinces. Majority of participants were from urban areas comprising of students or young professionals who migrated from their place of birth and living in ICT for last 2–3 years.

### Sample collection

All the participants were briefed on the stool sampling methodology and were given uBiome Explorer kits. These kits follow the protocols outlined by the NIH Human Microbiome Project [[73] Available online at: http://www.fda.gov/cder/guidance/959fnl.pdf (accessed 22 August 2023)]. These were then shipped to Microbiota Centre of Amsterdam (MiCA) in the Netherlands for subsequent downstream processing.

### DNA extraction and PCR amplification

DNA extraction and PCR amplification were performed in the Microbiota Centre of Amsterdam (MiCA), Amsterdam. First, sample collection tubes were centrifuged at 14,000 RPM/18,626 RCF (fixed angle) for 10 min at room temperature and stabilizing buffer was removed. Next, DNA from fecal samples was extracted using a repeated bead beating protocol and purified using the Maxwell RSC Whole Blood DNA kit [74]. Purified DNA concentration was measured by using the Qubit®dsDNA BR Assay with 96 well plate (Invitrogen—Carlsbad, California, United States). Four sample collection kits containing only solubilizing buffer with no stool samples were used as negative control and were followed for the same extraction steps. V3-V4 amplicon sequencing was selected based on its established utility as the most appropriate choice, with low error propagation in Illumina sequencers as described previously [75–77]. The V3–V4 region of the 16S ribosomal RNA (rRNA) gene was amplified using a single step PCR protocol using universal primers, B341 F and B806R. Ampure XP beads were then used to purify the amplicon libraries and purified products were pooled equimolarly [78]. The library was sequenced with an Illumina MiSeq platform using v3 chemistry with 2 × 250 cycles.

### Bioinformatics

Abundance tables were generated by constructing Amplicon Sequencing Variants (ASVs) using the QIIME2 workflow [79] and the DADA2 denoising algorithm [80], in which both forward and reverse reads are denoised and merged together (using qiime dada2 denoise-paired command)*.* Additionally, MAFFT [81] and FastTree [82] were used using qiime phylogeny align-to-tree-mafft-fasttree command to generate the rooted phylogenetic tree. Full details of the commands are provided at https://github.com/umerijaz/tutorials/blob/master/qiime2_tutorial.md and are similar to methods (bioinformatics) previously published by the authors [83]*.* The samples for this study form a subset of a larger gut associated study with all the samples pooled together to generate a single abundance table (n = 176 samples x P = 4751 ASVs). These samples included 4 negative controls, which were later used to identify and remove contaminants (11 ASVs) by employing the *Prevalence Method* in R’s decontam package [84]. Additionally, PICRUSt2 algorithm [85] as a QIIME2 plugin (using qiime picrust2 full-pipeline command) was used on the ASVs to predict the functional abundance of microbial communities (both KEGG enzymes and MetaCyc pathways were recovered) by using the weighted Nearest Sequenced Taxon Index (NSTI) threshold of 2.0 in the software to map the ASVs against the reference database comprising ~ 20,000 genomes (whose functions were known) in PICRUSt2. Only 4 ASVs out of 4,751 did not match, and thus a very high alignment (~ 99%) increases our confidence in the prediction quality. We then classified the ASVs using the recent SILVA SSU Ref NR database release v.138 [86] using qiime feature-classifier classify-sklearn command, and then combined the taxonomic information with the abundance table to generate a BIOM file. The rooted phylogenetic tree, also generated using the QIIME2 framework, along with the above BIOM file as well as the functional tables from PICRUSt2 were then used in the downstream statistical analyses in R. For visualization, the clip arts were either drawn by the authors or using the repository https://creazilla.com/ where stock images are available freely for personal or commercial projects without asking for permission from the original authors.

### Statistical methods

As a pre-processing step, we removed typical contaminants such as *Mitochondria*, and *Chloroplasts*, as well as any ASVs that were unassigned at all levels, as per recommendations given at https://docs.qiime2.org/2022.8/tutorials/filtering/. We further used R’s decontam package [84] to identify and remove contaminants (11 ASVs) from 4 negative control samples, by employing the *Prevalence Method*. Of 170 samples, only 117 samples were relevant to this study. After filtering out low yield samples (< 2000 reads), we were left with a final abundance table of n = 93 samples × P = 3437 ASVs, on which we performed the statistical analyses. The summary statistics of sample-wise read distributions are as follows: [Minimum: 13,622; 1st Quartile: 19,732; Median: 21,655; Mean: 22,356; 3rd Quartile: 24,993; Maximum: 36,990].

#### Diversity measures

R’s vegan package [87] was used for alpha and beta diversity analyses. For alpha diversity we used: (i) Shannon entropy; and (ii) rarefied richness.

Beta diversity was calculated using four different distance measures: (i) Bray–Curtis distance on the ASV abundance table to visualize the compositional changes; (ii) Unweighted UniFrac distance estimated using R’s Phyloseq package [88] to see changes between samples in terms of phylogeny; (iii) Weighted UniFrac, abundance weighted version of UniFrac; and (ii) Hierarchical Meta-Storms (HMS) [89]. Ordination of ASV table in reduced space (beta diversity) was done using Principal Coordinate Analysis (PCoA) using the R’ Vegan’s package, mainly using Bray–Curtis distance. Additionally, Vegan package was also used to perform PERMANOVA analyses to see if the microbial or functional community structures can be explained by different sources of variability.

#### Core microbiome

To identify core microbiome, we have used the approach discussed in [90].The approach first ranks the ASVs by occupancy (from highly prevalent to lowly prevalent) according to study design, and then calculates the minimal prevalence threshold dynamically by learning from the data. After ranking the ASVs, the subset of core taxa is constructed incremently by adding highly prevalent to lowly prevalent, and then quantifying the contribution of the core subsets to beta diversity using the Bray–Curtis distance in the equation, $C=1-\frac{B{C}_{core}}{B{C}_{all}}$. The authors have specified two approaches to decide at what threshold the core subset construction stops: (a) where addition of an ASV does not cause more than 2% increase in the explanatory value by Bray–Curtis distance; and (b), an “elbow” approach where first order differences are calculated by partitioning the curve in two parts, and calculating the difference in the average rates of change for both of these parts. A point at which this difference is maximized is the elbow point. Approach (b) is very stringent and therefore approach (a) was used as recommended by the original authors. Independently, a neutral model [91] is fitted to the “S” shaped abundance-occupancy distributions inform the ASVs that are likely selected by the environment. These are obtained as those that fall outside the 95% confidence interval of the fitted model, and are inferred to be deterministically assembled, rather than neutrally selected, with those that are above the model selected by the host environment (represented by red colour), and those points below the model are dispersal limited (represented by blue colour).

To incorporate heterogeneity caused by spatial/cross-sectional consideration (province of residence, gender, etc. of all the subjects who gave their gut samples), we have used two approaches as per original author’s suggestion: a) a conservative and restrictive approach (no site-specific occupancy) where all discrete samples contribute equally to the calculation of occupancy, expressed as a proportion of 1 (or a percentage out of 100%), returning only those ASVs that are detected in every sample, and is sometimes biased towards more abundant ASVs; and a site specific approach, where occupancy is viewed as a detection within a particular location (province of residence) or type (gender), such that as long as the ASVs is represented in each province of residence/gender (not necessarily in all replicates within that province), it is counted as occurring there. The latter approach returned ASVs that are prone to return false-positive core ASVs, however, on average, it picks up medium to low abundant, and low occupancy ASVs. We then used the neutral modelling approach to partition these core ASVs to those that are neutral, and those that are above/below the model fit (deterministically assembled).

#### Covariates associated with microbial community distribution

To find the relationship between microbial communities and all sources of variation, including dietary patterns (as self-reported by subjects in filled questionnaires, given at the end of this document), we have used *Generalised Linear Latent Variable Model* (GLLVM) [92] which extends the basic generalized linear model that regresses the mean abundances of microbes against all sources of variation, even those that are not directly observed, as confounding latent variables. GLLVM extends the basic generalized linear model that regresses the mean abundances ${\mu }_{ij}$ (for $i$-th sample and $j$-th microbe) against covariates ${x}_{i}$ by incorporating latent variables ${u}_{i}$ as $g\left({\mu }_{ij}\right)={\eta }_{ij}={\alpha }_{i}+{\beta }_{0j}+{{\varvec{x}}}_{i}^{T}{{\varvec{\beta}}}_{j}+{{\varvec{u}}}_{i}^{T}{{\varvec{\theta}}}_{j}$, where ${{\varvec{\beta}}}_{j}$ are the microbe specific coefficients associated with individual covariates. Once estimated, a 95% confidence interval of these coefficients, whether positive or negative, and not crossing 0 gives directionality, i.e., the interpretation that an increase or decrease (if the covariate is categorical in nature then we use the word “inclusion”) of that particular covariate causes an increase or decrease in the abundance of the microbe). and ${{\varvec{\theta}}}_{j}$ are the corresponding coefficients associated with latent variable. ${\beta }_{0j}$ are microbes specific intercepts, whilst ${\alpha }_{i}$ are optional sample effects which can either be chosen as fixed effects or random effects. To model the distribution of individual microbes, we have used *Negative Binomial distribution* with an additional dispersion parameter, and using log() as a link function. Additionally, the approximation to the log-likelihood is done through Variational approximation (VA) with final sets of parameters in glvmm() function being family = ‘negative.binomial’, method = “VA”, and control.start = list(n.init = 7, jitter.var = 0.1)that seemed to fit well. This, we did for top 100 most abundant genera. In addition, the factor loadings ${{\varvec{\theta}}}_{j}$ store correlations of microbes with the residual covariance matrix $\boldsymbol{\Sigma }=\boldsymbol{\Gamma }{\boldsymbol{\Gamma }}^{T}$ where $\boldsymbol{\Gamma }=[{\theta }_{1}\dots {\theta }_{m}]$ for $m$ latent variables. This residual covariance matrix gave co-occurrence relationship between microbes that is not explained by the observed covariates.

#### Contingency analysis

To analyses the self-reported questionnaires, and to see if any two categorical covariates have a relationship, we constructed a contingency table and used ${\chi }^{2}$ test of independence using chisq.test() function in R. Based on recommendations given in http://www.sthda.com/english/wiki/chi-square-test-of-independence-in-r, and where the ${\chi }^{2}$ test was significant, we then calculated the ${\chi }^{2}$ residuals for individual rows and columns of the contingency table. These were drawn using R’s corrplot [93] package where positive values in cells specify an attraction (positive association; blue) between the corresponding row and column variables whilst negative values implies a repulsion (negative association; red) between the corresponding row and column variables. Additionally, we fitted a generalised linear model using glm() function using Freq ~ A + B model on the contingency table’s observed frequencies contingent upon all the factors for two covariates A, and B, and fitted using *Poisson* distribution. This then gave us incidence rate ratios for factors that were found to be significant.

### Supplementary Information

**Additional file 1: Figs. S1**–**S4** and **Tables S1**, **S2**.

**Additional file 2.** The raw data for core microbiome analysis shown in Fig. 1 which includes detailed taxonomic assignment of core ASVs along with occupancy specific information on different sheets.

**Additional file 3.** Meta data accompanying samples uploaded to the ENA repository PRJEB59240, and contains demographics, dietary, sleeping patterns, and routine life style information.

## Data Availability

The dataset presented in this study is available under ENA repository PRJEB59240 with the meta data provided in the Sample_Details.xlsx. The Additional file 1: materials, figures, and tables are shown in Additional file 1: with additional datasets provided as Sample_Details.xlsx in Additional file 3 and Core_Microbiome_Details.xlsx in Additional file 2.

## Abbreviations

ASV: Amplicon sequencing variants
GLLVM: Generalized linear latent variable model
SCFA: Short-chain fatty acid
LMIC: Lower middle income country
IBD: Inflammatory bowel disease
HMP: Human microbiome project
rRNA: Ribosomal RNA
PERMANOVA: Permutation ANOVA
ICT: Islamabad Capital Territory
AJK: Azad Jammu & Kashmir
KPK: Khyber Pakhtunkhwa
LC: Lower class
LMC: Lower middle class
MC: Middle class
UMC: Upper middle class
HMS: Hierarchical meta-storms
PCoA: Principal coordinate analysis
MiCA: Microbiota Centre of Amsterdam
NSTI: Nearest Sequenced Taxon Index
SSU: Short subunit
RPM: Revolutions per minute
RCF: Relative centrifugal force
FMT: Fecal microbiota transplant
