# Supplementary material for: Gut microbial ecology and exposome of a healthy Pakistani cohort
Source: Gut Pathog. 2024 Jan 22;16:5. doi: 10.1186/s13099-024-00596-x (PMC10801943; doi:10.1186/s13099-024-00596-x)
Supplement: Supplementary file 1 — Additional file 1: Figs. S1–S4 and Tables S1, S2. [file 13099_2024_596_MOESM1_ESM.pdf]

Supplementary Materials for

Gut microbial ecology and exposome of a healthy Pakistani cohort

Farzana Gul[1], Hilde Herrema[2], Mark Davids[2], Ciara Keating[3], Arshan Nasir[1, †], Umer Zeeshan Ijaz[4,5,6, *], Sundus Javed[1, *]

[1]Department of Biosciences, COMSATS University Islamabad, Islamabad 45550, Pakistan
[2]Department of Experimental Vascular Medicine, Amsterdam University Medical Centers, Location AMC, Amsterdam, the Netherlands
[3]School of Biodiversity, One Health & Veterinary Medicine, Graham Kerr Building, University of Glasgow, Glasgow G12 8QQ, UK
[4]Water & Environment Research Group, University of Glasgow, Mazumdar-Shaw Advanced Research Centre, Glasgow G11 6EW, UK
[5]Department of Molecular and Clinical Cancer Medicine, University of Liverpool, Liverpool L69 7BE, UK
[6]National University of Ireland, Galway, University Road, Galway, H91 TK33, Ireland
[†]Present address: Moderna, Inc., Cambridge, MA, USA.

***Joint corresponding author**
Email: sundus.javed@comsats.edu.pk
Email: Umer.Ijaz@glasgow.ac.uk (http://userweb.eng.gla.ac.uk/umer.ijaz)

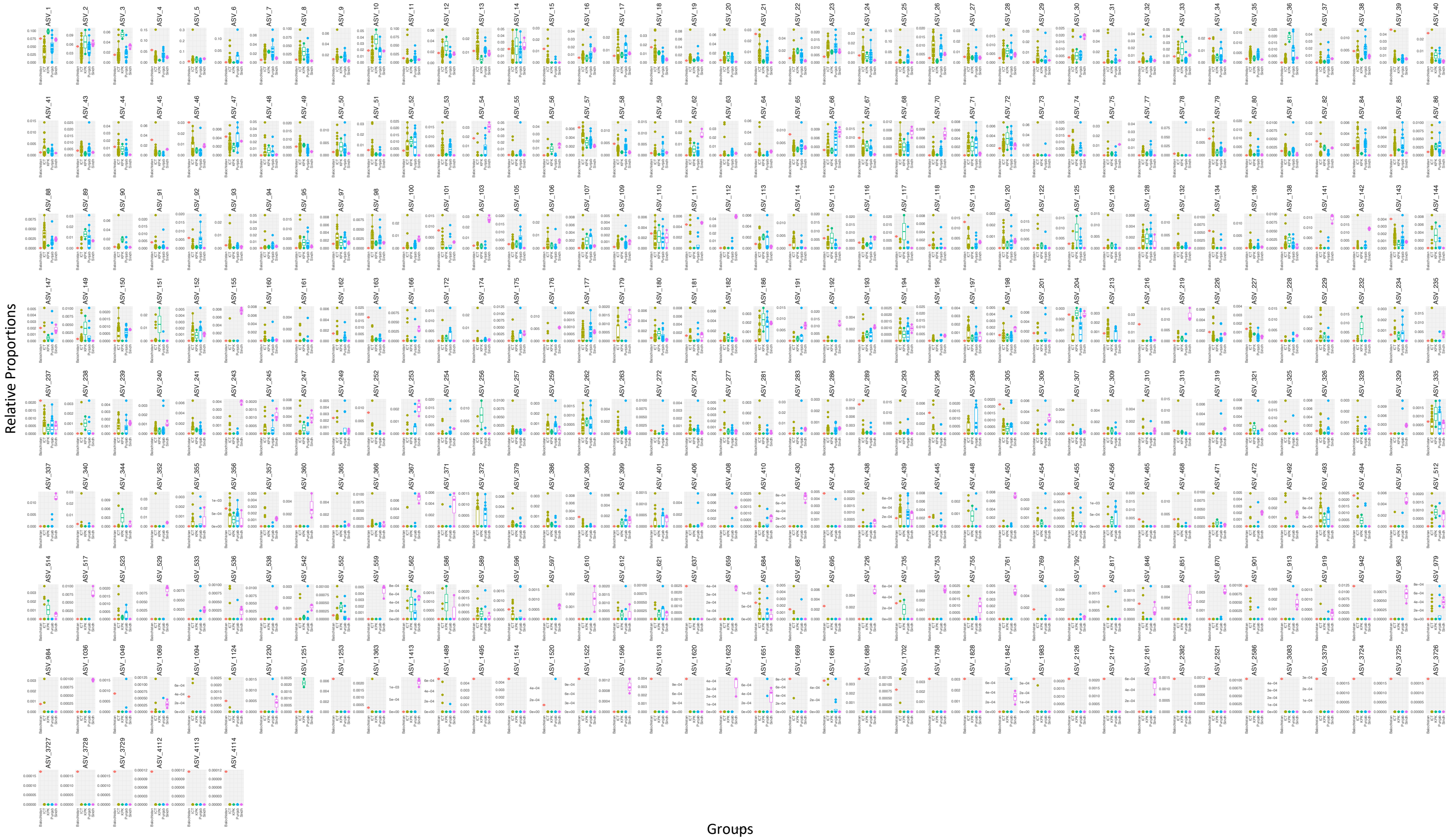

Fig. S1. Core Microbiome of Pakistani males with *Province of Residence* specific occupancy. The taxonomic information of ASVs is given in Core_Microbiome_Details.xlsx.

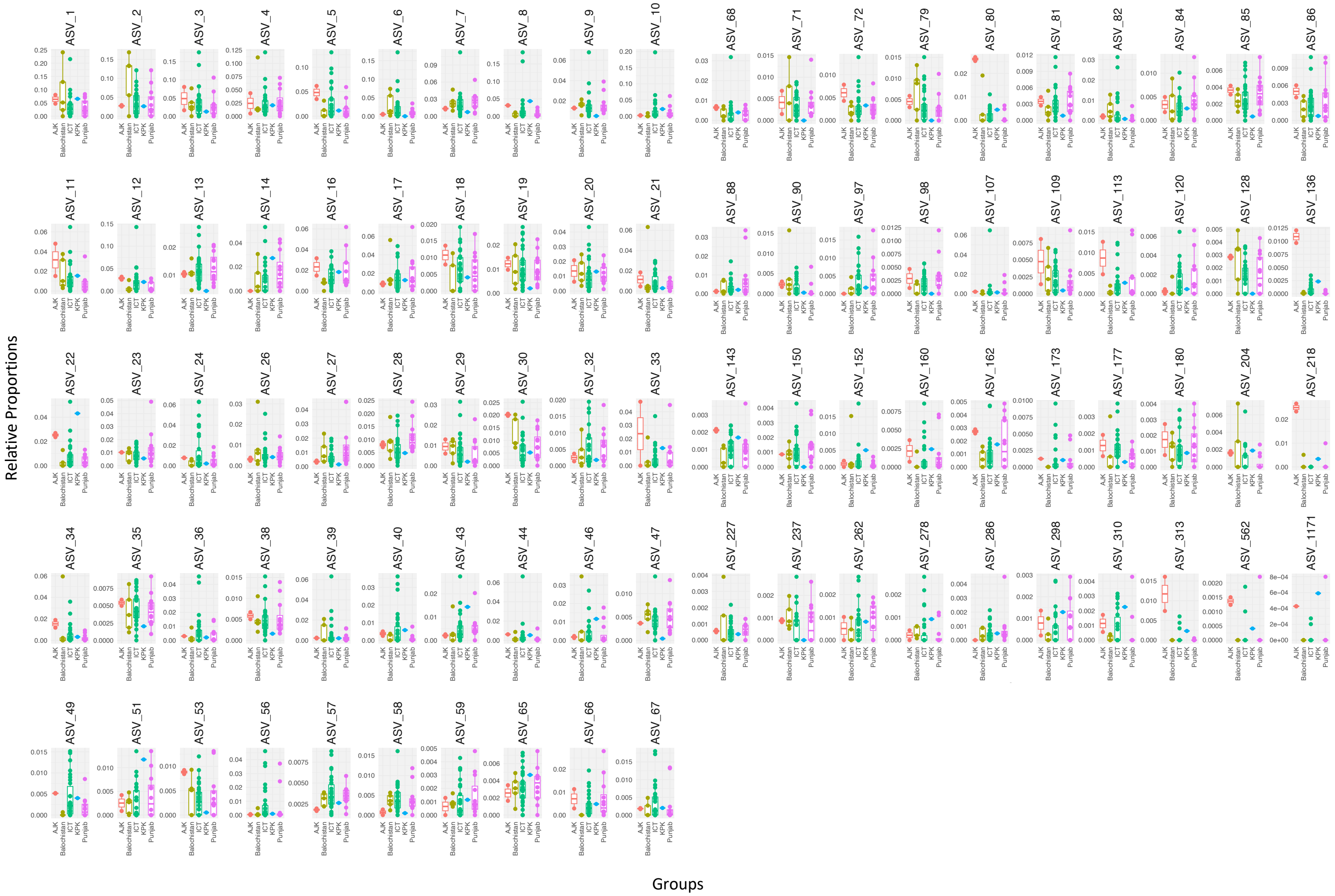

Fig. S2. Core Microbiome of Pakistani females with *Province of Residence* specific occupancy. The taxonomic information of ASVs is given in Core_Microbiome_Details.xlsx.

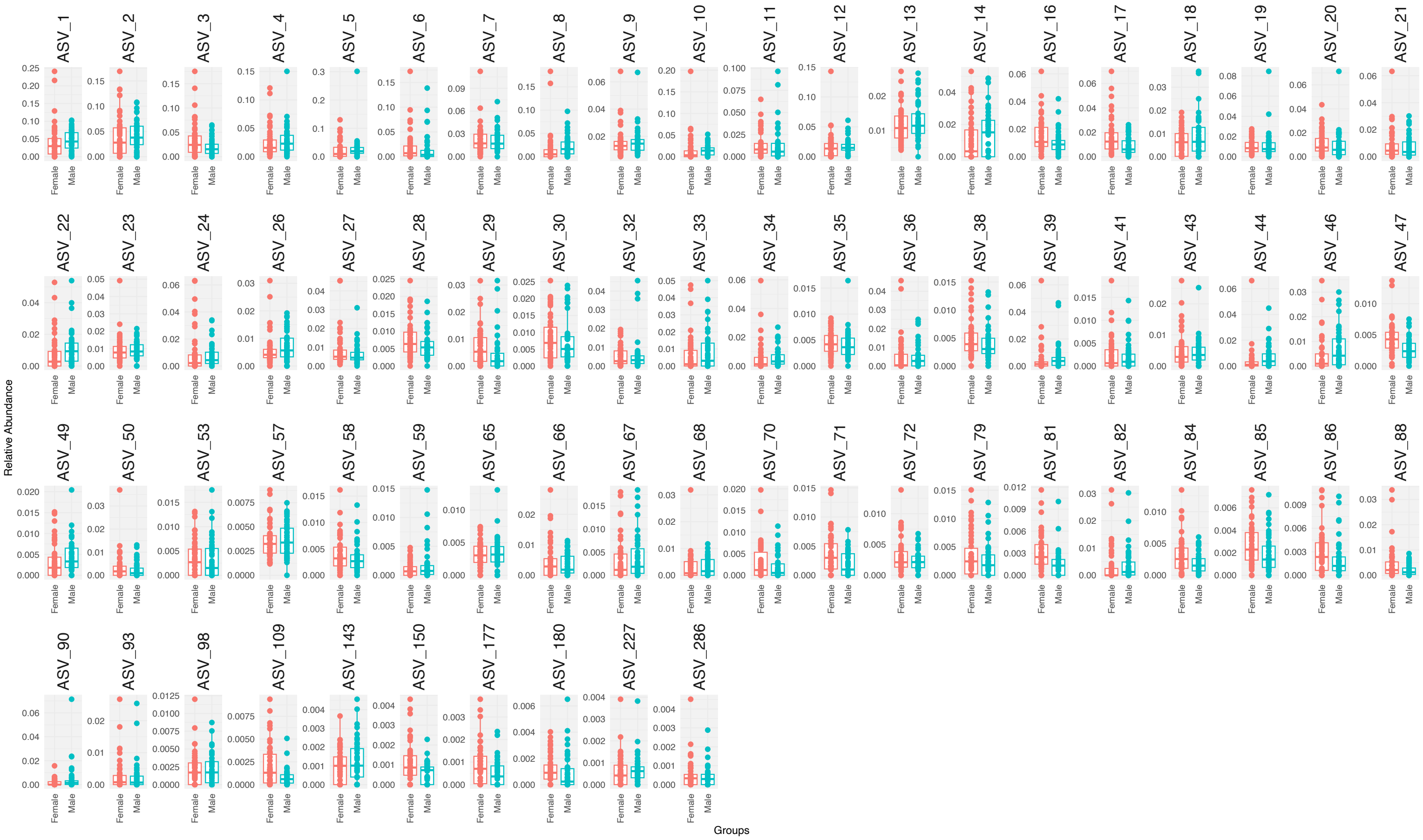

Fig. S3. Core Microbiome of Pakistani individuals with *Gender* specific occupancy. The taxonomic information of ASVs is given in Core_Microbiome_Details.xlsx

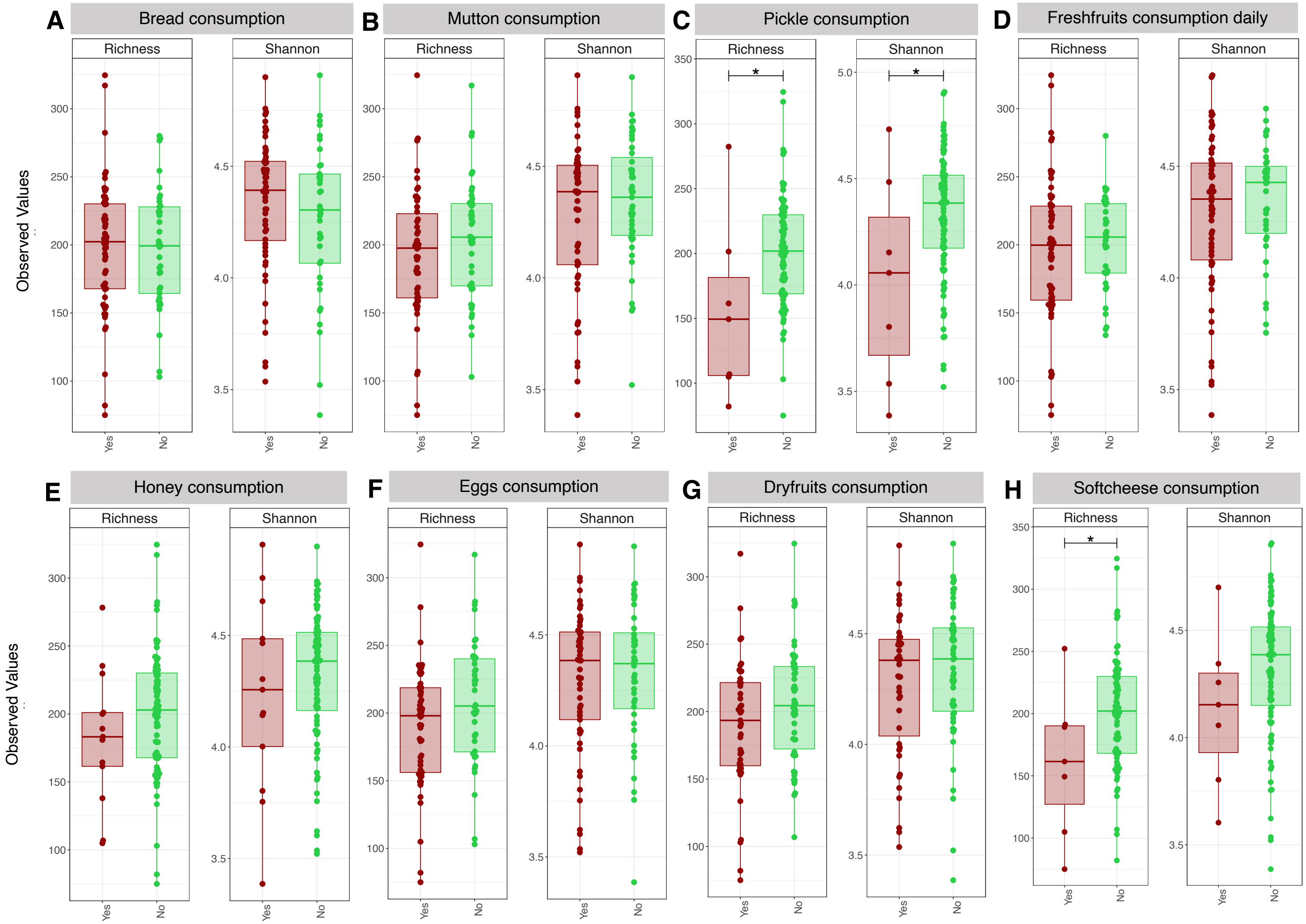

Fig. S4: Alpha diversity comparison of microbial community composition (bacterial ASV abundances) of samples for different sources of variability based on the self-reported questionnaire. The lines connect samples according to ANOVA with the significance values as: * p < 0.05, ** p < 0.01, or *** p < 0.001.

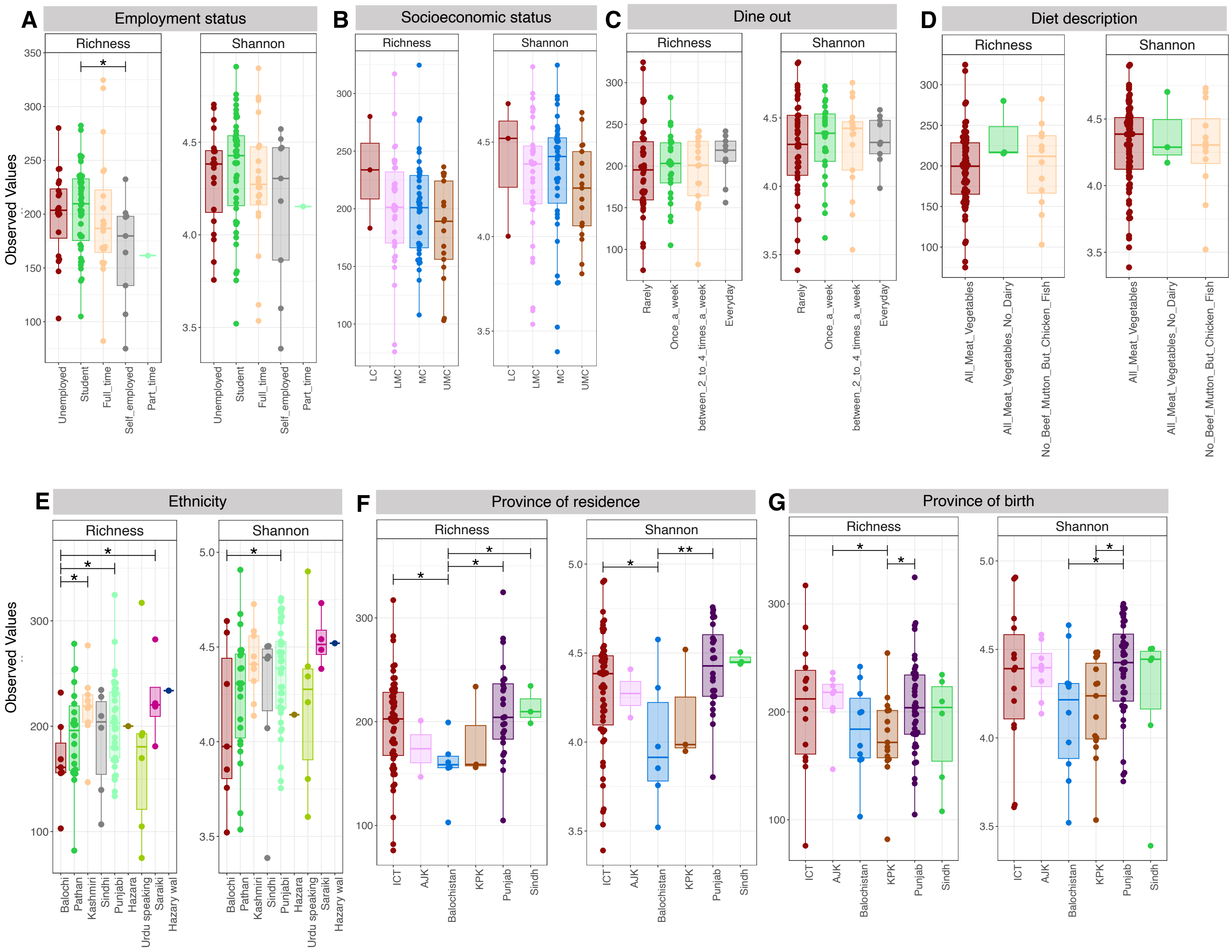

Fig. S5: Continuation of alpha diversity comparison of microbial community composition (bacterial ASV abundances) of samples for different sources of variability. See the legend of Fig. S4 for details.

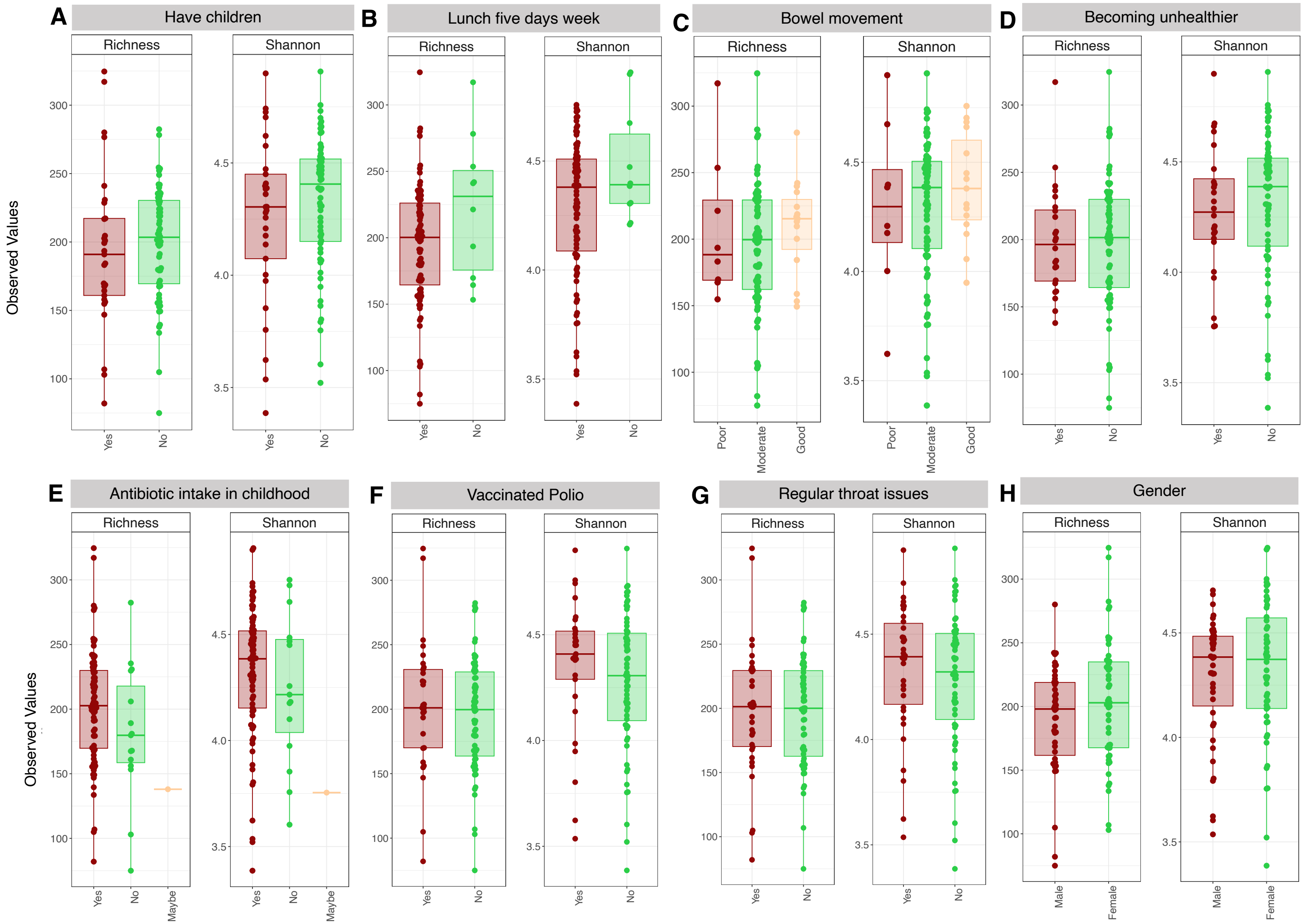

Fig. S6: Continuation of alpha diversity comparison of microbial community composition (bacterial ASV abundances) of samples for different sources of variability. See the legend of Fig. S4 for details.

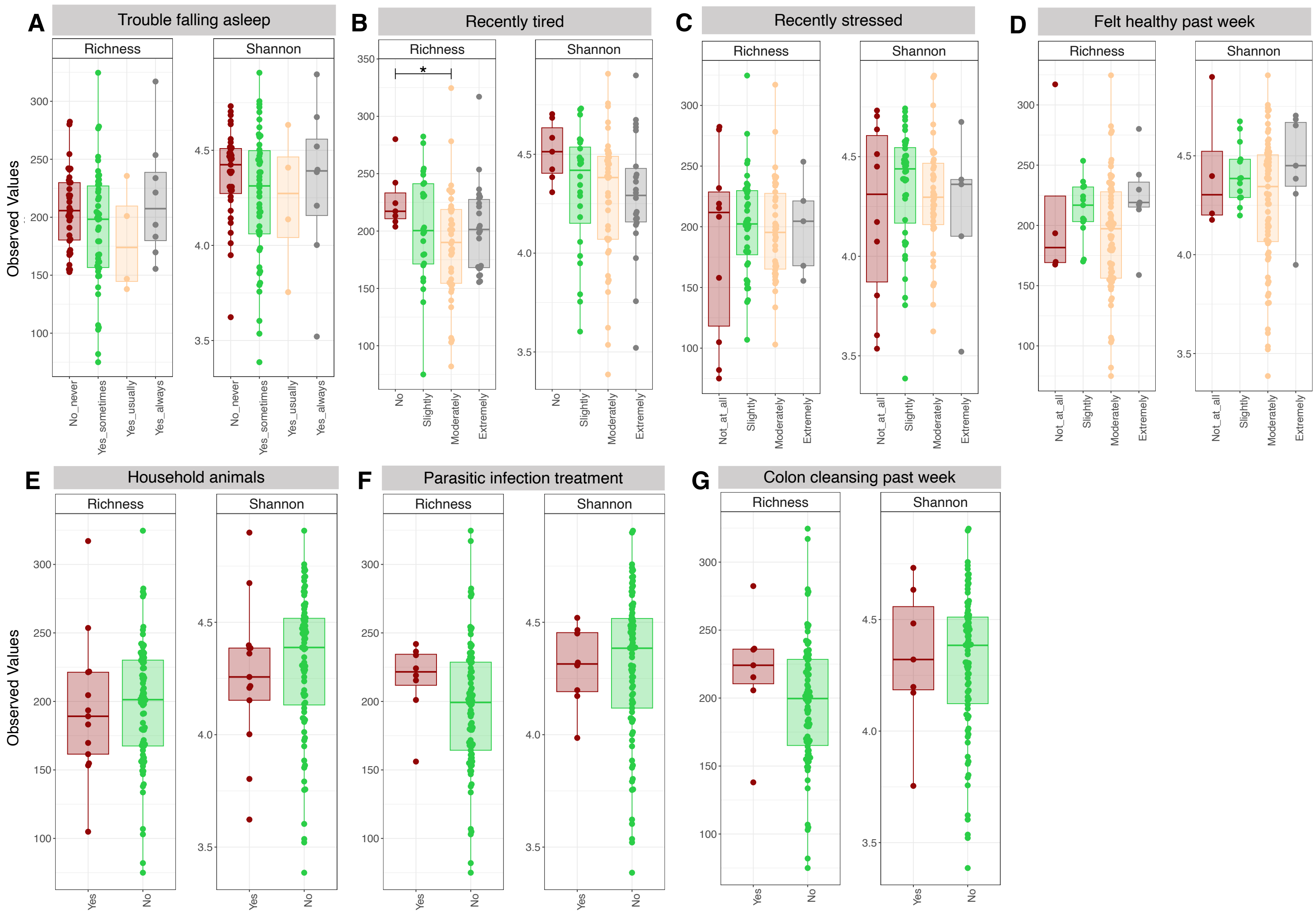

Fig. S7: Continuation of alpha diversity comparison of microbial community composition (bacterial ASV abundances) of samples for different sources of variability. See the legend of Fig. S4 for details.

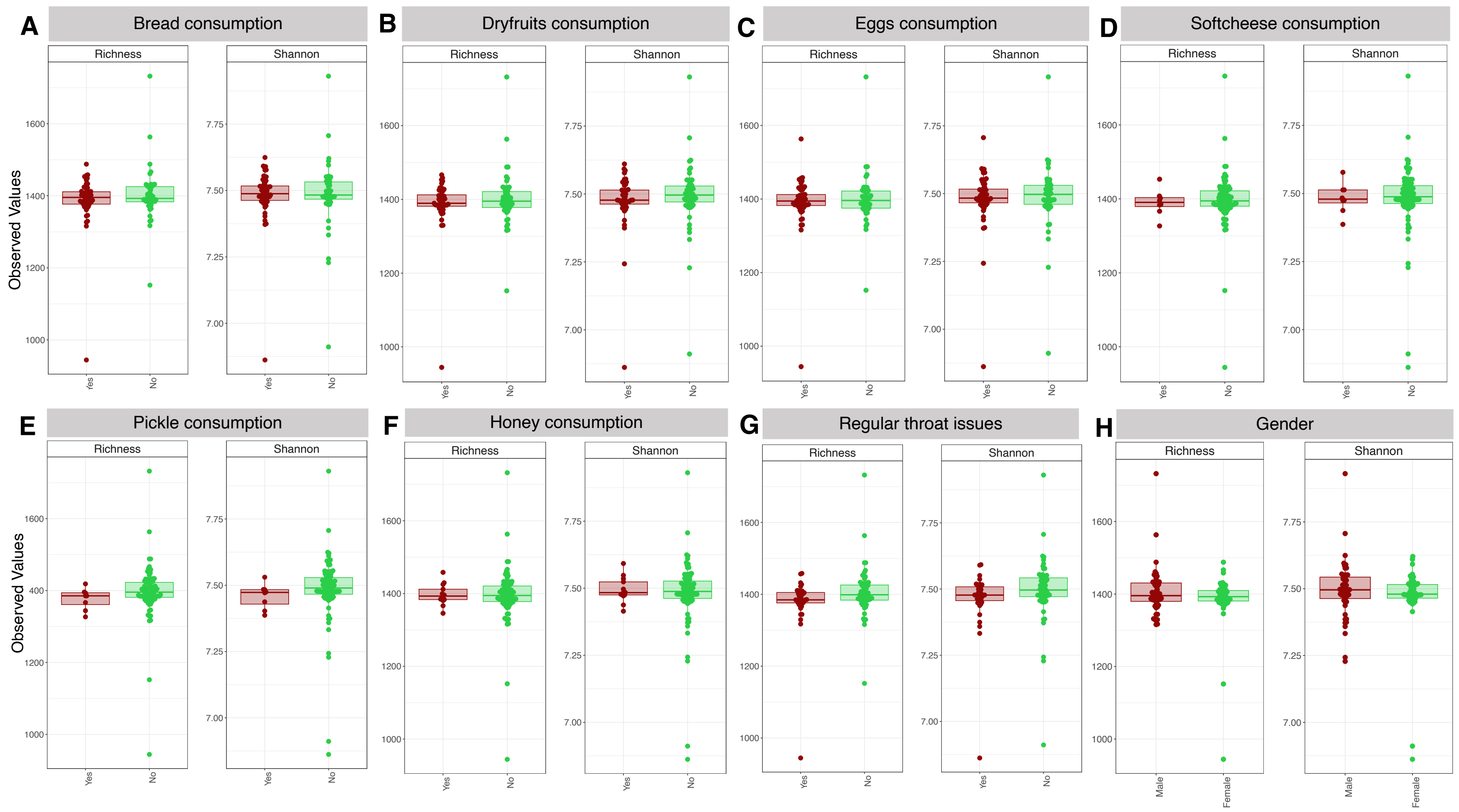

Fig. S8: Alpha diversity comparison of predicted metabolic function (KEGG KO abundances) of samples for different sources of variability based on the self-reported questionnaire. The lines connect samples according to ANOVA with the significance values as: * $p < 0.05$, ** $p < 0.01$, or *** $p < 0.001$.

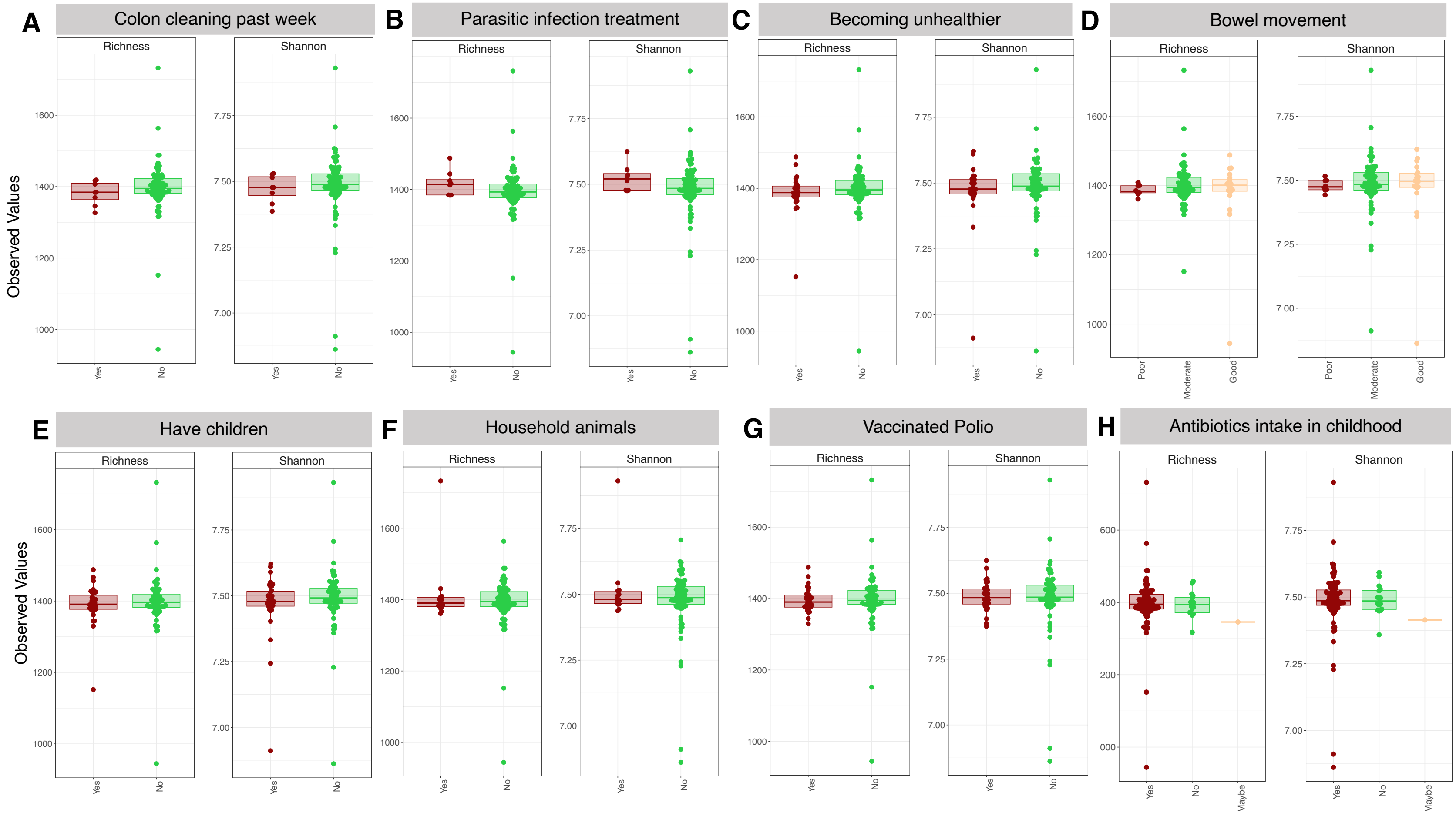

Fig. S9: Continuation of alpha diversity comparison of predicted metabolic function (KEGG KO abundances) of samples for different sources of variability. See the legend of Fig. S8 for details.

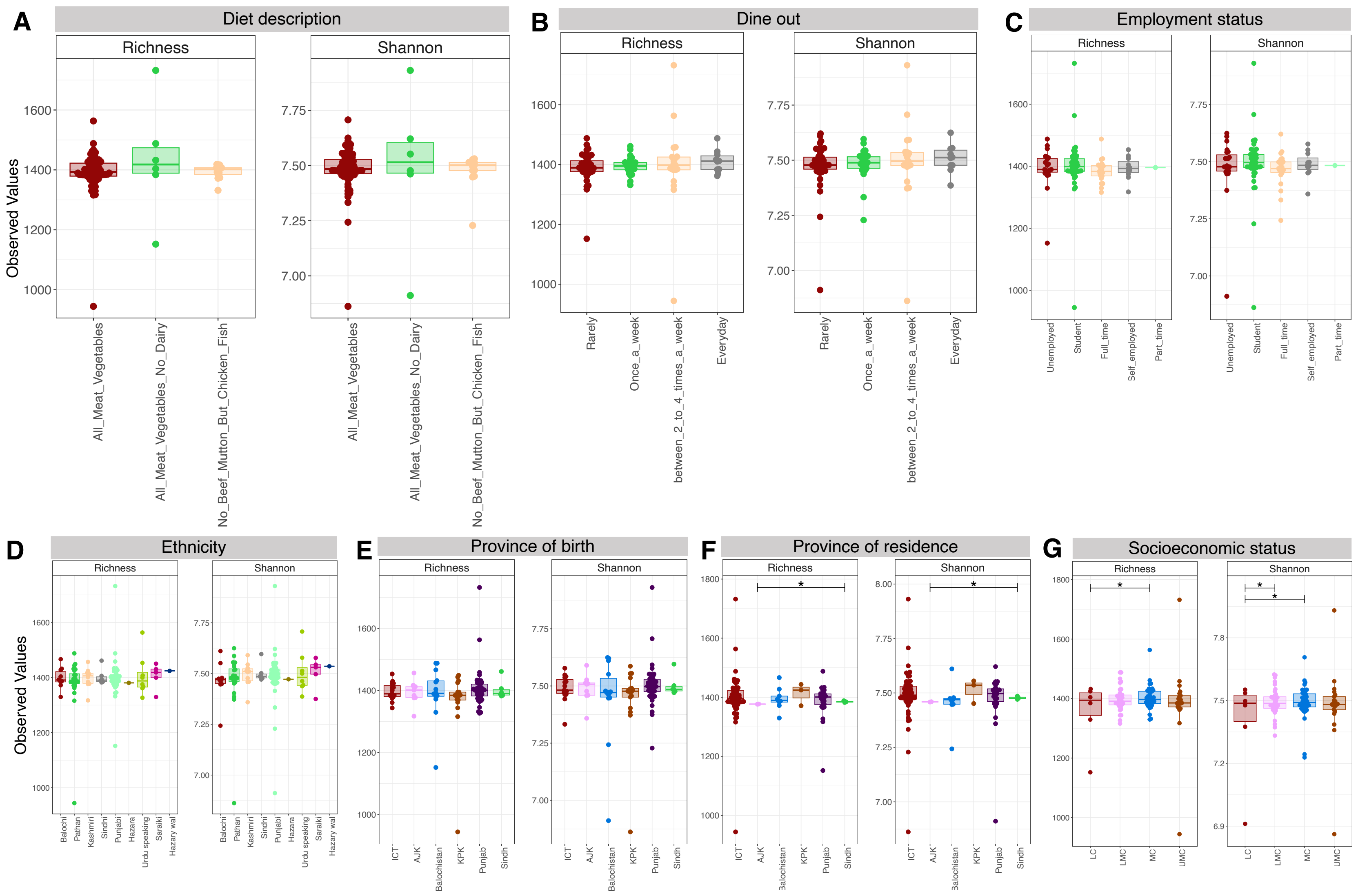

Fig. S10: Continuation of alpha diversity comparison of predicted metabolic function (KEGG KO abundances) of samples for different sources of variability. See the legend of Fig. S8 for details.

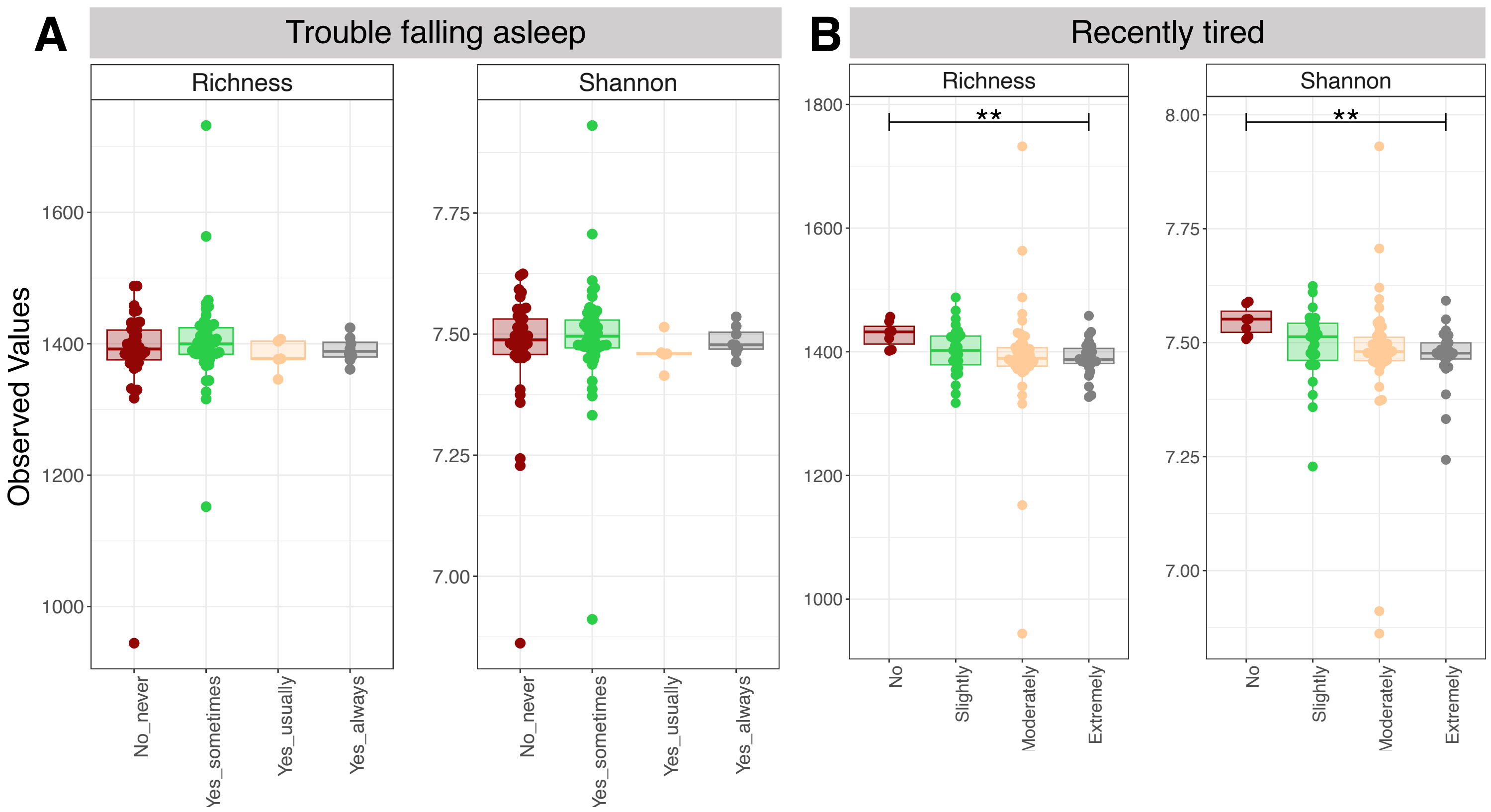

Fig. S11: Continuation of alpha diversity comparison of predicted metabolic function (KEGG KO abundances) of samples for different sources of variability. See the legend of Fig. S8 for details.

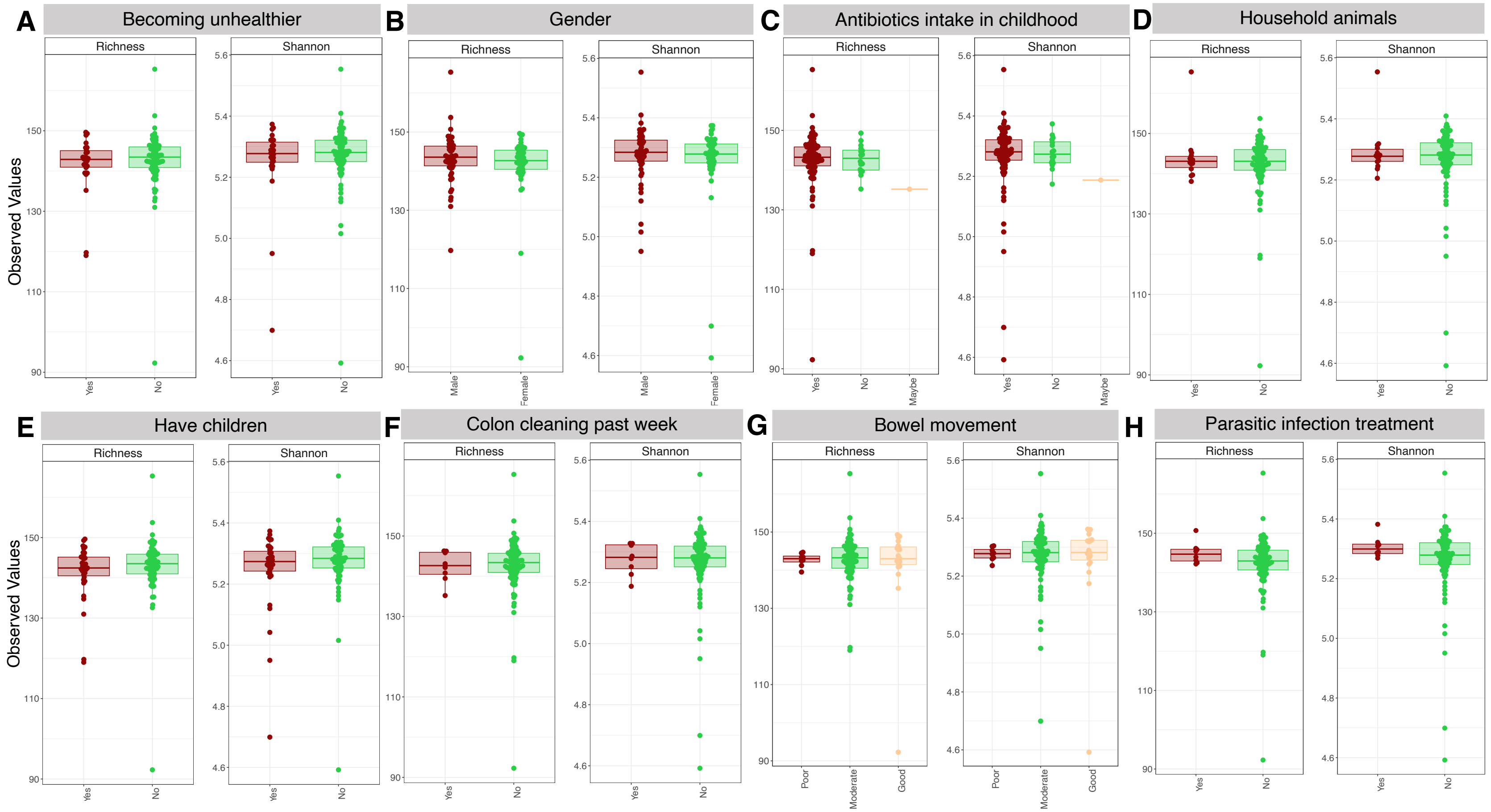

Fig. S12: Alpha diversity comparison of predicted metabolic function (MetaCyc pathway abundances) of samples for different sources of variability based on the self-reported questionnaire. The lines connect samples according to ANOVA with the significance values as: * $p < 0.05$, ** $p < 0.01$, or *** $p < 0.001$.

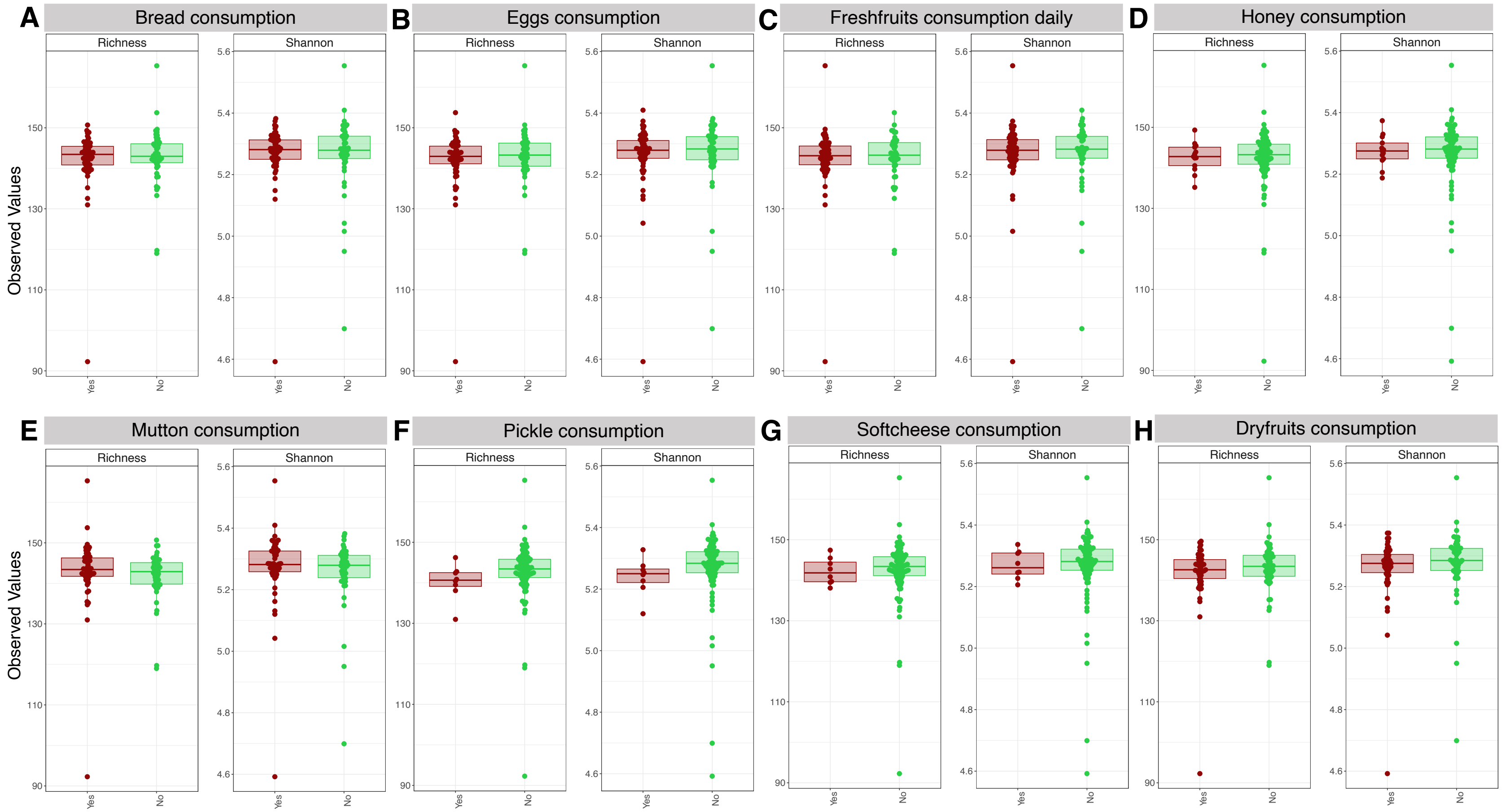

Fig. S13: Continuation of alpha diversity comparison of predicted metabolic function (MetaCyc pathway abundances) of samples for different sources of variability. See the legend of Fig. S12 for details.

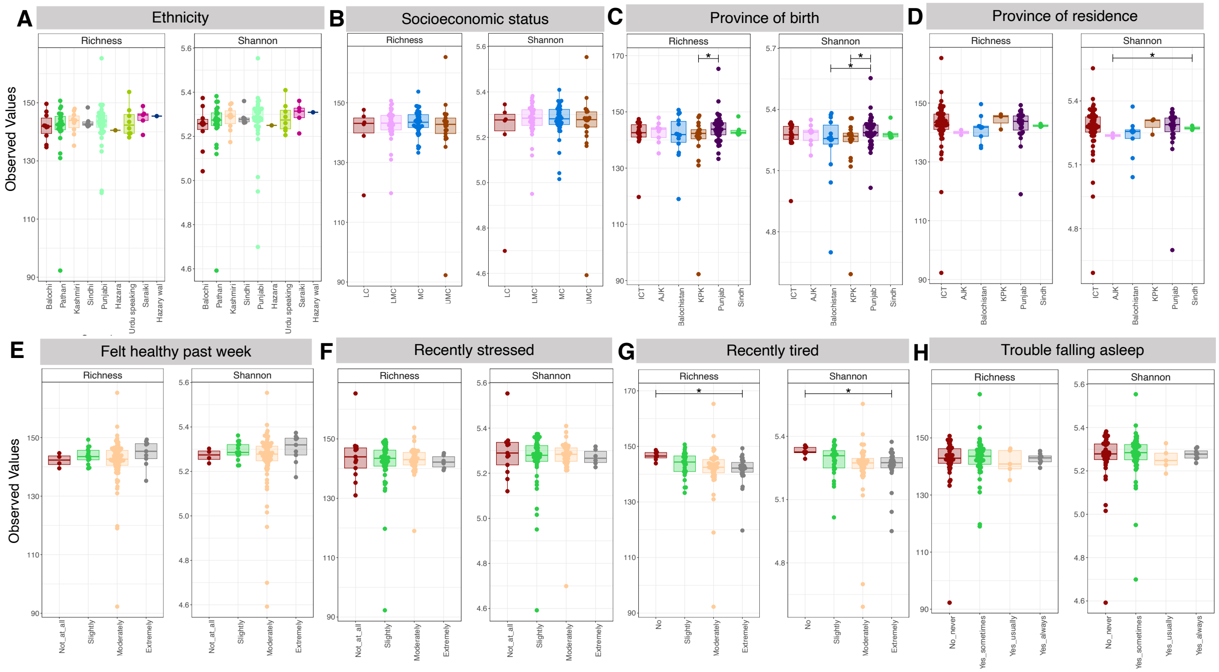

Fig. S14: Continuation of alpha diversity comparison of predicted metabolic function (MetaCyc pathway abundances) of samples for different sources of variability. See the legend of Fig. S12 for details.

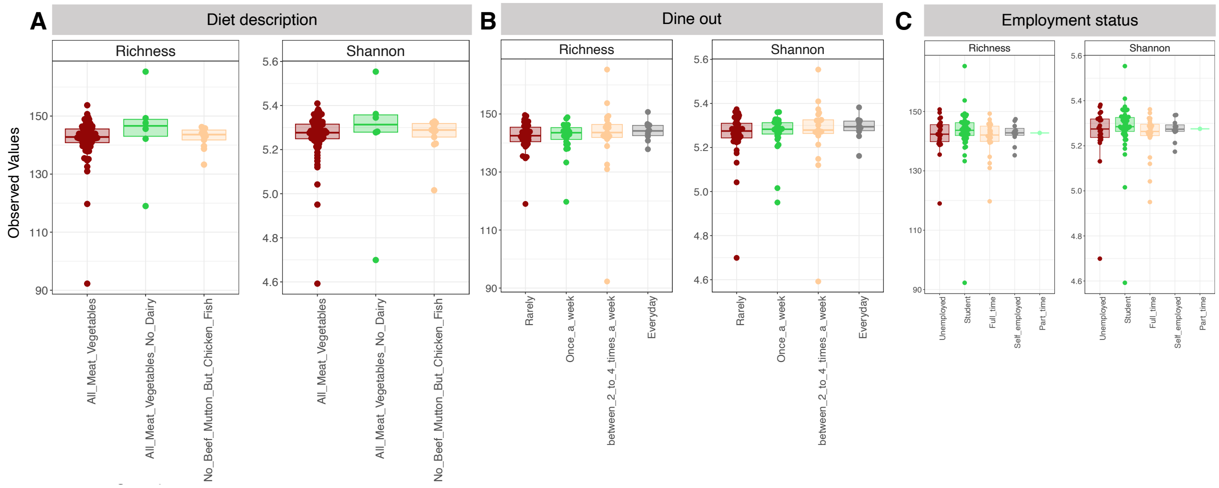

Fig. S15: Continuation of alpha diversity comparison of predicted metabolic function (MetaCyc pathway abundances) of samples for different sources of variability. See the legend of Fig. S12 for details.

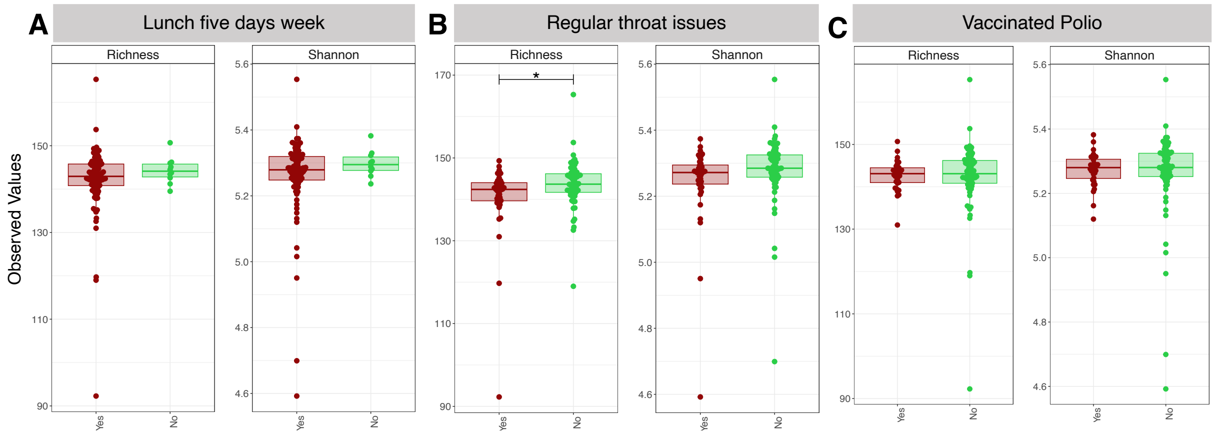

Fig. S16: Continuation of alpha diversity comparison of predicted metabolic function (MetaCyc pathway abundances) of samples for different sources of variability. See the legend of Fig. S12 for details.

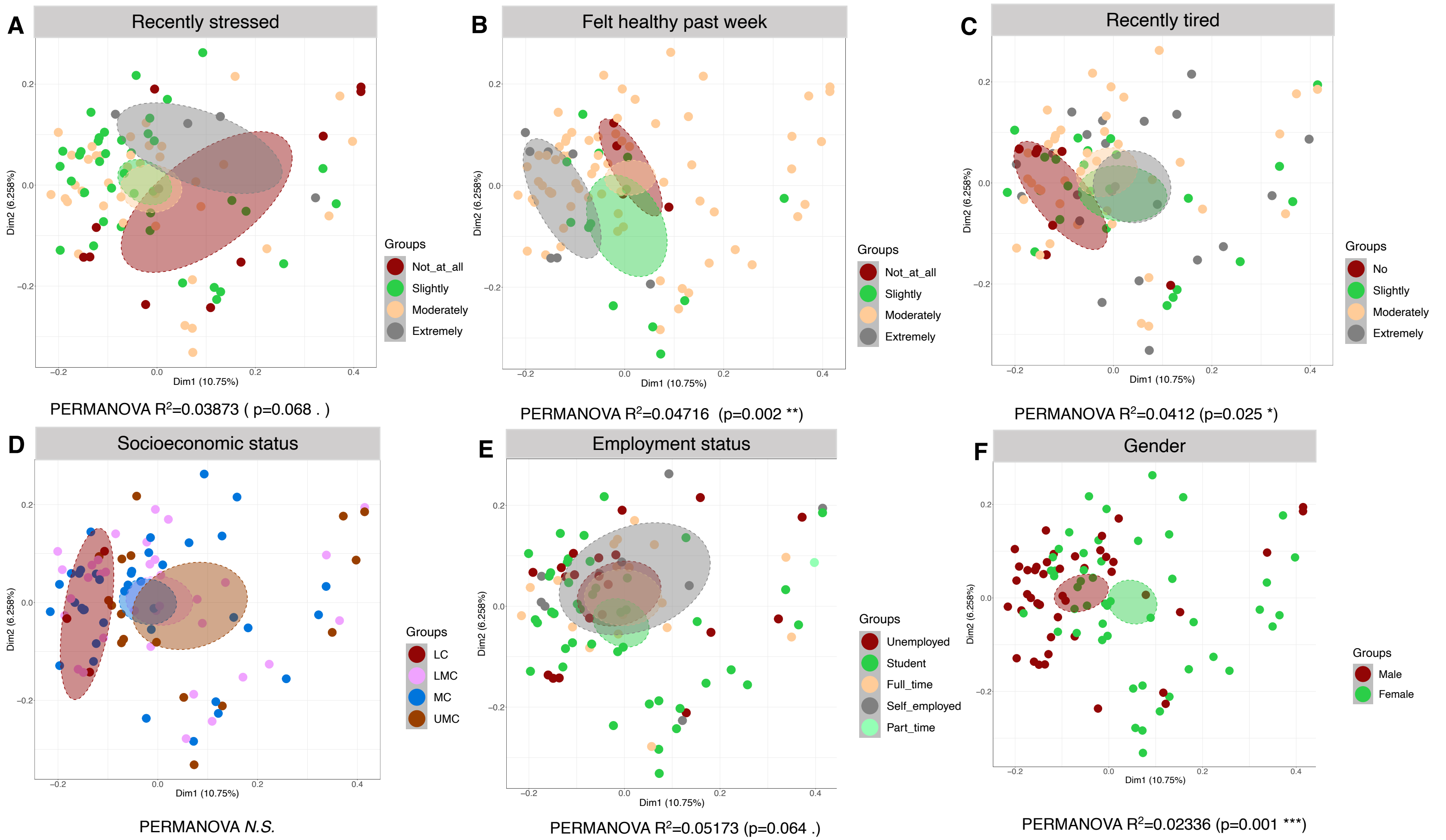

Fig. S17: Beta diversity comparison of microbial community composition (bacterial ASV abundances) of samples for different sources of variability based on the self-reported questionnaire. We have used Bray-Curtis distance in the Principle Coordinate Analysis (PCoA) plot where ellipses are drawn using 95% confidence intervals based on the standard error of spread of points for a given category. Beneath each figure are shown the R^2 values (along with p-values if significant) calculated from PERMANOVA and represents the percentage variability in microbiome explained between all possible values of the groups.

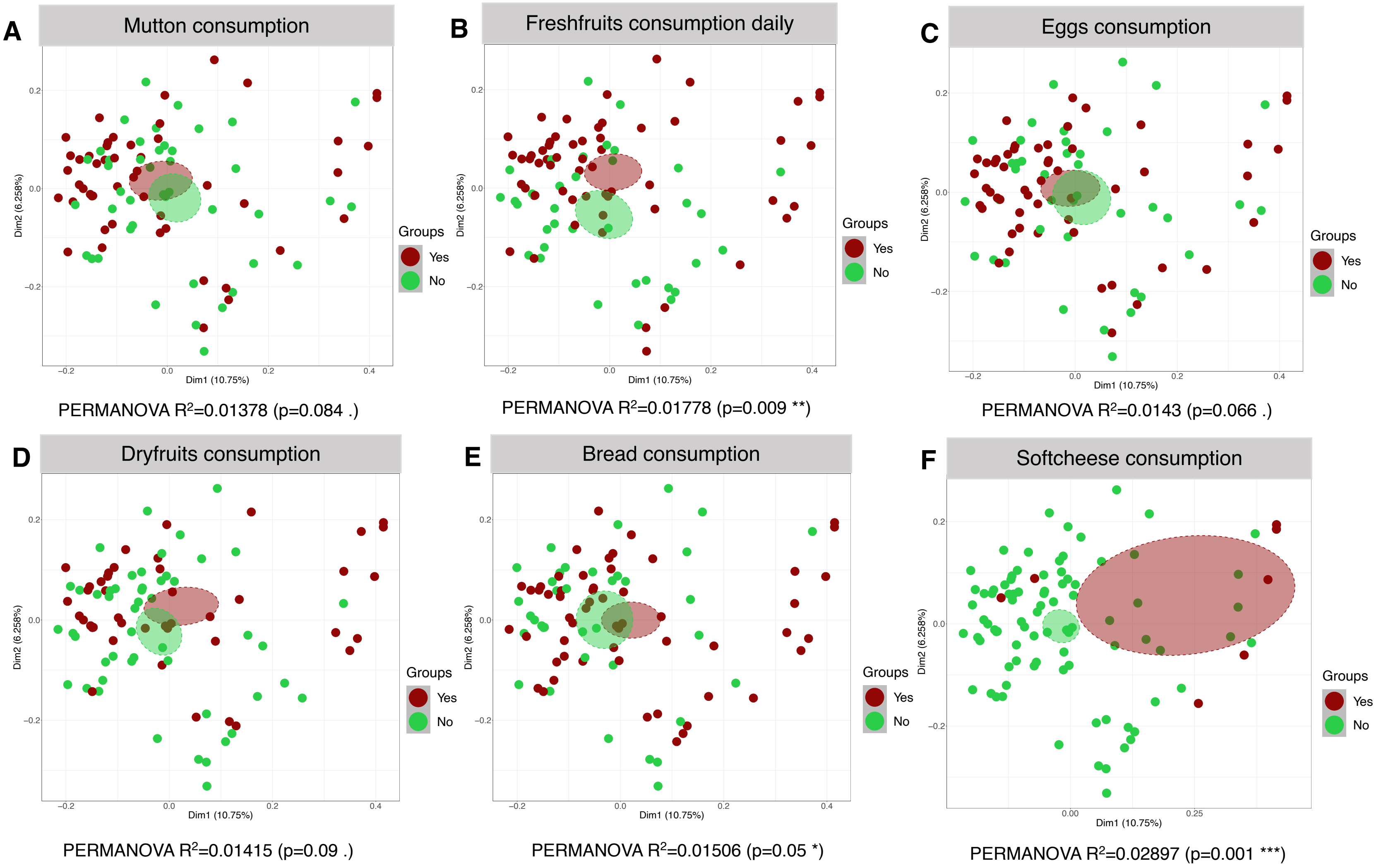

Fig. S18: Continuation of beta diversity comparison of microbial community composition (bacterial ASV abundances) of samples for different sources of variability. See the legend of Fig. S17 for details.

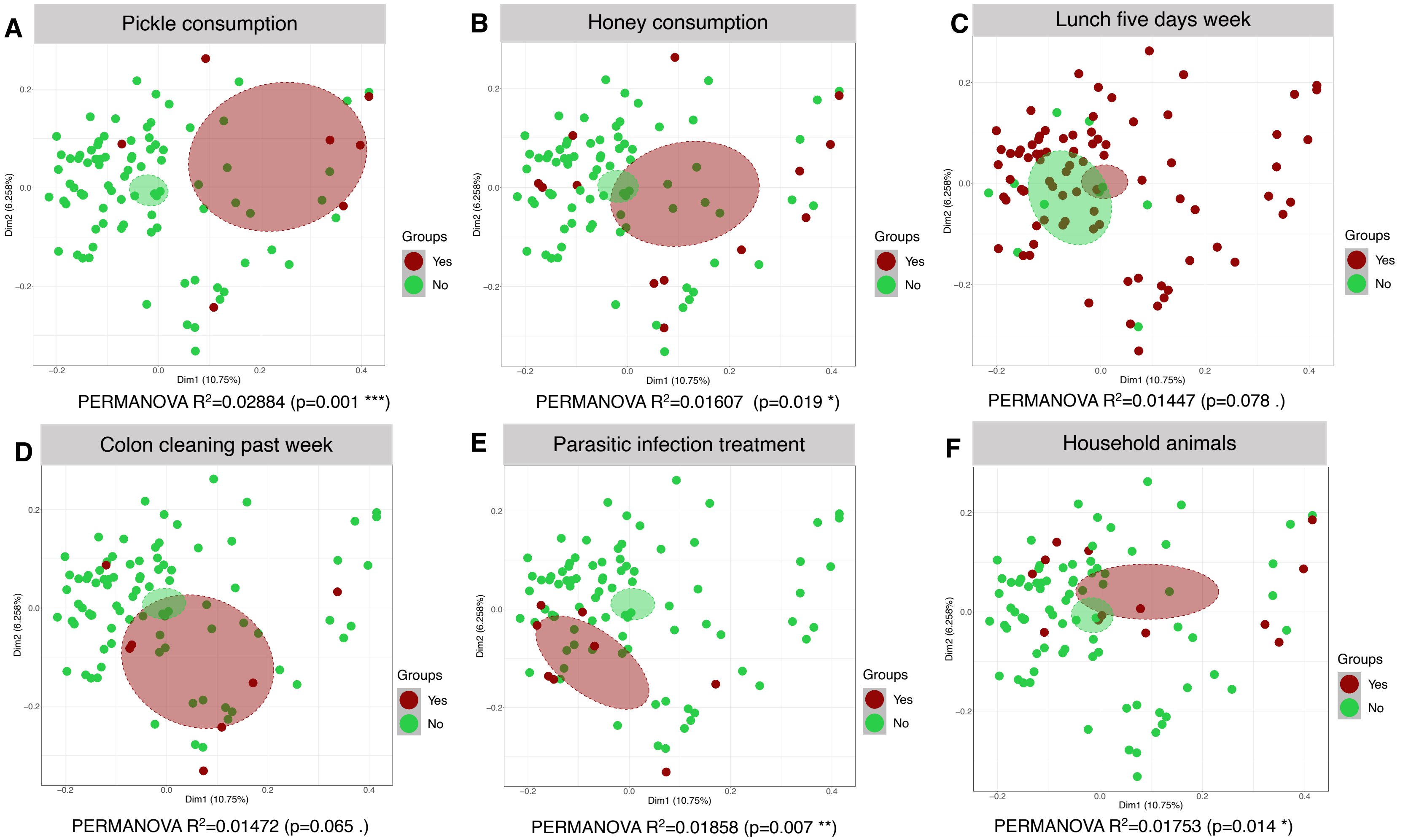

Fig. S19: Continuation of beta diversity comparison of microbial community composition (bacterial ASV abundances) of samples for different sources of variability. See the legend of Fig. S17 for details.

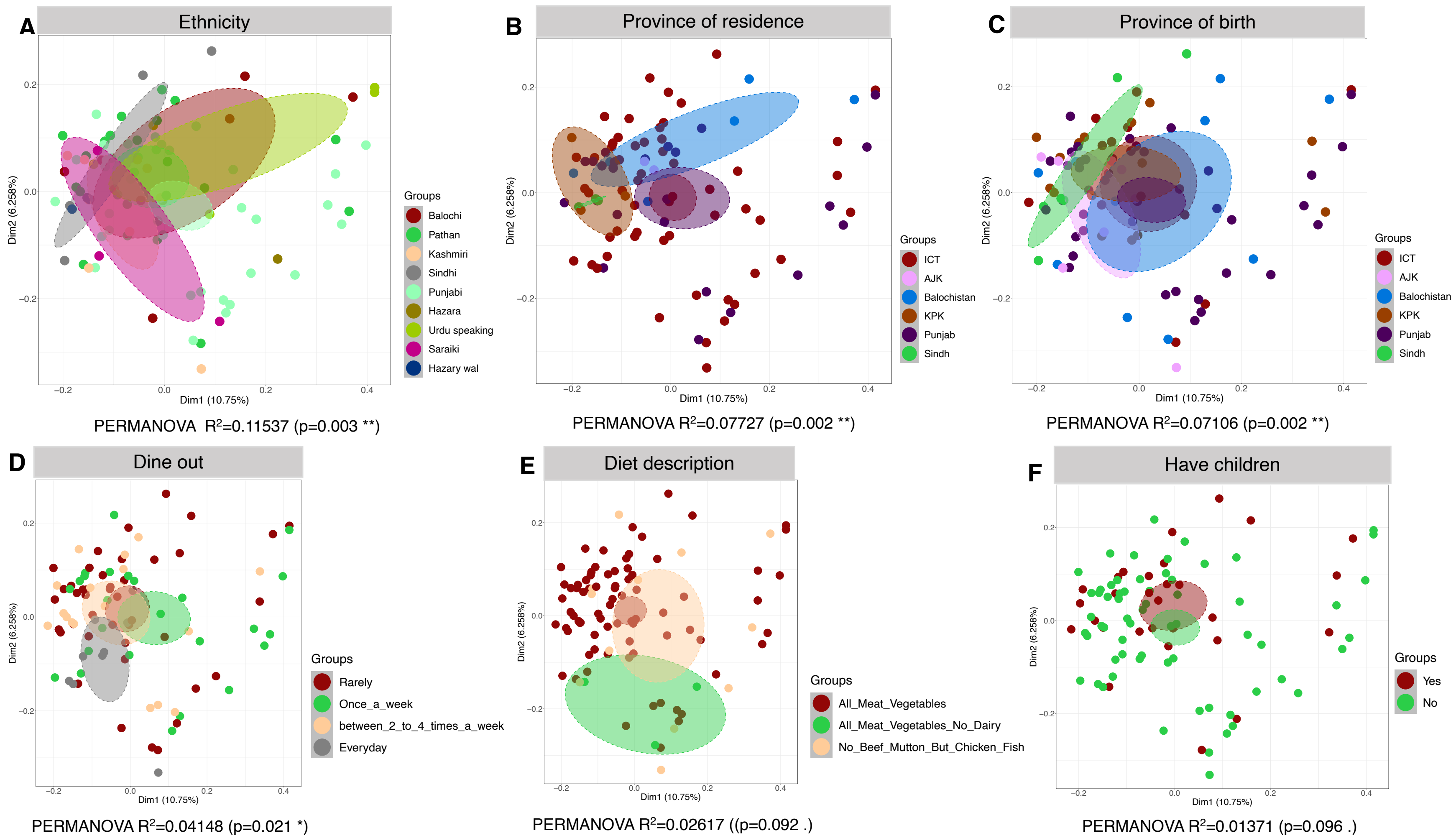

Fig. S20: Continuation of beta diversity comparison of microbial community composition (bacterial ASV abundances) of samples for different sources of variability. See the legend of Fig. S17 for details.

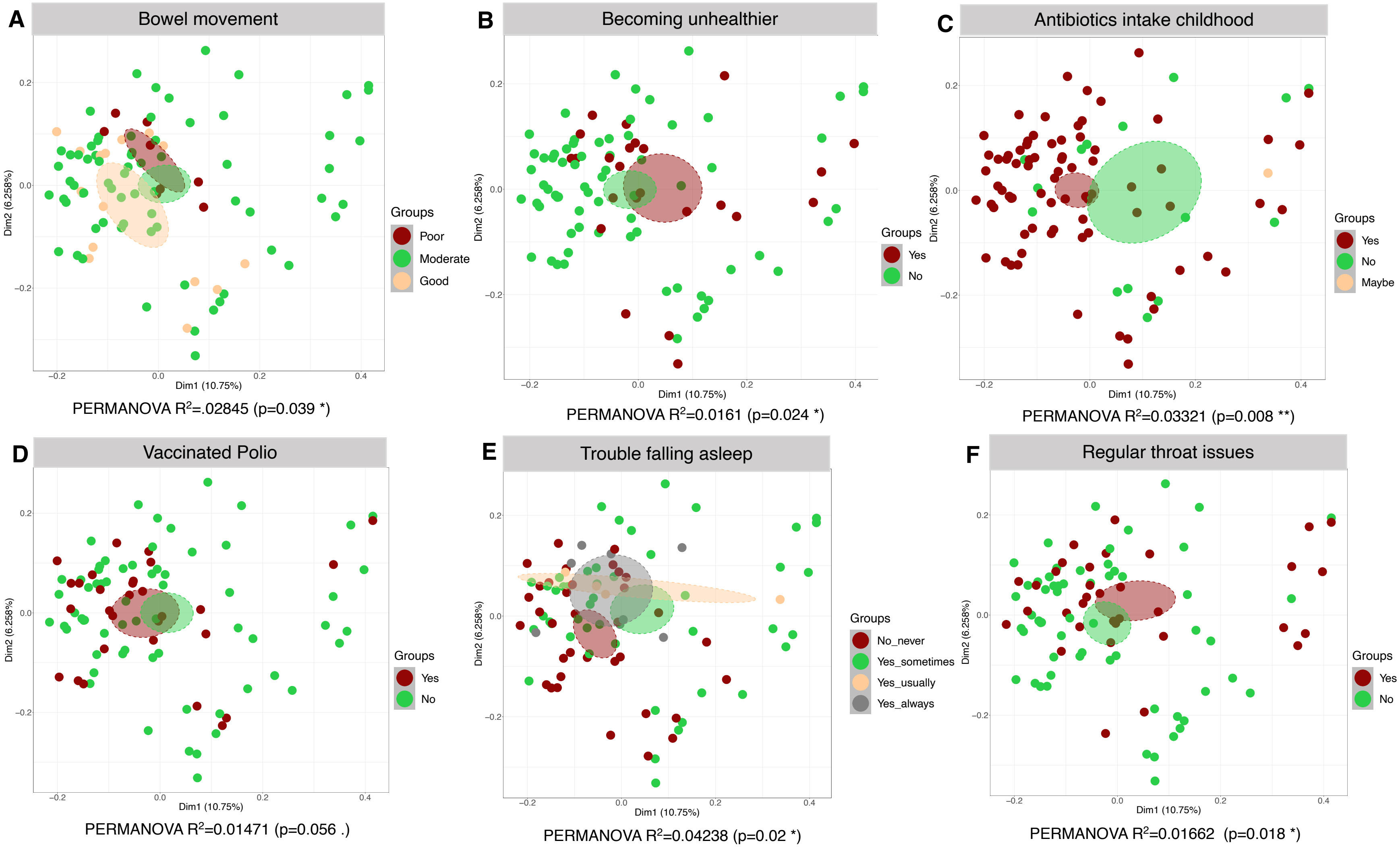

Fig. S21: Continuation of beta diversity comparison of microbial community composition (bacterial ASV abundances) of samples for different sources of variability. See the legend of Fig. S17 for details.

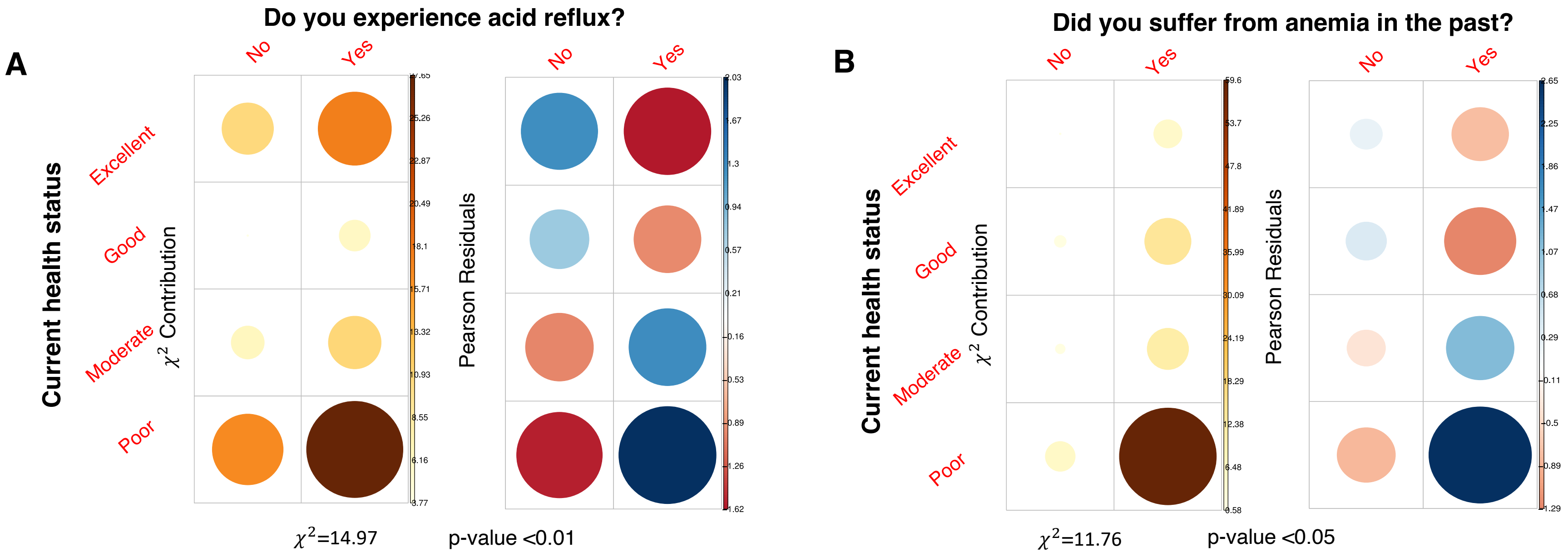

Predictors	Incidence Rate Ratios (95 % CI)
(Intercept)	4.37*** (1.86 – 8.58)
Current health status [Good]	9.43*** (4.64 – 22.60)
Current health status [Moderate]	5.71*** (2.73 – 13.95)
Current health status [Poor]	0.57 (0.15 – 1.89)
Acid reflux [Yes]	0.60** (0.41 – 0.87)
Observations	8
R^2	1.000

* p<0.05 ** p<0.01 *** p<0.001

Predictors	Incidence Rate Ratios (95 % CI)
(Intercept)	6.34*** (2.72 – 12.30)
Current health status [Good]	9.43*** (4.64 – 22.60)
Current health status [Moderate]	5.71*** (2.73 – 13.95)
Current health status [Poor]	0.57 (0.15 – 1.89)
Anemia [Yes]	0.10*** (0.05 – 0.18)
Observations	8
R^2	1.000

* p<0.05 ** p<0.01 *** p<0.001

Fig. S22: Significant dependences recovered between categorical-response questions in self-reported questionnaire using χ^2 test of independence. Where significant (p<0.05), the χ^2 contribution and Pearson residuals are calculated. The χ^2 contribution represent the biggest contributer to the χ^2 value whilst for Pearson residuals, a positive value, represented by the blue color, is a positive attraction between the corresponding row and column variables whilst negative values implie a repulsion (negative association; red) between the corresponding row and column variables. Additionally, we fitted a GLM model with frequency on the contingency table to calculate the incidence ratios and where significant they are highlighted in red.

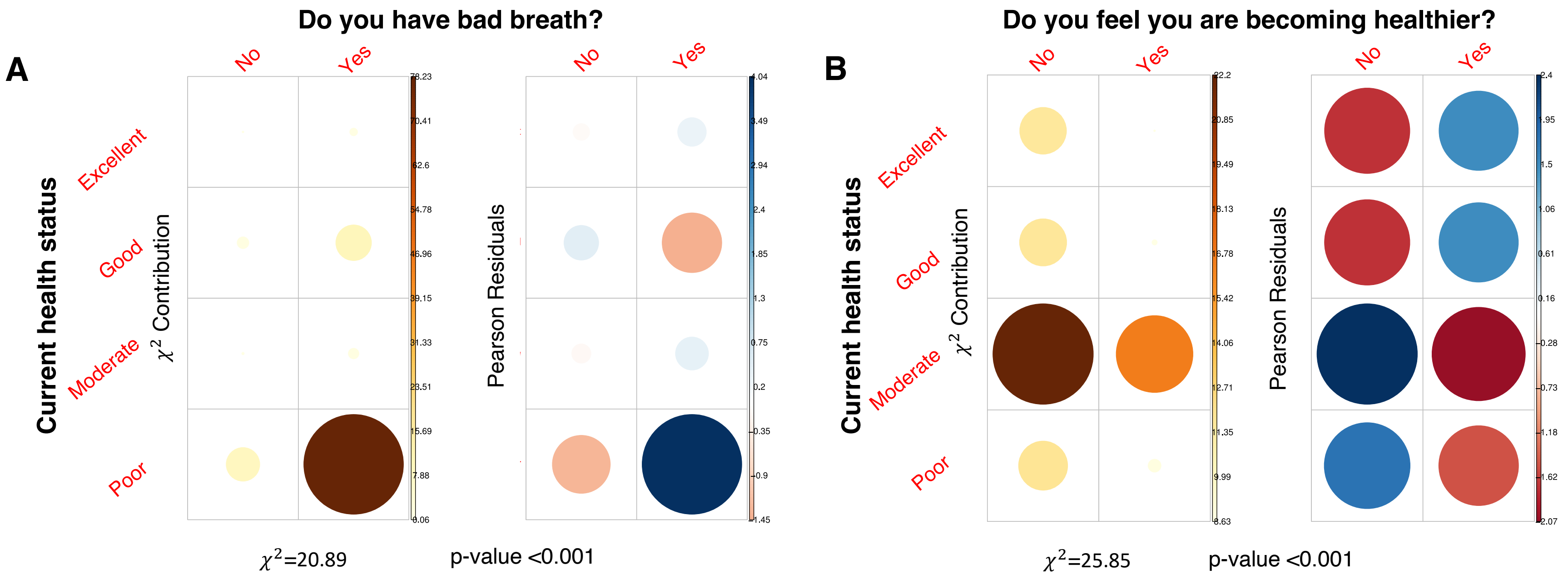

Predictors	Incidence Rate Ratios (95 % CI)
(Intercept)	6.28*** (2.69 – 12.19)
Current health status [Good]	9.43*** (4.64 – 22.60)
Current health status [Moderate]	5.71*** (2.73 – 13.95)
Current health status [Poor]	0.57 (0.15 – 1.89)
Bad breath [Yes]	0.11** (0.06-0.20)
Observations	8
R^2	1.000
	* p<0.05 ** p<0.01 *** p<0.001

Predictors	Incidence Rate Ratios (95 % CI)
(Intercept)	2.99** (1.26 – 5.99)
Current health status [Good]	9.43*** (4.64 – 22.60)
Current health status [Moderate]	5.71*** (2.73 – 13.95)
Current health status [Poor]	0.57 (0.15 – 1.89)
Becoming healthier [Yes]	1.34 (0.93 – 1.94)
Observations	8
R^2	1.000
	* p<0.05 ** p<0.01 *** p<0.001

Fig. S23: Continuation of χ^2 test of independence results for significant dependence between questions in the self-reported questionnaire. See the legend of Fig. S22 for details.

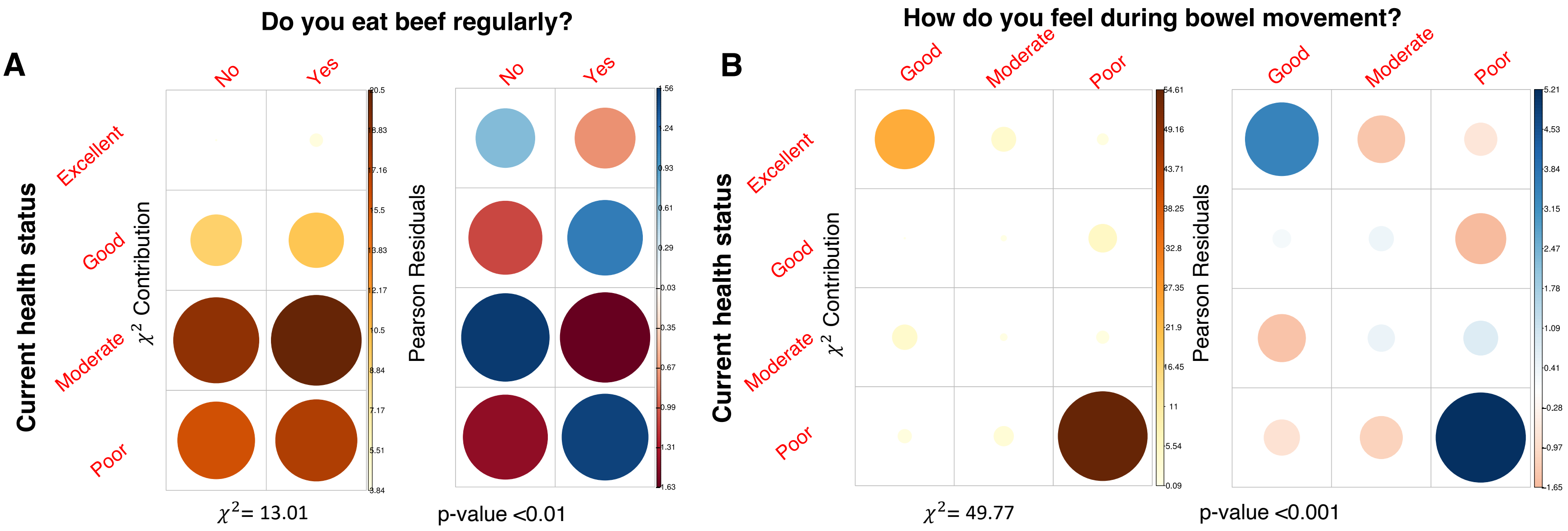

Predictors	Incidence Rate Ratios (95 % CI)
(Intercept)	3.65*** (1.54 – 7.23)
Current health status [Good]	9.43*** (4.64 – 22.60)
Current health status [Moderate]	5.71*** (2.73 – 13.95)
Current health status [Poor]	0.57 (0.15 – 1.89)
Beef consumption [Yes]	0.92 (0.64 – 1.32)
Observations	8
R^2	1.000
	* p<0.05 ** p<0.01 *** p<0.001

Predictors	Incidence Rate Ratios (95 % CI)
(Intercept)	1.20(0.47 – 2.58)
Current health status [Good]	9.43*** (4.64 – 22.60)
Current health status [Moderate]	5.71*** (2.73 – 13.95)
Current health status [Poor]	0.57 (0.15 – 1.89)
Bowl movement [Moderate]	4.45** (2.80-7.43)
Bowl movement [Poor]	0.40* (0.17 – 0.88)
Observations	12
R^2	1.000
	* p<0.05 ** p<0.01 *** p<0.001

Fig. S24: Continuation of χ^2 test of independence results for significant dependence between questions in the self-reported questionnaire. See the legend of Fig. S22 for details.

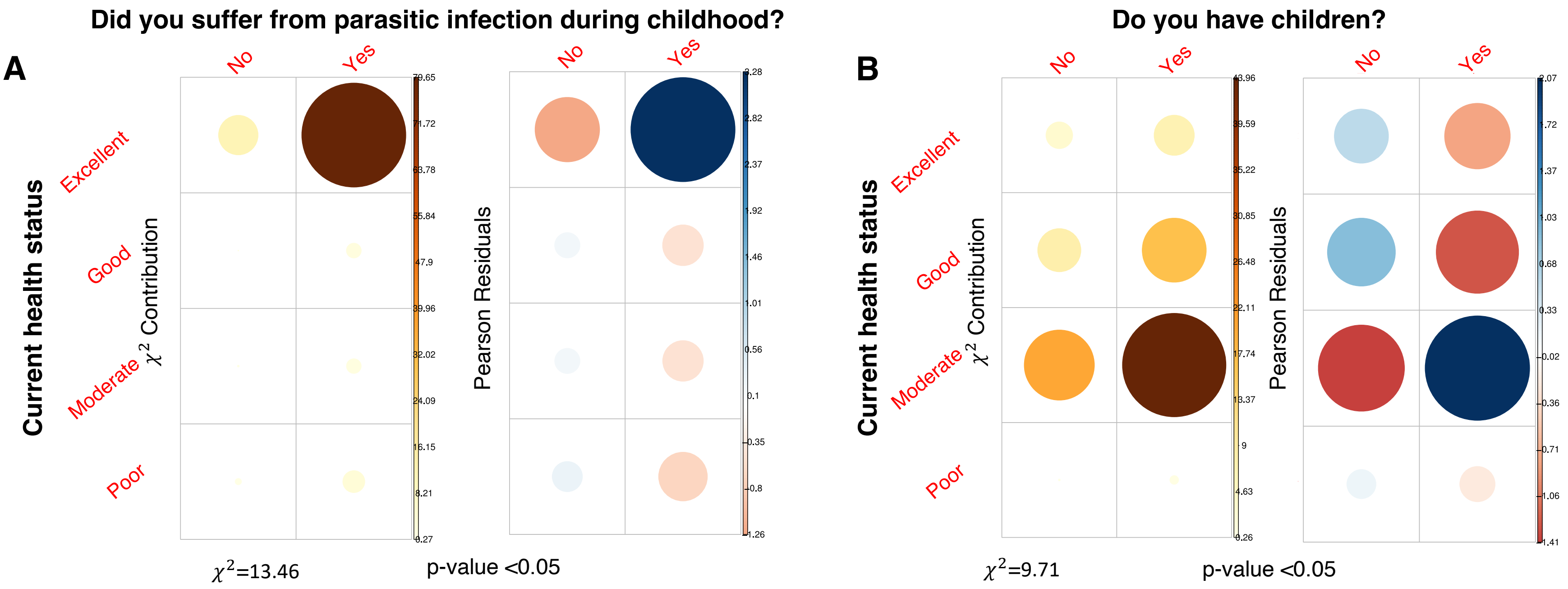

Predictors	Incidence Rate Ratios (95 % CI)
(Intercept)	6.10*** (2.62 – 11.85)
Current health status [Good]	9.43*** (4.64 – 22.60)
Current health status [Moderate]	5.71*** (2.73 – 13.95)
Current health status [Poor]	0.57 (0.15 – 1.89)
Childhood parasitic infecton [Yes]	0.15*** (0.08-0.24)
Observations	8
R^2	1.000
	* p<0.05 ** p<0.01 *** p<0.001

Predictors	Incidence Rate Ratios (95 % CI)
(Intercept)	4.79*** (2.04 – 9.37)
Current health status [Good]	9.43*** (4.64 – 22.60)
Current health status [Moderate]	5.71*** (2.73 – 13.95)
Current health status [Poor]	0.57 (0.15 – 1.89)
Children [Yes]	0.46*** (0.31-0.68)
Observations	8
R^2	1.000
	* p<0.05 ** p<0.01 *** p<0.001

Fig. S25: Continuation of χ^2 test of independence results for significant dependence between questions in the self-reported questionnaire. See the legend of Fig. S22 for details.

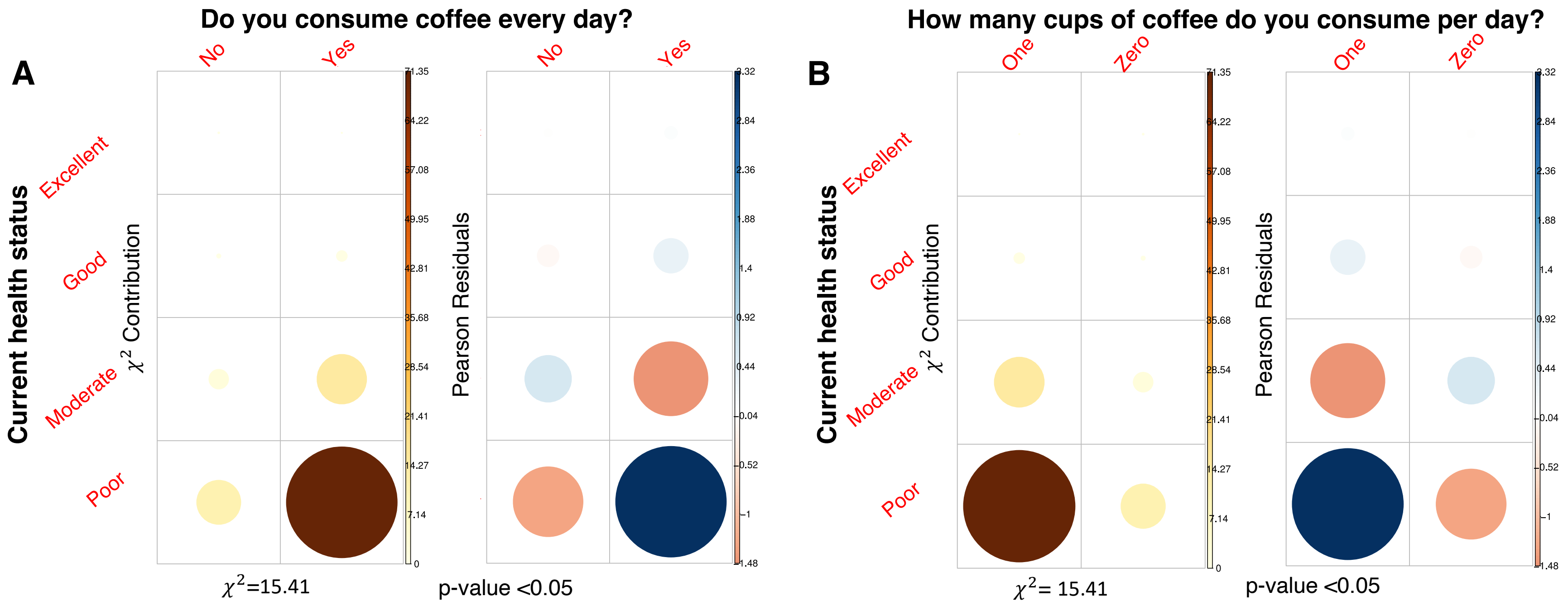

Predictors	Incidence Rate Ratios (95 % CI)
**(Intercept)**	6.04*** (2.59 – 11.73)
**Current health status [Good]**	9.43*** (4.64 – 22.60)
**Current health status [Moderate]**	5.71*** (2.73 – 13.95)
**Current health status [Poor]**	0.57 (0.15 – 1.89)
**Coffee consumption [Yes]**	0.16*** (0.09-0.26)
**Observations**	8
**R^2**	1.000
	*** p<0.05 ** p<0.01 *** p<0.001**

Predictors	Incidence Rate Ratios (95 % CI)
**(Intercept)**	0.96 (0.37 – 2.13)
**Current health status [Good]**	9.43*** (4.64 – 22.60)
**Current health status [Moderate]**	5.71*** (2.73 – 13.95)
**Current health status [Poor]**	0.57 (0.15 – 1.89)
**Coffee cups per day [Zero]**	6.31*** (3.84-11.11)
**Observations**	8
**R^2**	1.000
	*** p<0.05 ** p<0.01 *** p<0.001**

Fig. S26: Continuation of χ^2 test of independence results for significant dependence between questions in the self-reported questionnaire. See the legend of Fig. S22 for details.

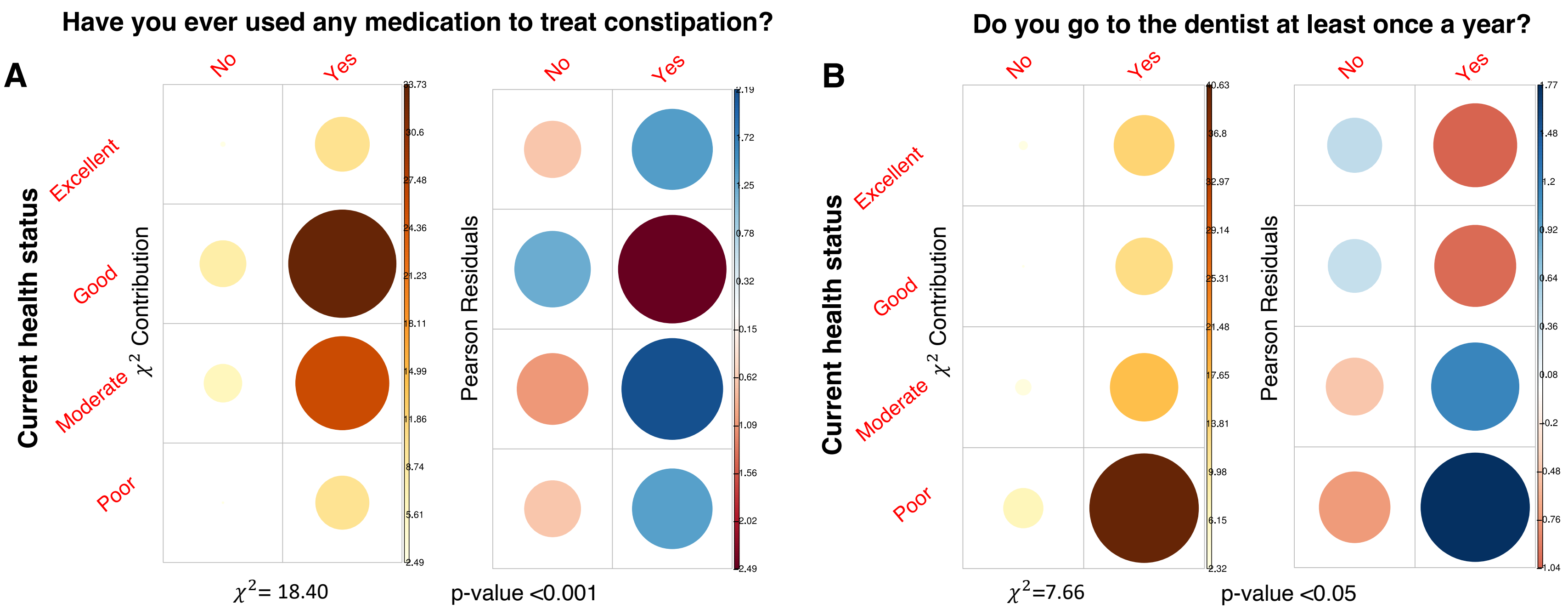

Predictors	Incidence Rate Ratios (95 % CI)
(Intercept)	5.62*** (2.41 – 10.95)
Current health status [Good]	9.43*** (4.64 – 22.60)
Current health status [Moderate]	5.71*** (2.73 – 13.95)
Current health status [Poor]	0.57 (0.15 – 1.89)
Constipation treatment [Yes]	0.24*** (0.15-0.38)
Observations	8
R^2	1.000
	* p<0.05 ** p<0.01 *** p<0.001

Predictors	Incidence Rate Ratios (95 % CI)
(Intercept)	5.92*** (2.54 – 11.51)
Current health status [Good]	9.43*** (4.64 – 22.60)
Current health status [Moderate]	5.71*** (2.73 – 13.95)
Current health status [Poor]	0.57 (0.15 – 1.89)
Dentist visit [Yes]	0.18*** (0.11-0.29)
Observations	8
R^2	1.000
	* p<0.05 ** p<0.01 *** p<0.001

Fig. S27: Continuation of χ^2 test of independence results for significant dependence between questions in the self-reported questionnaire. See the legend of Fig. S22 for details.

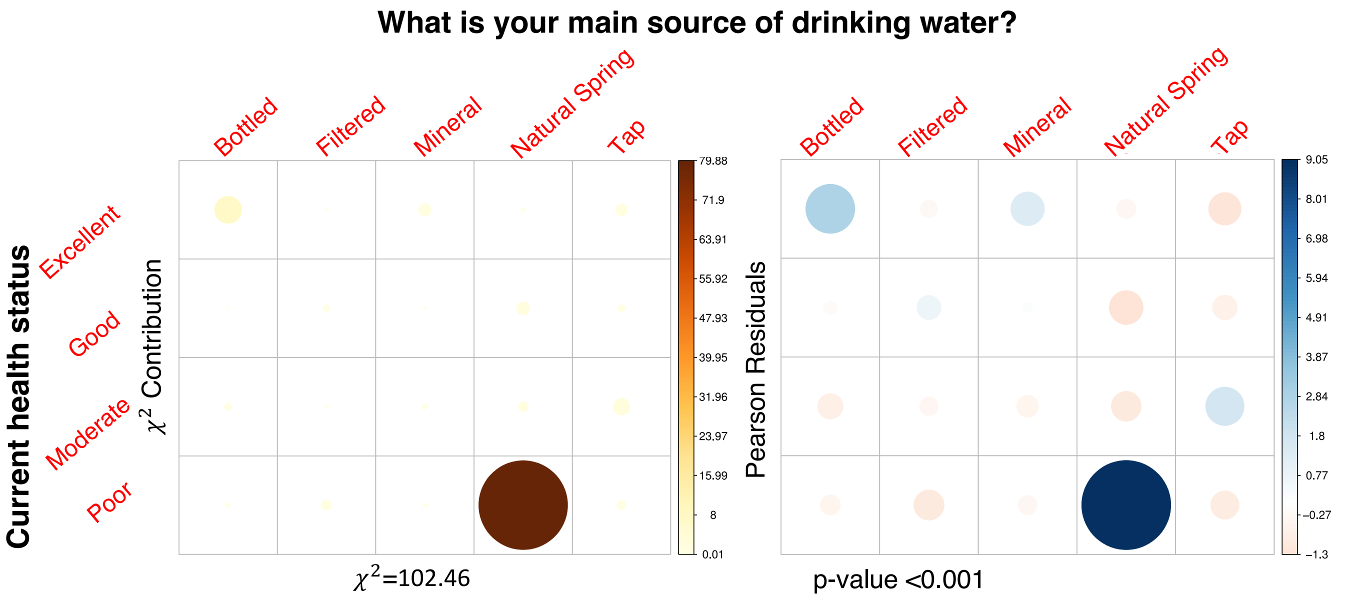

Predictors	Incidence Rate Ratios (95 % CI)
(Intercept)	0.36 (0.11 – 0.96)
Current health status [Good]	9.43*** (4.64 – 22.60)
Current health status [Moderate]	5.71*** (2.73 – 13.95)
Current health status [Poor]	0.57 (0.15 – 1.89)
Drinking water source [Filtered]	13.17*** (6.25-33.88)
Drinking water source [Mineral]	0.83 (0.24 – 2.77)
Drinking water source [Other]	0.50 (0.11 – 1.90)
Drinking water source [Tap]	4.00** (1.74 – 10.80)
Observations	20
R^2	1.000
	* p<0.05 ** p<0.01 *** p<0.001

Fig. S28: Continuation of χ^2 test of independence results for significant dependence between questions in the self-reported questionnaire. See the legend of Fig. S22 for details.

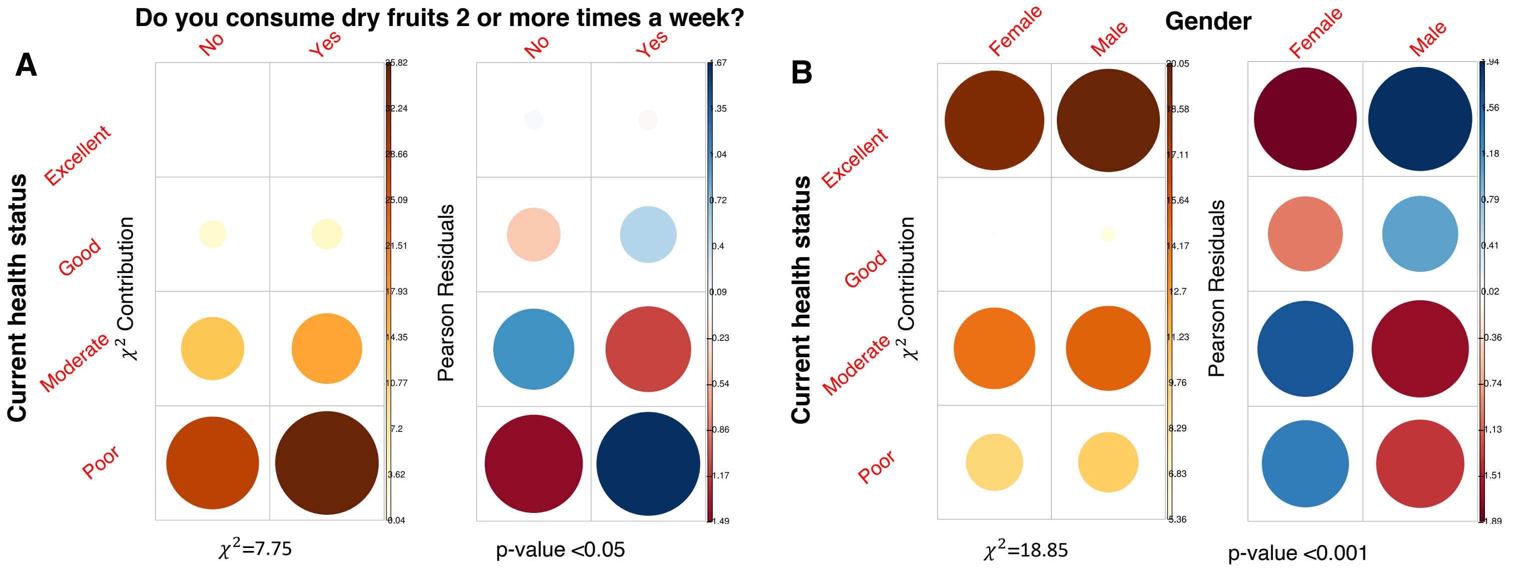

Predictors	Incidence Rate Ratios (95 % CI)
(Intercept)	3.89*** (1.65– 7.68)
Current health status [Good]	9.43*** (4.64 – 22.60)
Current health status [Moderate]	5.71*** (2.73 – 13.95)
Current health status [Poor]	0.57 (0.15 – 1.89)
Dryfruits consumption [Yes]	0.80 (0.55-1.15)
Observations	8
R^2	1.000
	* p<0.05 ** p<0.01 *** p<0.001

Predictors	Incidence Rate Ratios (95 % CI)
(Intercept)	3.59** (1.52– 7.11)
Current health status [Good]	9.43*** (4.64 – 22.60)
Current health status [Moderate]	5.71*** (2.73 – 13.95)
Current health status [Poor]	0.57 (0.15 – 1.89)
Gender [Male]	0.95 (0.66-1.37)
Observations	8
R^2	1.000
	* p<0.05 ** p<0.01 *** p<0.001

Fig. S29: Continuation of χ^2 test of independence results for significant dependence between questions in the self-reported questionnaire. See the legend of Fig. S22 for details.

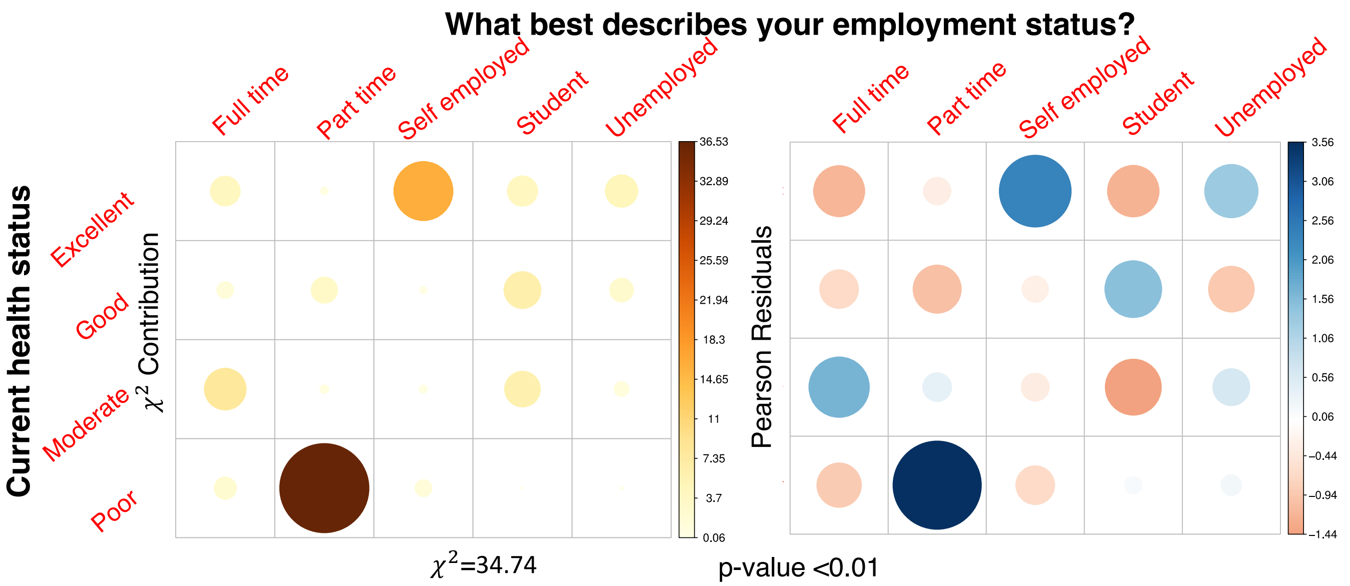

Predictors	Incidence Rate Ratios (95 % CI)
(Intercept)	1.44 (0.58 – 3.04)
Current health status [Good]	9.43*** (4.64 – 22.60)
Current health status [Moderate]	5.71*** (2.73 – 13.95)
Current health status [Poor]	0.57 (0.15 – 1.89)
Employment status [Part time]	0.08*** (0.01-0.28)
Employment status [Self employed]	0.58 (0.29 – 1.11)
Employment status [Student]	2.21** (1.38 – 3.64)
Employment status [Unemployed]	1.00 (0.57– 1.77)
Observations	20
R^2	1.000
	* p<0.05 ** p<0.01 *** p<0.001

Fig. S30: Continuation of χ^2 test of independence results for significant dependence between questions in the self-reported questionnaire. See the legend of Fig. S22 for details.

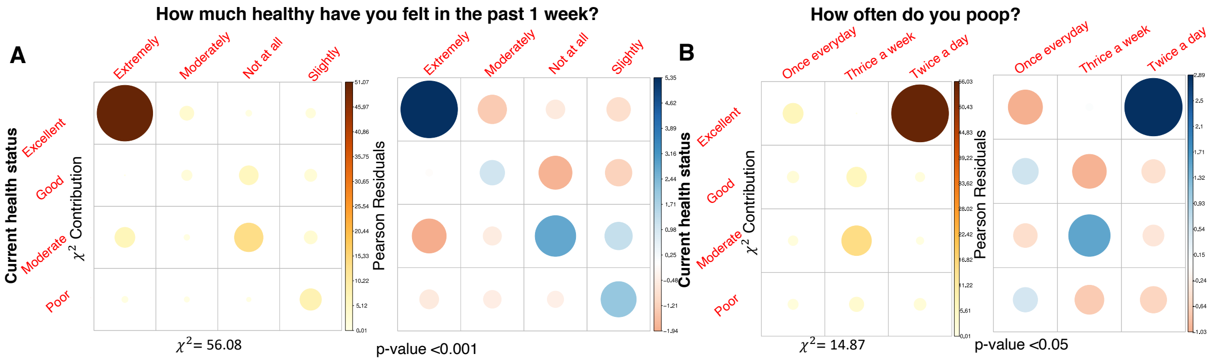

Predictors	Incidence Rate Ratios (95 % CI)
(Intercept)	0.66 (0.24 – 1.55)
Current health status [Good]	9.43*** (4.64 – 22.60)
Current health status [Moderate]	5.71*** (2.73 – 13.95)
Current health status [Poor]	0.57 (0.15 – 1.89)
Felt healthy past week [Moderately]	7.73*** (4.31-15.34)
Felt healthy past week [Not at all]	0.55 (0.19 – 1.43)
Felt healthy past week [Slightly]	1.36 (0.63 – 3.05)
Observations	16
R^2	1.000

* p<0.05 ** p<0.01 *** p<0.001

Predictors	Incidence Rate Ratios (95 % CI)
(Intercept)	5.38*** (2.30 – 10.50)
Current health status [Good]	9.43*** (4.64 – 22.60)
Current health status [Moderate]	5.71*** (2.73 – 13.95)
Current health status [Poor]	0.57 (0.15 – 1.89)
Poop frequency [Thrice a week]	0.18*** (0.10-0.29)
Poop frequency [Twice a day]	0.12*** (0.06-0.22)
Observations	12
R^2	1.000

* p<0.05 ** p<0.01 *** p<0.001

Fig. S31: Continuation of χ^2 test of independence results for significant dependence between questions in the self-reported questionnaire. See the legend of Fig. S22 for details.

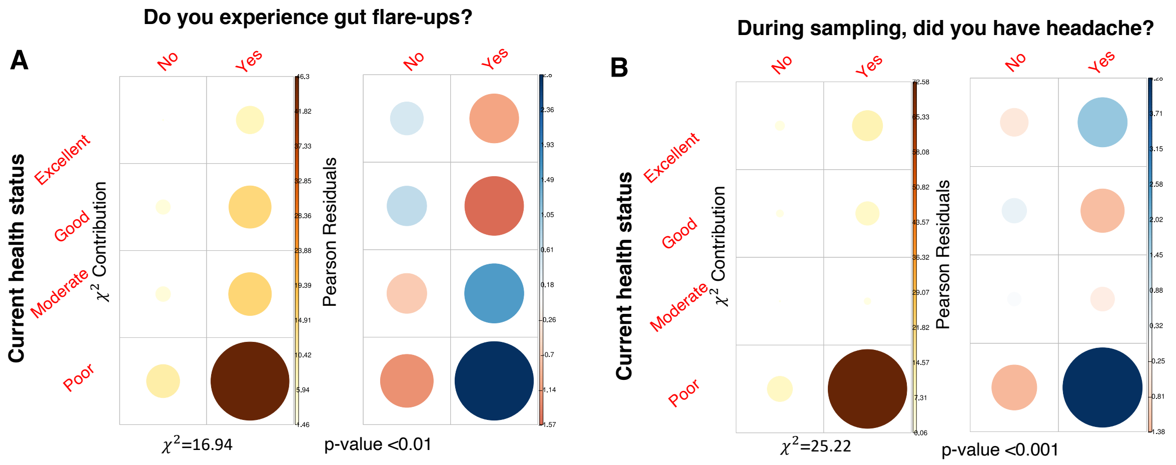

Predictors	Incidence Rate Ratios (95 % CI)
(Intercept)	5.80*** (2.48– 11.28)
Current health status [Good]	9.43*** (4.64 – 22.60)
Current health status [Moderate]	5.71*** (2.73 – 13.95)
Current health status [Poor]	0.57 (0.15 – 1.89)
Gut flare ups [Yes]	0.21*** (0.12-0.33)
Observations	8
R^2	1.000
	* p<0.05 ** p<0.01 *** p<0.001

Predictors	Incidence Rate Ratios (95 % CI)
(Intercept)	6.34*** (2.72– 12.30)
Current health status [Good]	9.43*** (4.64 – 22.60)
Current health status [Moderate]	5.71*** (2.73 – 13.95)
Current health status [Poor]	0.57 (0.15 – 1.89)
Headaches DS [Yes]	0.10*** (0.05-0.18)
Observations	8
R^2	1.000
	* p<0.05 ** p<0.01 *** p<0.001

Fig. S32: Continuation of χ^2 test of independence results for significant dependence between questions in the self-reported questionnaire. See the legend of Fig. S22 for details.

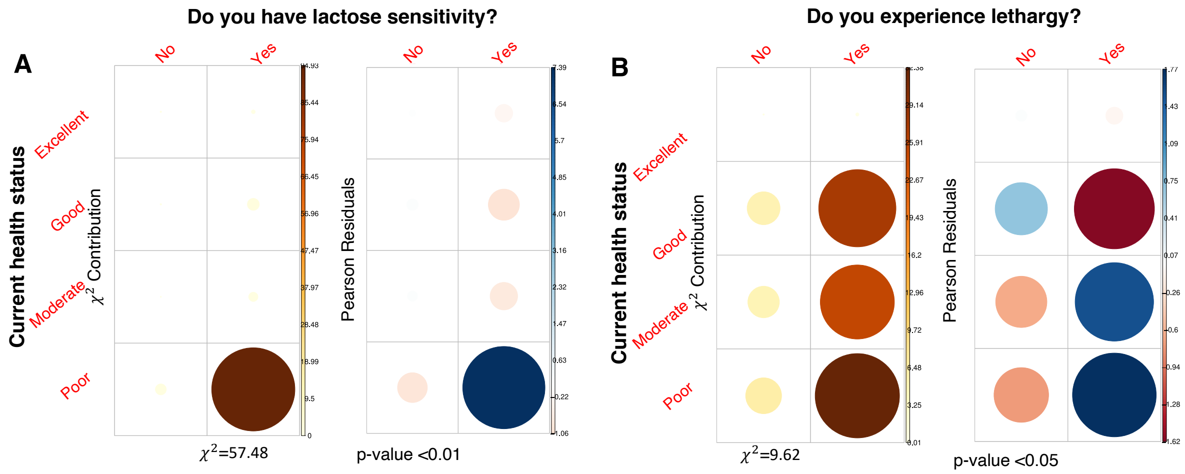

Predictors	Incidence Rate Ratios (95 % CI)
(Intercept)	6.88*** (2.96– 13.31)
Current health status [Good]	9.43*** (4.64 – 22.60)
Current health status [Moderate]	5.71*** (2.73 – 13.95)
Current health status [Poor]	0.57 (0.15 – 1.89)
Lactose sensitivity [Yes]	0.02*** (0.00-0.05)
Observations	8
R^2	1.000
	* p<0.05 ** p<0.01 *** p<0.001

Predictors	Incidence Rate Ratios (95 % CI)
(Intercept)	5.92*** (2.54– 11.51)
Current health status [Good]	9.43*** (4.64 – 22.60)
Current health status [Moderate]	5.71*** (2.73 – 13.95)
Current health status [Poor]	0.57 (0.15 – 1.89)
Lethargy [Yes]	0.18*** (0.11-0.29)
Observations	8
R^2	1.000
	* p<0.05 ** p<0.01 *** p<0.001

Fig. S33: Continuation of χ^2 test of independence results for significant dependence between questions in the self-reported questionnaire. See the legend of Fig. S22 for details.

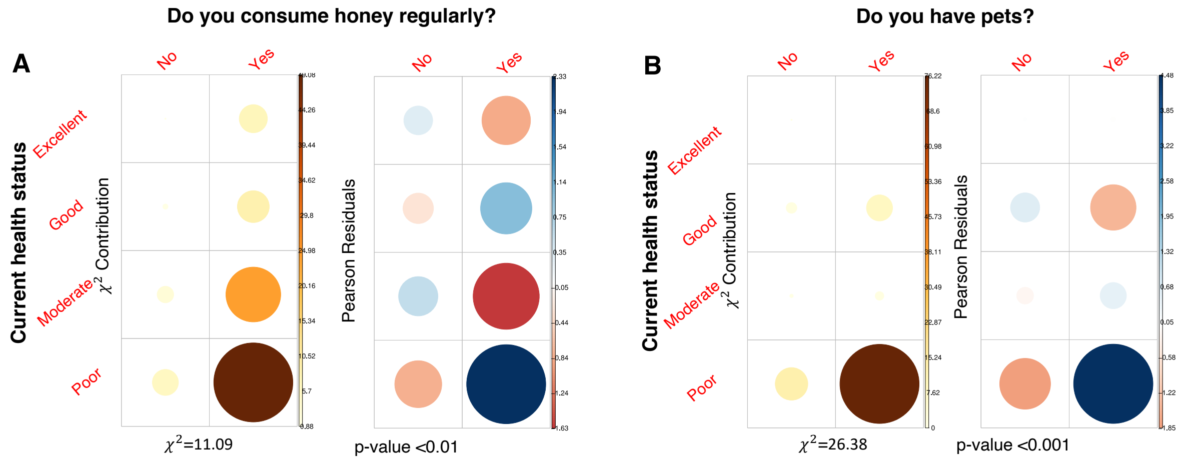

Predictors	Incidence Rate Ratios (95 % CI)
(Intercept)	6.22*** (2.67– 12.07)
Current health status [Good]	9.43*** (4.64 – 22.60)
Current health status [Moderate]	5.71*** (2.73 – 13.95)
Current health status [Poor]	0.57 (0.15 – 1.89)
Honey consumption [Yes]	0.13*** (0.07-0.21)
Observations	8
R^2	1.000
	* p<0.05 ** p<0.01 *** p<0.001

Predictors	Incidence Rate Ratios (95 % CI)
(Intercept)	5.98*** (2.56– 11.62)
Current health status [Good]	9.43*** (4.64 – 22.60)
Current health status [Moderate]	5.71*** (2.73 – 13.95)
Current health status [Poor]	0.57 (0.15 – 1.89)
Household animals [Yes]	0.17*** (0.10-0.28)
Observations	8
R^2	1.000
	* p<0.05 ** p<0.01 *** p<0.001

Fig. S34: Continuation of χ^2 test of independence results for significant dependence between questions in the self-reported questionnaire. See the legend of Fig. S22 for details.

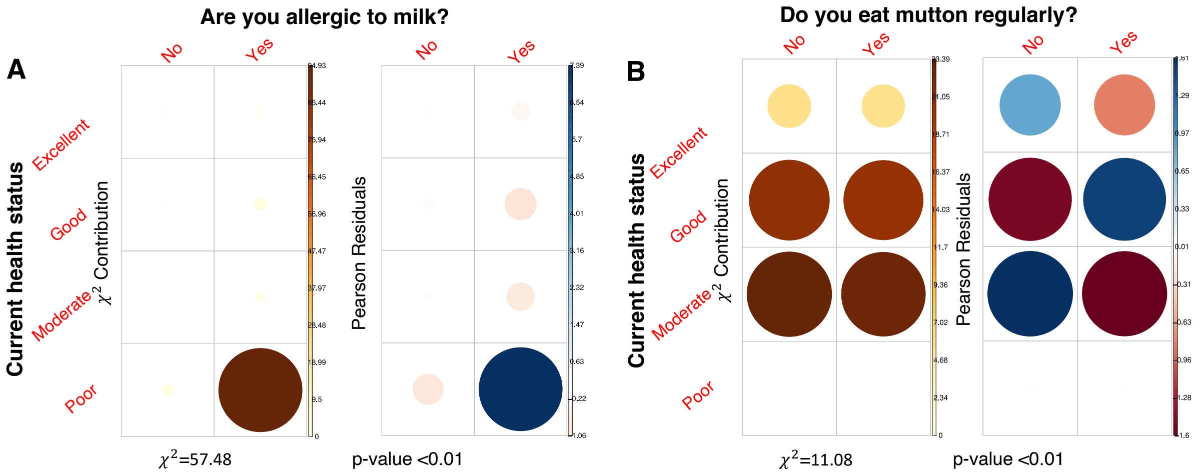

Predictors	Incidence Rate Ratios (95 % CI)
(Intercept)	6.88*** (2.96– 13.31)
Current health status [Good]	9.43*** (4.64 – 22.60)
Current health status [Moderate]	5.71*** (2.73 – 13.95)
Current health status [Poor]	0.57 (0.15 – 1.89)
Milk allergy [Yes]	0.02*** (0.00-0.05)
Observations	8
R^2	1.000
	* p<0.05 ** p<0.01 *** p<0.001

Predictors	Incidence Rate Ratios (95 % CI)
(Intercept)	3.47** (1.46– 6.89)
Current health status [Good]	9.43*** (4.64 – 22.60)
Current health status [Moderate]	5.71*** (2.73 – 13.95)
Current health status [Poor]	0.57 (0.15 – 1.89)
Mutton consumption [Yes]	1.02 (0.71-1.46)
Observations	8
R^2	1.000
	* p<0.05 ** p<0.01 *** p<0.001

Fig. S35: Continuation of χ^2 test of independence results for significant dependence between questions in the self-reported questionnaire. See the legend of Fig. S22 for details.

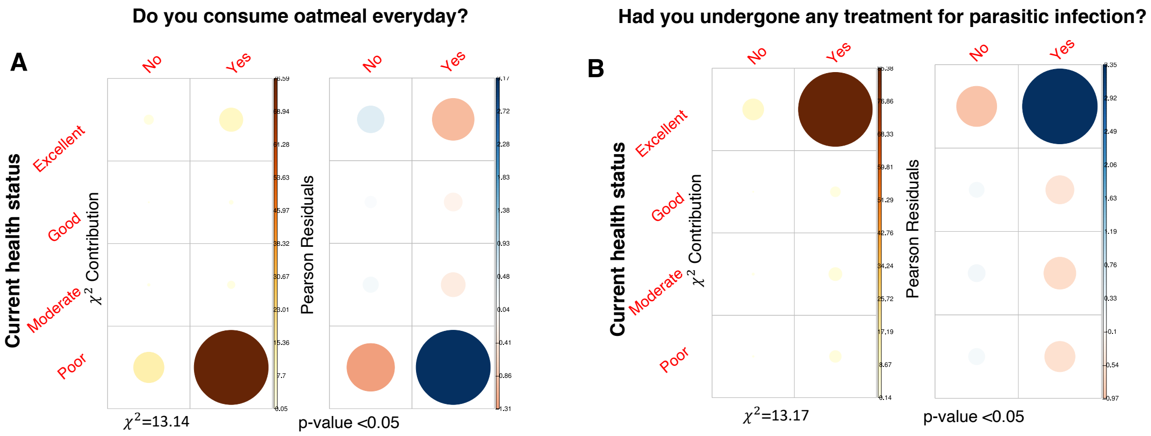

Predictors	Incidence Rate Ratios (95 % CI)
(Intercept)	5.98*** (2.56– 11.62)
Current health status [Good]	9.43*** (4.64 – 22.60)
Current health status [Moderate]	5.71*** (2.73 – 13.95)
Current health status [Poor]	0.57 (0.15 – 1.89)
Oatmeal consumption [Yes]	0.17*** (0.10-0.28)
Observations	8
R^2	1.000
	* p<0.05 ** p<0.01 *** p<0.001

Predictors	Incidence Rate Ratios (95 % CI)
(Intercept)	6.46*** (2.77– 12.52)
Current health status [Good]	9.43*** (4.64 – 22.60)
Current health status [Moderate]	5.71*** (2.73 – 13.95)
Current health status [Poor]	0.57 (0.15 – 1.89)
Parasitic infection treatment [Yes]	0.08*** (0.04-0.16)
Observations	8
R^2	1.000
	* p<0.05 ** p<0.01 *** p<0.001

Fig. S36: Continuation of χ^2 test of independence results for significant dependence between questions in the self-reported questionnaire. See the legend of Fig. S22 for details.

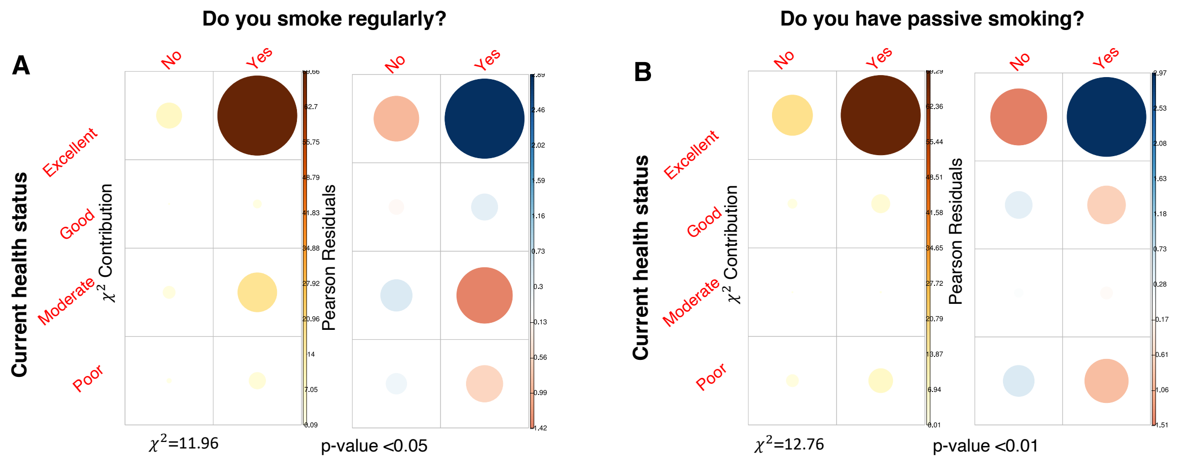

Predictors	Incidence Rate Ratios (95 % CI)
(Intercept)	5.98*** (2.38– 10.83)
Current health status [Good]	9.43*** (4.64 – 22.60)
Current health status [Moderate]	5.71*** (2.73 – 13.95)
Current health status [Poor]	0.57 (0.15 – 1.89)
Smoking [Yes]	0.26*** (0.16-0.40)
Observations	8
R^2	1.000
	* p<0.05 ** p<0.01 *** p<0.001

Predictors	Incidence Rate Ratios (95 % CI)
(Intercept)	6.34*** (2.72– 12.30)
Current health status [Good]	9.43*** (4.64 – 22.60)
Current health status [Moderate]	5.71*** (2.73 – 13.95)
Current health status [Poor]	0.57 (0.15 – 1.89)
Passive smoking [Yes]	0.10*** (0.05-0.18)
Observations	8
R^2	1.000
	* p<0.05 ** p<0.01 *** p<0.001

Fig. S37: Continuation of χ^2 test of independence results for significant dependence between questions in the self-reported questionnaire. See the legend of Fig. S22 for details.

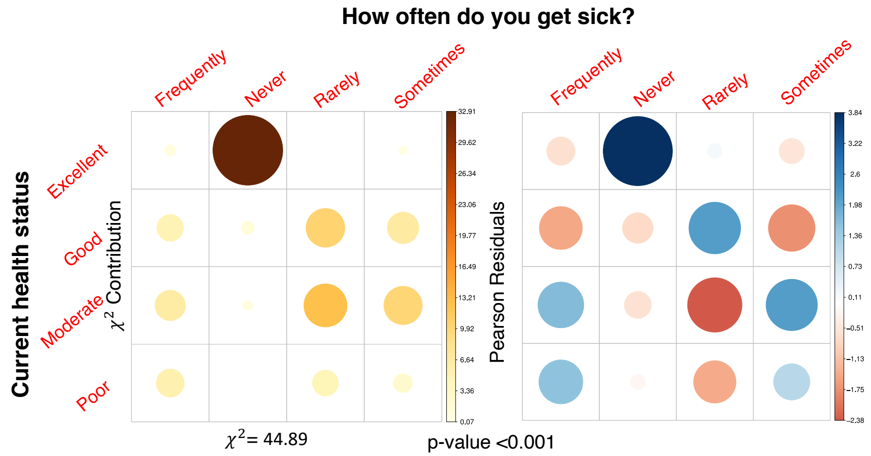

Predictors	Incidence Rate Ratios (95 % CI)
(Intercept)	0.42 (0.13 – 1.08)
Current health status [Good]	9.43*** (4.64 – 22.60)
Current health status [Moderate]	5.71*** (2.73 – 13.95)
Current health status [Poor]	0.57 (0.15 – 1.89)
Sickness frequency [Never]	0.14 (0.01-0.80)
Sickness frequency [Rarely]	8.71*** (4.28 – 20.93)
Sickness frequency [Sometimes]	6.86*** (2.32 – 16.61)
Observations	16
R^2	1.000
	* p<0.05 ** p<0.01 *** p<0.001

Fig. S38: Continuation of χ^2 test of independence results for significant dependence between questions in the self-reported questionnaire. See the legend of Fig. S22 for detail.

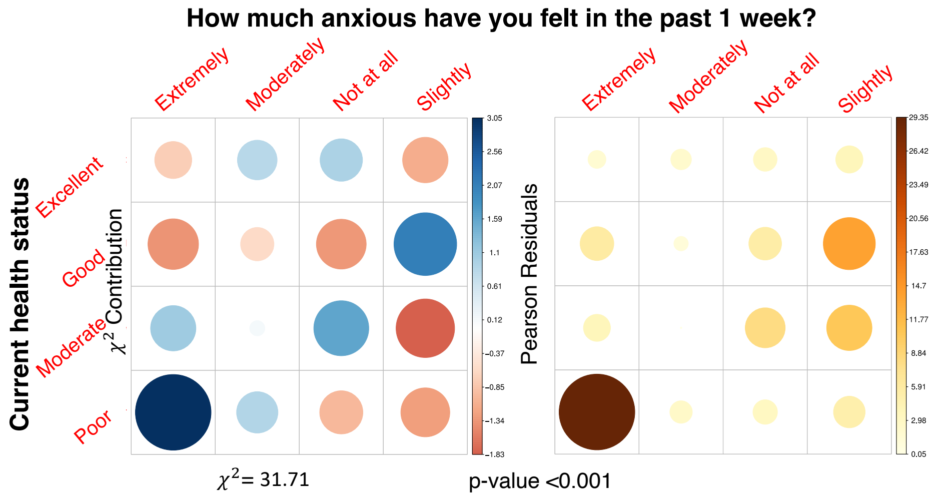

Predictors	Incidence Rate Ratios (95 % CI)
(Intercept)	0.54 (0.19 – 1.31)
Current health status [Good]	9.43*** (4.64 – 22.60)
Current health status [Moderate]	5.71*** (2.73 – 13.95)
Current health status [Poor]	0.57 (0.15 – 1.89)
Recently anxious [Moderately]	3.44** (1.71-7.69)
Recently anxious [Not at all]	3.22** (1.59 – 7.22)
Recently anxious [Slightly]	5.33*** (2.75 – 11.63)
Observations	16
R^2	1.000
	* p<0.05 ** p<0.01 *** p<0.001

Fig. S39: Continuation of χ^2 test of independence results for significant dependence between questions in the self-reported questionnaire. See the legend of Fig. S22 for detail.

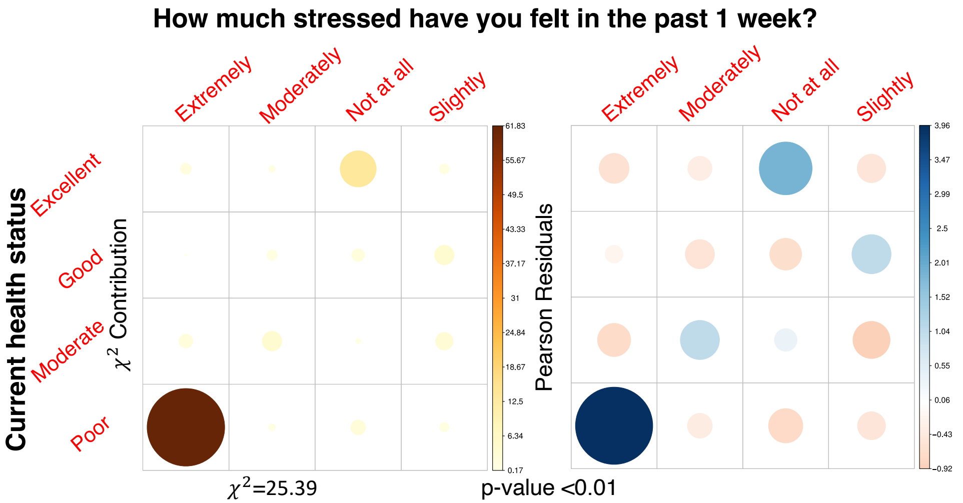

Predictors	Incidence Rate Ratios (95 % CI)
(Intercept)	0.36 (0.11 – 0.96)
Current health status [Good]	9.43*** (4.64 – 22.60)
Current health status [Moderate]	5.71*** (2.73 – 13.95)
Current health status [Poor]	0.57 (0.15 – 1.89)
Recently stressed [Moderately]	7.33*** (3.38-19.19)
Recently stressed [Not at all]	3.00* (0.19 – 1.43)
Recently stressed [Slightly]	8.17*** (3.79 – 21.29)
Observations	16
R^2	1.000
	* p<0.05 ** p<0.01 *** p<0.001

Fig. S40: Continuation of χ^2 test of independence results for significant dependence between questions in the self-reported questionnaire. See the legend of Fig. S22 for detail.

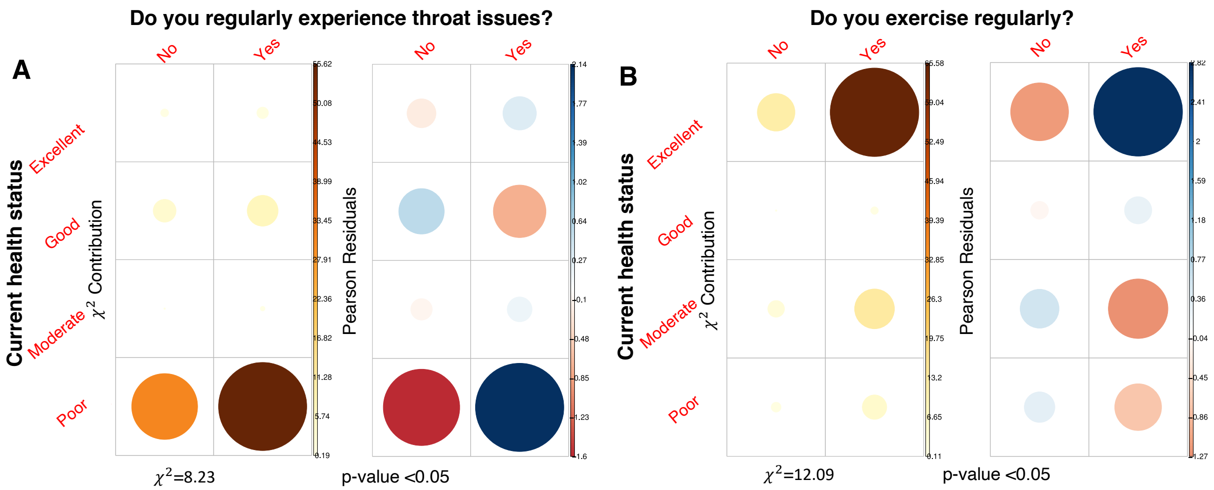

Predictors	Incidence Rate Ratios (95 % CI)
(Intercept)	4.49*** (1.91– 8.81)
Current health status [Good]	9.43*** (4.64 – 22.60)
Current health status [Moderate]	5.71*** (2.73 – 13.95)
Current health status [Poor]	0.57 (0.15 – 1.89)
Regular throat issue [Yes]	0.56*** (0.38-0.81)
Observations	8
R^2	1.000
	* p<0.05 ** p<0.01 *** p<0.001

Predictors	Incidence Rate Ratios (95 % CI)
(Intercept)	5.92*** (2.54– 11.51)
Current health status [Good]	9.43*** (4.64 – 22.60)
Current health status [Moderate]	5.71*** (2.73 – 13.95)
Current health status [Poor]	0.57 (0.15 – 1.89)
Regular excercise [Yes]	0.18*** (0.11-0.29)
Observations	8
R^2	1.000
	* p<0.05 ** p<0.01 *** p<0.001

Fig. S41: Continuation of χ^2 test of independence results for significant dependence between questions in the self-reported questionnaire. See the legend of Fig. S22 for detail.

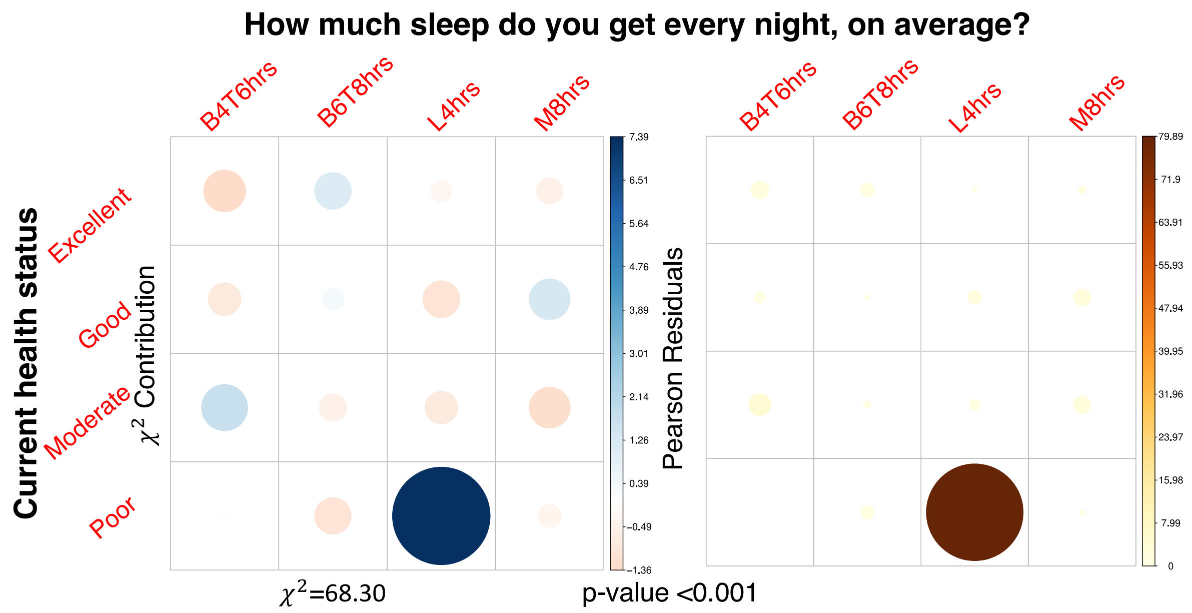

Predictors	Incidence Rate Ratios (95 % CI)
(Intercept)	1.85 (0.76 – 3.84)
Current health status [Good]	9.43*** (4.64 – 22.60)
Current health status [Moderate]	5.71*** (2.73 – 13.95)
Current health status [Poor]	0.57 (0.15 – 1.89)
Sleep time [B6T8hrs]	2.55*** (1.70-3.92)
Sleep time [L4hrs]	0.06*** (0.01 – 0.21)
Sleep time [M8hrs]	0.16*** (0.06– 0.38)
Observations	16
R^2	1.000
	* p<0.05 ** p<0.01 *** p<0.001

Fig. S42: Continuation of χ^2 test of independence results for significant dependence between questions in the self-reported questionnaire. See the legend of Fig. S22 for detail.

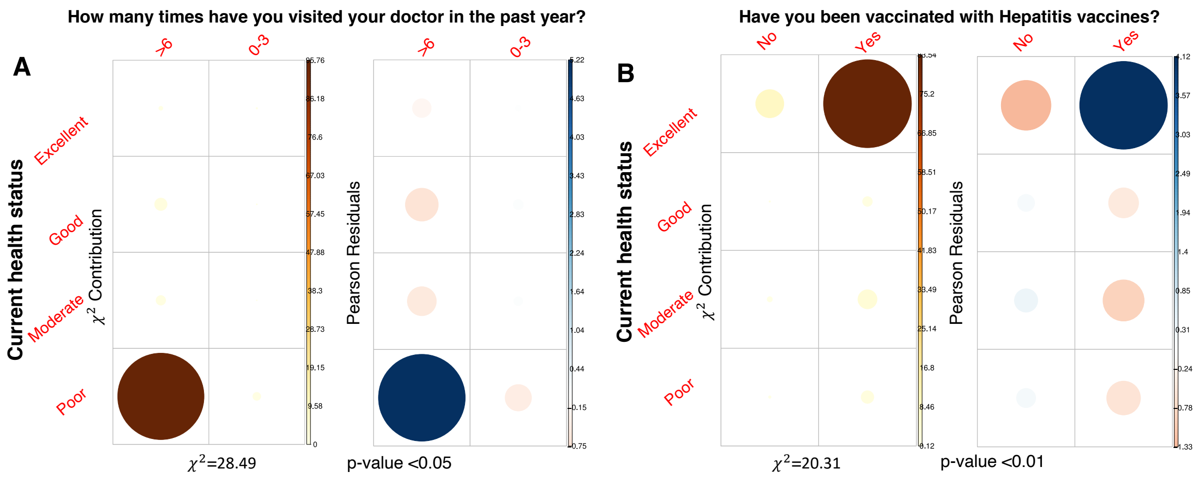

Predictors	Incidence Rate Ratios (95 % CI)
(Intercept)	0.06** (0.00– 0.32)
Current health status [Good]	9.43*** (4.64 – 22.60)
Current health status [Moderate]	5.71*** (2.73 – 13.95)
Current health status [Poor]	0.57 (0.15 – 1.89)
Visited doctor frequency [0-3]	116.0*** (25.98-2042.33)
Observations	8
R^2	1.000

* p<0.05 ** p<0.01 *** p<0.001

Predictors	Incidence Rate Ratios (95 % CI)
(Intercept)	6.34*** (2.72– 12.30)
Current health status [Good]	9.43*** (4.64 – 22.60)
Current health status [Moderate]	5.71*** (2.73 – 13.95)
Current health status [Poor]	0.57 (0.15 – 1.89)
Vaccinated hepatitis [Yes]	0.10*** (0.05-0.18)
Observations	8
R^2	1.000

* p<0.05 ** p<0.01 *** p<0.001

Fig. S43: Continuation of χ^2 test of independence results for significant dependence between questions in the self-reported questionnaire. See the legend of Fig. S22 for detail.

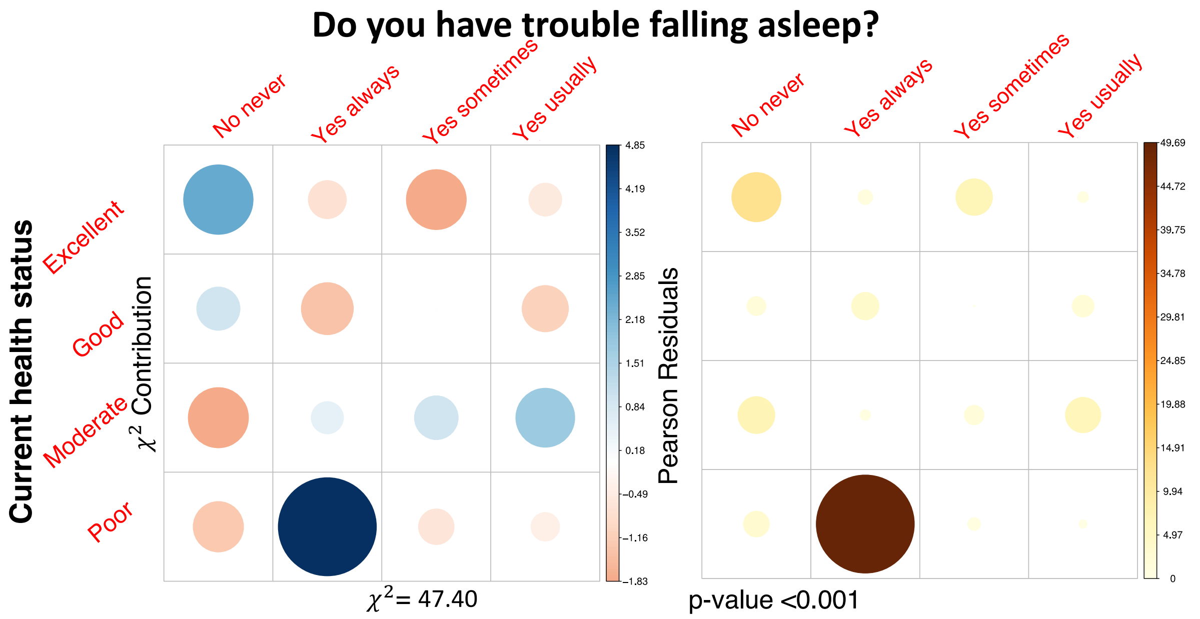

Predictors	Incidence Rate Ratios (95 % CI)
(Intercept)	2.87** (1.20 – 5.76)
Current health status [Good]	9.43*** (4.64 – 22.60)
Current health status [Moderate]	5.71*** (2.73 – 13.95)
Current health status [Poor]	0.57 (0.15 – 1.89)
Trouble falling asleep [Yes always]	0.19*** (0.09-0.36)
Trouble falling asleep [Yes sometimes]	1.15 (0.78 – 1.69)
Trouble falling asleep [Yes usually]	0.10*** (0.04 – 0.24)
Observations	16
R^2	1.000
	* p<0.05 ** p<0.01 *** p<0.001

Fig. S44: Continuation of χ^2 test of independence results for significant dependence between questions in the self-reported questionnaire. See the legend of Fig. S22 for detail.

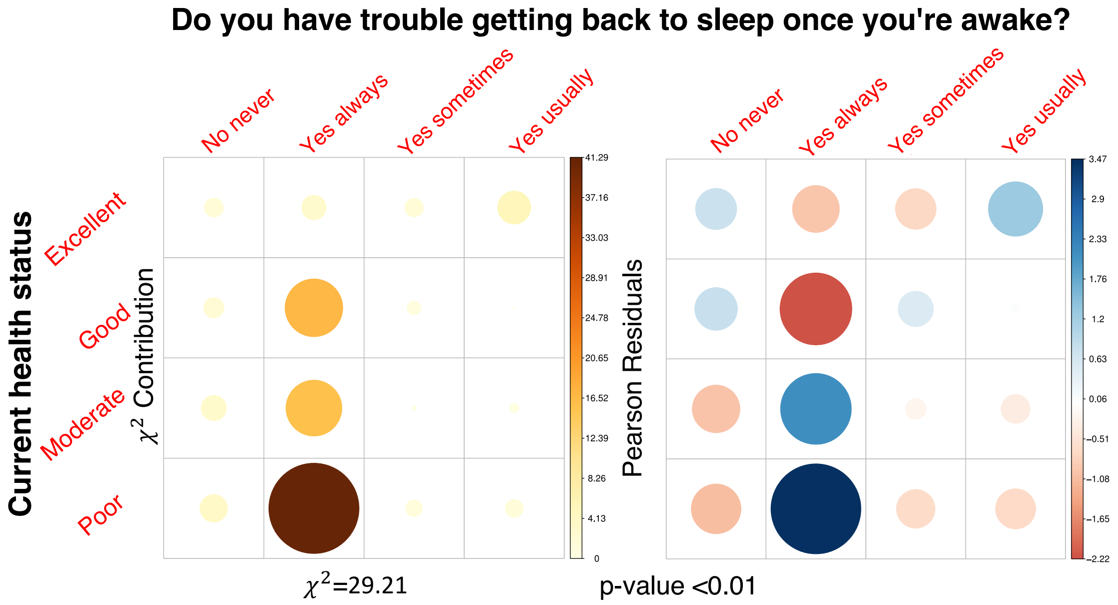

Predictors	Incidence Rate Ratios (95 % CI)
(Intercept)	1.97 (0.81 – 4.06)
Current health status [Good]	9.43*** (4.64 – 22.60)
Current health status [Moderate]	5.71*** (2.73 – 13.95)
Current health status [Poor]	0.57 (0.15 – 1.89)
Trouble back to sleep once awake [Yes always]	0.45* (0.24-0.82)
Trouble back to sleep once awake [Yes sometimes]	1.67* (1.09 – 2.59)
Trouble back to sleep once awake [Yes usually]	0.42** (0.22 – 0.78)
Observations	16
R^2	1.000
	* p<0.05 ** p<0.01 *** p<0.001

Fig. S45: Continuation of χ^2 test of independence results for significant dependence between questions in the self-reported questionnaire. See the legend of Fig. S22 for detail.

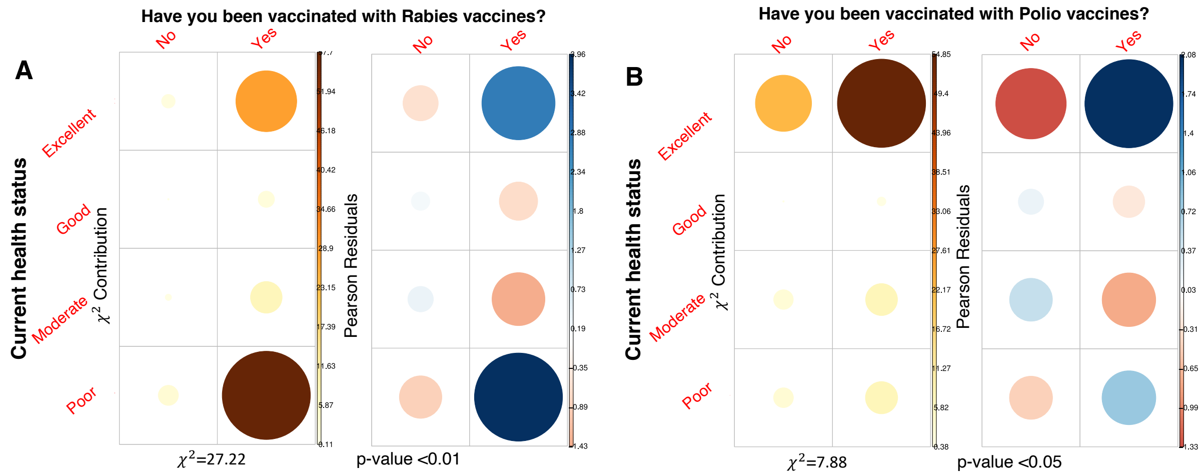

Predictors	Incidence Rate Ratios (95 % CI)
(Intercept)	6.64*** (2.85– 12.86)
Current health status [Good]	9.43*** (4.64 – 22.60)
Current health status [Moderate]	5.71*** (2.73 – 13.95)
Current health status [Poor]	0.57 (0.15 – 1.89)
Vaccinated Rabies [Yes]	0.05*** (0.02-0.11)
Observations	8
R^2	1.000
	* p<0.05 ** p<0.01 *** p<0.001

Predictors	Incidence Rate Ratios (95 % CI)
(Intercept)	4.97*** (2.12– 9.71)
Current health status [Good]	9.43*** (4.64 – 22.60)
Current health status [Moderate]	5.71*** (2.73 – 13.95)
Current health status [Poor]	0.57 (0.15 – 1.89)
Vaccinated Polio [Yes]	0.41*** (0.27-0.60)
Observations	8
R^2	1.000
	* p<0.05 ** p<0.01 *** p<0.001

Fig. S46: Continuation of χ^2 test of independence results for significant dependence between questions in the self-reported questionnaire. See the legend of Fig. S22 for detail.

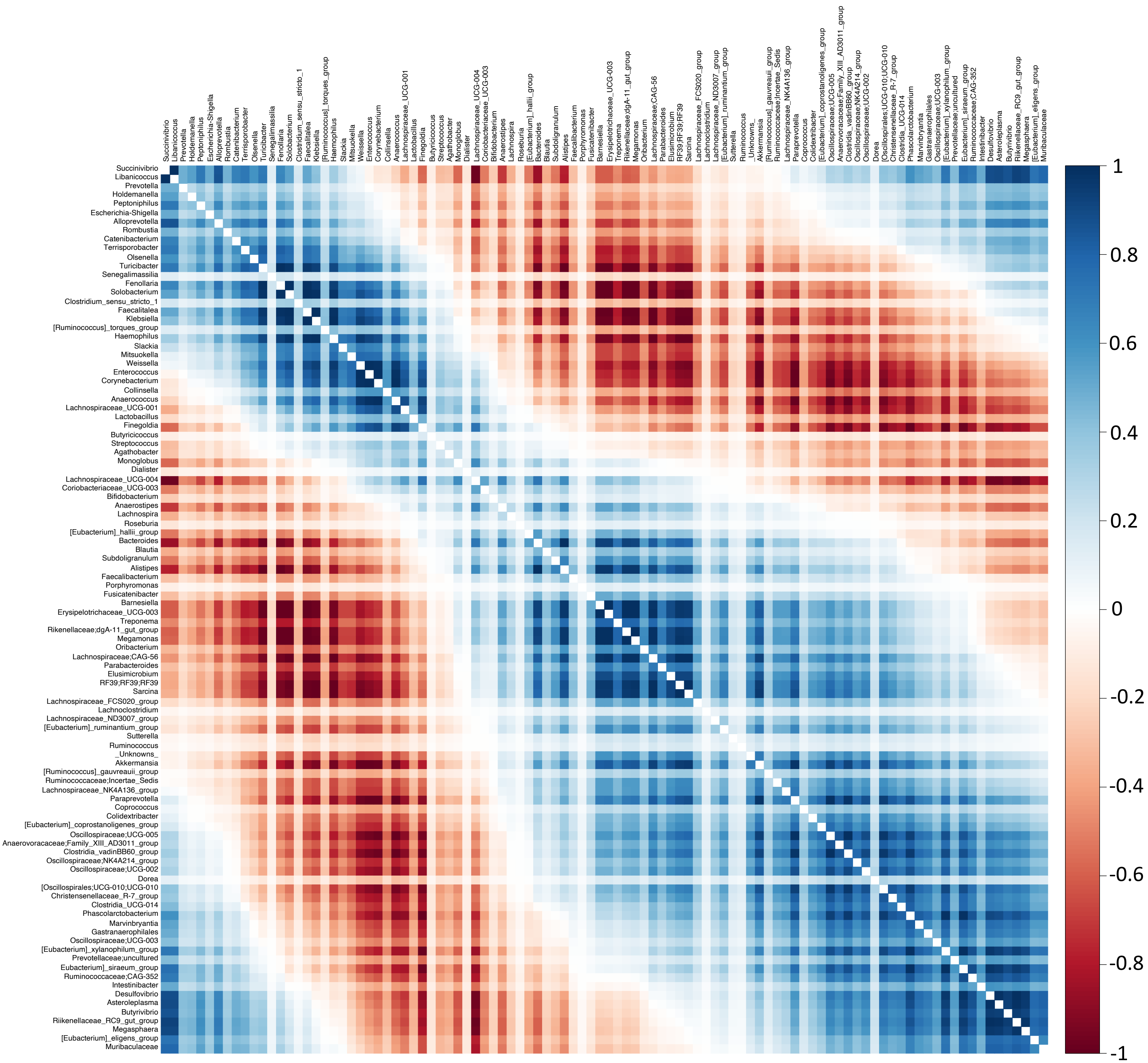

Fig. S47: Co-occurrence relationship between microbes recovered from the residual covariance matrix $\boldsymbol{\Sigma}$ that are not explained by the environmental covariates in the GLLVM model in Figures 2-4. Here, blue represent the positive correlation (taxa 1 increasing in abundance leads to taxa 2 increasing in abundance), and red represent the negative relationship (taxa 1 increasing in abundance leads to taxa 2 decreasing in abundance).

Table S1: PERMANOVA testing of no difference between two or more classes of objects (groups of samples) based on the analysis and partitioning sums of square distances as per sources of variability in the self-reported questionnaire. R^2 value where significant ($p<0.05$) represents the percentage variability explained by that covariate. For categorical variables, all possible outcomes are shown in brackets along with their frequencies. Important covariates where there are significant associations with microbiome are shown with a blue background. Here N.S represents non significance.

Covariate	Bray-Curtis (Composition)	Unweighted Unifrac (Phylogeny)	Weighted Unifrac (Composition weighted Phylogeny)	Heirarchical Meta-Storm (Function)
**Do you experience acid reflux?** (Yes:37; No:56)	N.S	N.S	N.S	N.S
**Age**	R^2 =0.013 (P=0.09.)	N.S	N.S	R^2 =0.020 (P=0.05.)
**Did you suffer from anemia in the past?** (Yes:10; No:83)	N.S	N.S	N.S	N.S
**Were you given antibiotics as a child?** (Yes:77; No:15; Maybe:1)	R^2 =0.033 (P=0.01*)	R^2 =0.074 (P=0.01**)	R^2 =0.074 (P=0.01*)	N.S
**Do you have trouble biting or chewing certain foods (apples, meat)?** (Yes:7; No:86)	N.S	N.S	N.S	N.S
**Do you have bad breath?** (Yes:9; No:84)	N.S	N.S	N.S	N.S
**Do you share your bathroom/toilet?** (Yes:87; No:6)	N.S	N.S	N.S	N.S
**Do you feel you are becoming healthier?** (Yes:56; No:37)	N.S	R^2 =0.021 (P=0.06.)	R^2 =0.021 (P=0.07.)	N.S
**Do you feel you are becoming unhealthier?** (Yes:24; No:69)	R^2 =0.016 (P=0.02*)	R^2 =0.026 (P=0.02*)	R^2 =0.026 (P=0.03*)	N.S
**Do you eat beef regularly?** (Yes:51; No:42)	N.S	N.S	N.S	N.S
**Birth city** (Quetta:10; Bagh:2; Swat:2; Islamabad:13; Jehlum:1; Rawalpindi:15; Mardan:3; Lahore:2; Sahiwal:5; Haripur:3; Chakwal:4; Karachi:2; Hasilpur:1; Abbotabad:1; Daharki:1; Lodhran:1; Nankana:1; Khanpur:1; Malakand:1; Kasur:1; Peshawar:3; Faislabad:1; Hyderabad:2;	N.S	N.S	N.S	N.S

Table S1: PERMANOVA testing of no difference between two or more classes of objects (groups of samples) based on the analysis and partitioning sums of square distances as per sources of variability in the self-reported questionnaire. R^2 value where significant ($p<0.05$) represents the percentage variability explained by that covariate. For categorical variables, all possible outcomes are shown in brackets along with their frequencies. Important covariates where there are significant associations with microbiome are shown with a blue background. Here N.S represents non significance.

Kashmir:1; Rawlakot, AJK:2; Jaranwala:1; Sialkot:3; Tehran:1; Mirpur Khas:1; Noshehra:1; Taunsa Sharif:1; Kot addu:1; Rawalakot:2; Kohat:1; Haveli:1; Mianwali:1)				
**What was your mode of birth?** (Natural:86; Cesarean:7)	N.S	R^2 =0.043 (P=0.01*)	R^2 =0.043 (P=0.01*)	N.S
**Body Mass Index (BMI)**	R^2 =0.017 (P=0.01*)	R^2 =0.055 (P=0.001***)	R^2 =0.055 (P=0.001***)	N.S
**How do you feel during bowel movement?** (Poor:8; Moderate:70; Good:15)	R^2 =0.028 (P=0.03*)	N.S	N.S	N.S
**Do you consume bread daily?** (Yes:56; No:37)	R^2 =0.015 (P=0.05.)	N.S	N.S	R^2 =0.021 (P=0.02*)
**Do you eat breakfast on at least 5 days each week?** (Yes:83; No:10)	N.S	N.S	N.S	N.S
**Were you breastfed as an infant?** (Yes:83; No:10)	N.S	R^2 =0.024 (P=0.09.)	R^2 =0.024 (P=0.09.)	N.S
**How often do you brush your teeth?** (Once a day:72; Twice a day:19; More than twice a day:2)	N.S	N.S	N.S	N.S
**Do you consume butter daily?** (Yes:26; No:67)	N.S	N.S	N.S	N.S
**Do you consume cheese daily?** (Yes:2; No:91)	R^2 =0.020 (P=0.01*)	N.S	N.S	N.S
**Do you eat chicken regularly?** (Yes:77; No:16)	N.S	N.S	N.S	N.S
**Did you suffer from parasitic infection (tapeworm etc.) during childhood?** (Yes:11; No:85)	R^2 =0.014 (P=0.04*)	N.S	N.S	N.S

Table S1: PERMANOVA testing of no difference between two or more classes of objects (groups of samples) based on the analysis and partitioning sums of square distances as per sources of variability in the self-reported questionnaire. R^2 value where significant ($p<0.05$) represents the percentage variability explained by that covariate. For categorical variables, all possible outcomes are shown in brackets along with their frequencies. Important covariates where there are significant associations with microbiome are shown with a blue background. Here N.S represents non significance.

**Do you have children?** (Yes:29; No:64)	R^2 =0.013 (P=0.09.)	R^2 =0.015 (P=0.08.)	N.S	N.S
**Do you consume coffee every day?** (Yes:15;No:78)	N.S	N.S	N.S	N.S
**Have you had your colon cleansed in the past week?** (Yes:7; No:86)	R^2 =0.014 (P=0.05.)	R^2 =0.057 (P=0.007**)	R^2 =0.057 (P=0.006**)	N.S
**During sampling, did you have Constipation?** (Yes:7; No:86)	N.S	N.S	N.S	N.S
**Have you ever used any medication or home remedies to prevent or treat constipation?** (Yes:16; No:77)	N.S	N.S	N.S	N.S
**Current city** (Quetta:6; Rawalpindi:19; Islamabad:55; Lahore:2; Sahiwal:2; Haripur:1; Daharki:1; Hyderabad:1; Mardan:1; Karachi:1; Noshehra:1; Rawalakot:2; Mianwali:1)	N.S	N.S	N.S	N.S
**Current health status** (Poor:4; Moderate:31; Good:55; Excellent:3)	N.S	N.S	N.S	N.S
**How often do you eat Samosa, pakora and other deep-fried food items in a week?** (Rarely:29; Once a week:33; Everyday:1; Between 2 to 4 times a week:30)	N.S	N.S	N.S	N.S
**Do you go to the dentist at least once a year?** (Yes:12; No:81)	N.S	N.S	N.S	R^2 =0.022 (P=0.03*)
**Do you consume desi ghee every day?** (Yes:8; No:85)	N.S	N.S	N.S	N.S
**Have you been diagnosed with any of the sexually transmitted diseases?** (Genital Herpes:1; None of the above:92)	N.S	N.S	N.S	N.S
**What best describe your diet?** (All Meat & Vegetables:78; No Beef & Mutton but Chicken	R^2 =0.026 (P=0.09.)	N.S	N.S	R^2 =0.034 (P=0.09.)

Table S1: PERMANOVA testing of no difference between two or more classes of objects (groups of samples) based on the analysis and partitioning sums of square distances as per sources of variability in the self-reported questionnaire. R^2 value where significant ($p<0.05$) represents the percentage variability explained by that covariate. For categorical variables, all possible outcomes are shown in brackets along with their frequencies. Important covariates where there are significant associations with microbiome are shown with a blue background. Here N.S represents non significance.

& Fish:12; All Meat & Vegetables but no Dairy:3)				
**How often do you eat dinner outside instead of eating/cooking at home?** (Rarely:42; Once a week:25; between 2 to 4 times a week:17; Everyday:9)	R^2 =0.041 (P=0.02*)	N.S	N.S	N.S
**Do you eat dinner on at least 5 days each week?** (Yes:89; No:4)	N.S	N.S	N.S	N.S
**During sampling, did you have dizziness?** (Yes:2; No:92)	N.S	N.S	N.S	N.S
**What is your main source of drinking water?** (Tap:21; Filtered:60; Bottled:6; Mineral:3; Natural Spring:3)	N.S	N.S	N.S	N.S
**Is your mouth dry?** (Yes:5; No:88)	N.S	N.S	N.S	R^2 =0.023 (P=0.07.)
**Do you consume dry fruits 2 or more times a week?** (Yes:43; No:50)	R^2 =0.014 (P=0.08.)	N.S	N.S	N.S
**What is the highest level of education you completed?** (Intermediate:11; Undergraduate:34; Masters:45; Doctorate:2; Primary:1)	N.S	N.S	N.S	N.S
**Do you consume eggs every day?** (Yes:53; No:40)	R^2 =0.014 (P=0.06.)	N.S	N.S	N.S
**What best describes your employment status?** (Unemployed:20; Student:43; Full time:20; Self employed:9; Part time:1)	R^2 =0.051 (P=0.06.)	N.S	N.S	N.S

Table S1: PERMANOVA testing of no difference between two or more classes of objects (groups of samples) based on the analysis and partitioning sums of square distances as per sources of variability in the self-reported questionnaire. R^2 value where significant ($p<0.05$) represents the percentage variability explained by that covariate. For categorical variables, all possible outcomes are shown in brackets along with their frequencies. Important covariates where there are significant associations with microbiome are shown with a blue background. Here N.S represents non significance.

**Ethnicity** (Balochi:7; Kashmiri:9; Pathan:21; Urdu speaking:6; Punjabi:37; Hazary wal:1; Sindhi:6; Saraiki:4; Pashtun:1; Hazara:1)	R^2 =0.11537 (P=0.003**)	N.S	N.S	N.S
**How much healthy have you felt in the past 1 week?** (Moderately:69; Not at all:4; Slightly:13; Extremely:7)	R^2 =0.047 (P=0.004**)	N.S	N.S	N.S
**Do you eat fish regularly?** (Yes:25; No:68)	N.S	N.S	N.S	N.S
**Do you eat fresh fruits every day?** (Yes:58; No:35)	R^2 =0.017 P=(0.01*)	N.S	N.S	N.S
**Gender** (Male:43; Female:50)	R^2 =0.023 (P=0.001***)	R^2 =0.034 (P=0.01*)	R^2 =0.034 (P=0.01*)	R^2 =0.017 (P=0.06.)
**Do you experience gut flare-ups (Sudden pain, or outburst in gut)?** (Yes:18; No:75)	N.S	N.S	N.S	N.S
**During sampling, did you have headache?** (Yes:8; No:85)	N.S	N.S	N.S	N.S
**Do you consume honey regularly?** (Yes:13; No:80)	R^2 =0.016 (P=0.02*)	R^2 =0.034 (P=0.01*)	R^2 =0.034 (P=0.01*)	N.S
**Do you have pets?** (Yes:13; No:80)	R^2 = 0.017 (P=0.01*)	N.S	N.S	N.S
**During sampling, did you have insomnia?** (Yes:1; No:92)	N.S	N.S	N.S	N.S
**Do you consume "junk food" (e.g. fried chicken burgers, pizza) at least twice every week?** (Yes:47; No:46)	N.S	N.S	N.S	N.S
**Do you have lactose sensitivity?** (Yes:2; No:91)	N.S	N.S	N.S	N.S
**Do you consume lassi every day?** (Yes:21; No:72)	N.S	N.S	N.S	N.S
**Do you consume legumes?** (Yes:10; No:83)	N.S	N.S	N.S	N.S

Table S1: PERMANOVA testing of no difference between two or more classes of objects (groups of samples) based on the analysis and partitioning sums of square distances as per sources of variability in the self-reported questionnaire. R^2 value where significant ($p<0.05$) represents the percentage variability explained by that covariate. For categorical variables, all possible outcomes are shown in brackets along with their frequencies. Important covariates where there are significant associations with microbiome are shown with a blue background. Here N.S represents non significance.

**Do you experience lethargy or general malaise?** (Yes:13; No:80)	N.S	N.S	N.S	N.S
**Do you have contact with livestock?** (Yes:10; No:83)	N.S	N.S	N.S	N.S
**Do you eat lunch on at least 5 days each week?** (Yes:83; No:10)	R^2 =0.014 (P=0.06.)	N.S	N.S	N.S
**Are you allergic to milk?** (Yes:2; No:91)	N.S	N.S	N.S	N.S
**Do you consume milk every day?** (Yes:52; No:41)	N.S	N.S	N.S	N.S
**What is your monthly income (or income of parent/guardian)?** (Between 60,000 & 120,000:38; Between 20,000 & 60,000:35; >120,000:17; <20,000:3)	N.S	R^2 =0.065 (P=0.03*)	R^2 =0.065 (P=0.04*)	N.S
**Do you eat goat and/or lamb meat regularly?** (Yes:50; No:43)	R^2 =0.013 (P=0.09.)	N.S	N.S	N.S
**Do you eat naan/roti on at least 5 days each week?** (Yes:92; No:1)	N.S	N.S	N.S	R^2 =0.086 (P=0.004**)
**Do you take naps?** (No never:19;Yes usually:16; Yes sometimes:58)	N.S	R^2 =0.036 (P=0.08.)	R^2 =0.036 (P=0.07.)	N.S
**Did you have any craving for soil/chalk/sand/ or other non-food items in the past?** (Yes:14; No:79)	N.S	N.S	N.S	N.S
**Do you consume oatmeal every day?** (Yes:15; No:78)	N.S	N.S	N.S	N.S
**What is your occupation (or occupation of parent/legal guardian)? (**Personal business:41; Labor force:4; Academia:23; Unemployed:11; Academia Agriculture:4; Government:4; Military:2; Foreign remittance:2; Multinational:1; Agriculture:1)	N.S	N.S	N.S	N.S

Table S1: PERMANOVA testing of no difference between two or more classes of objects (groups of samples) based on the analysis and partitioning sums of square distances as per sources of variability in the self-reported questionnaire. R^2 value where significant ($p<0.05$) represents the percentage variability explained by that covariate. For categorical variables, all possible outcomes are shown in brackets along with their frequencies. Important covariates where there are significant associations with microbiome are shown with a blue background. Here N.S represents non significance.

**Had you undergone any treatment for parasitic infection?** (Yes:8; No:85)	R^2 =0.018 (P=0.007**)	N.S	N.S	N.S
**Do a family member or friend you are in constant contact with (>2 hours a day) smoke in your presence?** (Yes:15; No:78)	N.S	R^2 =0.010 (P=0.03*)	N.S	R^2 =0.031 (P=0.01*)
**Are you allergic to peanuts?** (Yes:3; No:90)	N.S	N.S	N.S	N.S
**How do you identify yourself as a person?** (Social with only close friends:29; Very social and friendly:27; Sometimes social sometimes friendly:31; Stressed at gatherings:1; Shy and introvert:5)	N.S	R^2 = 0.086 (P=0.04*)	R^2 = 0.086 (P=0.03*)	N.S
**Do you consume pickle every day?** (Yes:7; No:86)	R^2 = 0.028 (P=0.001***)	R^2 = 0.033 (P=0.07.)	R^2 = 0.033 (P=0.07.)	N.S
**How often do you poop?** (Once everyday:74; Thrice a week:13; Twice a day:6)	N.S	N.S	N.S	N.S
**Birth province** (Balochistan:10; AJK:8; KPK:15; ICT:14; Punjab:40; Sindh:6)	R^2 = 0.071 (P=0.003**)	N.S	N.S	N.S
**Residence province [**Balochistan:6; Punjab:24; Islamabad Capital Territory (ICT):55; Khyber Pakhtunkhwa (KPK):3; Sindh:3; Azad Jammu & Kashmir (AJK):2]	R^2 =0.077 (P=0.002**)	N.S	N.S	N.S
**Have you felt abdominal pain in the last 2 days?** (Not recently:65; Yes moderately:14; Yes slightly:13; Yes extremely:1)	N.S	N.S	N.S	N.S

Table S1: PERMANOVA testing of no difference between two or more classes of objects (groups of samples) based on the analysis and partitioning sums of square distances as per sources of variability in the self-reported questionnaire. R^2 value where significant ($p<0.05$) represents the percentage variability explained by that covariate. For categorical variables, all possible outcomes are shown in brackets along with their frequencies. Important covariates where there are significant associations with microbiome are shown with a blue background. Here N.S represents non significance.

**In the last two days, how much have you exercised?** (No:79; Less than 30 min:4; Between 30 & 60 mins:7; Between 1 & 2 hours:1; More than 2 hours:2)	N.S	N.S	N.S	N.S
**Have you had any headaches in the past 1 week?** (Yes regularly:2; Yes sometimes:64; No:27)	N.S	N.S	N.S	N.S
**How anxious have you felt in the past 1 week?** (Extremely:8; Moderately:27; Not at all:18; Slightly:40)	N.S	N.S	N.S	N.S
**How much happy have you felt in the past 1 week?** (Moderately:73; Extremely:10; Slightly:7; Not at all:3)	N.S	N.S	N.S	N.S
**How much stressed have you felt in the past 1 week?** (Moderately:38; Extremely:5; Slightly:40; Not at all:10)	R^2 =0.038 (P=0.08.)	N.S	N.S	N.S
**How much tired have you felt in the past 1 week?** (Moderately:40; Extremely:22; Slightly:24; Not at all:7)	R^2 =0.041 (P=0.01*)	R^2 =0.053 (P=0.07.)	R^2 =0.053 (P=0.07.)	N.S
**Do you exercise regularly?** (Yes:15; No:78)	N.S	N.S	N.S	N.S
**Do you regularly experience throat issues (e.g. sore throat, flu)?** (Yes:34; No:59)	R^2 =0.016 (P=0.02*)	N.S	N.S	R^2 =0.019 (P=0.04*)
**Did you get your appendix removed?** (Yes:5; No:88)	N.S	N.S	N.S	N.S
**Have you had your tonsils removed?** (Yes:7; No:86)	N.S	R^2 =0.036 (P=0.03*)	R^2 =0.036 (P=0.04*)	N.S

Table S1: PERMANOVA testing of no difference between two or more classes of objects (groups of samples) based on the analysis and partitioning sums of square distances as per sources of variability in the self-reported questionnaire. R^2 value where significant ($p<0.05$) represents the percentage variability explained by that covariate. For categorical variables, all possible outcomes are shown in brackets along with their frequencies. Important covariates where there are significant associations with microbiome are shown with a blue background. Here N.S represents non significance.

**Do you eat rice regularly?** (Yes:87; No:6)	N.S	R^2 =0.025 (P=0.09.)	R^2 =0.025 (P=0.08.)	R^2 =0.020 (P=0.05.)
**How often do you get sick?** (Sometimes:41; Rarely:49; Frequently:3)	N.S	N.S	N.S	N.S
**How much sleep do you get every night, on average?** (Between 6 to 8 hours:58; Between 4 to 6 hours:29; More than 8 hours:4; Less than 4 hours:2)	N.S	N.S	N.S	N.S
**Do you smoke regularly?** (Yes:9; No:84)	N.S	N.S	N.S	N.S
**What best describes your socioeconomic status?** (Middle class:39; Lower middle class:34; Upper middle class:17; Lower class:3)	N.S	R^2 =0.066 (P=0.04*)	R^2 =0.066 (P=0.05*)	N.S
**Do you consume soft cheese every day?** (Yes:7; No:86)	R^2 = 0.028 (P=0.001***)	R^2 = 0.033 (P=0.05.)	R^2 = 0.033 (P=0.07.)	N.S
**During sampling, did you have stomach pain?** (Yes:1; No:92)	N.S	R^2 =0.044 (P=0.03*)	R^2 =0.044 (P=0.03*)	N.S
**How often do you take juices or other sugary drinks (e.g. lemonade, Rooh Afza, soft drinks, etc.) in a week?** (Rarely:17; Between 2 to 4 times a week:47; Once a week:19; Everyday:10)	N.S	N.S	N.S	N.S
**How often do you eat sugary foods (e.g. halwa, desserts, kheer, ice-cream, etc.) in a**	N.S	N.S	N.S	N.S

Table S1: PERMANOVA testing of no difference between two or more classes of objects (groups of samples) based on the analysis and partitioning sums of square distances as per sources of variability in the self-reported questionnaire. R^2 value where significant ($p<0.05$) represents the percentage variability explained by that covariate. For categorical variables, all possible outcomes are shown in brackets along with their frequencies. Important covariates where there are significant associations with microbiome are shown with a blue background. Here N.S represents non significance.

**week?** (Once a week:38; Between 2 to 4 times a week:47; Rarely:17; Everyday:10)				
**Do you consume tea every day?** (Yes:82; No:11)	N.S	N.S	N.S	N.S
**Do you have trouble falling asleep?** (No Never:35; Yes always:8; Yes sometimes:46; Yes usually:4)	R^2 = 0.042 (P=0.01*)	N.S	N.S	N.S
**Do you have trouble swallowing?** (Yes:4; No:89)	N.S	N.S	N.S	N.S
**Have you been vaccinated with Bacillus Calmette-Guérin (BCG) vaccine?** (Yes:27; No:66)	N.S	R^2 =0.020 (P=0.08.)	R^2 =0.020 (P=0.08.)	N.S
**Have you been vaccinated with COVID-19 vaccine?** (Yes:71; No:22)	N.S	N.S	N.S	N.S
**Have you been vaccinated with Hepatitis vaccine?** (Yes:7; No:86)	N.S	R^2 =0.026 (P=0.09.)	N.S	N.S
**Have you been vaccinated with Measles, Mumps, and Rubella (MMR) vaccine?** (Yes:20; No:73)	N.S	R^2 =0.020 (P=0.08.)	R^2 =0.020 (P=0.08.)	N.S
**Have you been vaccinated with Polio vaccine?** (Yes:29; No:64)	R^2 = 0.014 (P=0.04*)	N.S	N.S	N.S
**Have you been vaccinated with Rabies vaccine?** (Yes:5; No:88)	N.S	N.S	N.S	N.S
**Do you eat vegetables regularly?** (Yes:83; No:10)	N.S	N.S	N.S	N.S
**How many hours per week do you work?** (20 to 40:42; 40 to 60:23; Less than 20:20; More than 60:8)	N.S	N.S	N.S	N.S
**Did you gain weight recently?** (Yes:21; No:72)	N.S	N.S	N.S	N.S

Table S1: PERMANOVA testing of no difference between two or more classes of objects (groups of samples) based on the analysis and partitioning sums of square distances as per sources of variability in the self-reported questionnaire. R^2 value where significant ($p<0.05$) represents the percentage variability explained by that covariate. For categorical variables, all possible outcomes are shown in brackets along with their frequencies. Important covariates where there are significant associations with microbiome are shown with a blue background. Here N.S represents non significance.

**Did you lose weight recently?** (Yes:3; No:90)	N.S	N.S	N.S	N.S
**Do you consume yogurt every day?** (Yes:56; No:37)	N.S	N.S	N.S	N.S
**.p<0.1* p<0.05 ** p<0.01 *** p<0.001**				

Table S2: Literature survey significant genera (top 5 most positive and negative beta coefficients) in GLLVM associated with all sources of variability. The acronyms are as follows: (ICT: Islamabad Capital Territory; AJK: Azad Jammu & Kashmir; KPK: Khyber Pakhtunkhwa; LC: Lower Class; LMC: Lower Middle Class; MC: Middle Class; UMC: Upper Middle Class).

No	Significant genera	Positively associated covariates	Negatively associated covariates	Implication in literature	Reference
1.	Lachnospiraceae;CAG−56	Age, [Province of birth:KPK; REF(ICT)], [Source of Drinking Water: Bottled/ Mineral; REF(Filtered)] SoftCheese Consumption:Yes, Desighee Consumption:Yes, Lassi Consumption:Yes, Fresh Fruits Consumption:Yes, Fried Food Items:Yes, Junk Food Twice/ Week:Yes	[Gender:Male; REF(Female)] [Province of Birth:AJK/Sindh; REF(ICT)], [Province of Residence:Balochistan/Punjab; REF(ICT)], [Source of Drinking Water: Tap; REF(Filtered)] [Education:Intermediate/Doctorate; REF(Primary)] [Socioeconomic Status:LMC; (REF(LC)], Cheese Consumption:Yes, Milk Consumption:Yes, Yogurt Consumption:Yes, Honey Consumption:Yes, Coffee Consumption:Yes, Vegetables Consumption:Yes, [Sugary Foods Consumption: Once/Week/Between 2 to 4 times/Week/Everyday; REF(Rarely)]	Members of Lachnospiraceae are strictly anaerobic, SCFA producers and are detected in humans of different age groups *Lachnospiraceae;CAG* has been reported to be associated with fast food consumption, high fiber diet and complex carbohydrates	(*1-3*)
2.	Phascolarctobacterium	Age, Gender:Male; REF(Female)] [Province of Birth:AJK/KPK/Punjab/Sindh; REF(ICT)], Province of Residence:KPK; REF(ICT)], [Socioeconomic Status:LMC; (REF(LC)], Yogurt Consumption:Yes, Lassi Consumption:Yes, Honey Consumption:Yes, Naan/Roti 5 days/week:Yes, [Deep Fried Food Consumption: Between 2 to 4 times/ Week; REF(Rarely)]	[Province of Residence:Punjab/Sindh; REF(ICT)], [Source of Drinking Water: Natural_spring; REF(Filtered)] [Education:Undergraduate/Masters; REF(Primary)] Chicken Consumption:Yes, Beef Consumption:Yes, Milk Consumption:Yes, Buttermilk Consumption:Yes, Desighee Consumption:Yes, Rice Consumption:Yes, Fresh Fruits Consumption:Yes	SCFA producers and have beneficial effects in human gut health. Higher abundance in age group 18-40 years Higher abundance is also associated with high fat diet, starchy foods, and dairy Higher abundance associated with tap drinking water in Pakistan	(*4-8*)

Table S2: Literature survey significant genera (top 5 most positive and negative beta coefficients) in GLLVM associated with all sources of variability. The acronyms are as follows: (ICT: Islamabad Capital Territory; AJK: Azad Jammu & Kashmir; KPK: Khyber Pakhtunkhwa; LC: Lower Class; LMC: Lower Middle Class; MC: Middle Class; UMC: Upper Middle Class).

3.	Solobacterium	Age, [Province of Birth:Punjab; REF(ICT)], [Province of Residence:Balochistan/KPK/Sindh; REF(ICT)], [Source of Drinking Water: Bottled; REF(Filtered)] [Education:Intermediate; REF(Primary)] Chicken Consumption:Yes, Yogurt Consumption:Yes, Fried Food Items:Yes, [Deep Fried Food Consumption: Once/Week/Everyday; REF(Rarely)]	[Province of Birth:KPK; REF(ICT)], Mutton Consumption:Yes, SoftCheese Consumption:Yes, Breakfast 5 days/week:Yes, Naan/Roti 5 days/week:Yes, Junk Food Twice/ Week:Yes, [Sugary Foods Consumption: Between 2 to 4 times/Week; REF(Rarely)]	Gram-positive, non-spore-forming, obligate anaerobic which causes halitosis (foul smell or oral malodour) and oral infections<br>Found to be present in all age group between 11-65 years	(*9, 10*)
4.	Haemophilus	Age, BMI, [Province of Birth:Balochistan; REF(ICT)] [Province of Residence:AJK; REF(ICT)], [Source of Drinking Water: Bottled; REF(Filtered)] SoftCheese Consumption:Yes, Eggs Consumption:Yes, Coffee Consumption:Yes, [Deep Fried Food Consumption: Between 2 to 4 times/ Week; REF(Rarely)]	[Gender: Male; REF(Female)] [Province of Residence:Punjab/Sindh; REF(ICT)], [Source of Drinking Water: Mineral/Tap; REF(Filtered)] [Education:Undergraduate/Masters; REF(Primary)] Fish Consumption:Yes, Bread Consumption:Yes, Lassi Consumption:Yes, Oatmeal Consumption:Yes, Pickle Consumption:Yes, Tea Consumption:Yes, Fresh Fruits Consumption:Yes, Breakfast 5 days/week:Yes, [Sugary Foods Consumption: Once/Week/ Between 2 to 4 times/Week; REF(Rarely)]	Part of healthy human salivary microbiome. Some of the species are respiratory tract pathogens<br>Found positively associated with tea and coffee consumption	(*11-13*)

Table S2: Literature survey significant genera (top 5 most positive and negative beta coefficients) in GLLVM associated with all sources of variability. The acronyms are as follows: (ICT: Islamabad Capital Territory; AJK: Azad Jammu & Kashmir; KPK: Khyber Pakhtunkhwa; LC: Lower Class; LMC: Lower Middle Class; MC: Middle Class; UMC: Upper Middle Class).

5.	Klebsiella	BMI, [Source of Drinking Water: Mineral/Tap; REF(Filtered)] Bread Consumption:Yes, Cheese Consumption:Yes, SoftCheese Consumption:Yes, Honey Consumption:Yes, Junk Food Twice/ Week:Yes	Age, [Province of Birth:Balochistan; REF(ICT)] Province of Birth:Punjab; REF(ICT)], [Source of Drinking Water: Bottled; REF(Filtered)] Butter Consumption:Yes, Milk Consumption:Yes, Yogurt Consumption:Yes, Oatmeal Consumption:Yes, Coffee Consumption:Yes, Tea Consumption:Yes, Fried Food Items:Yes	Gram-negative non motile pathogenic bacteria, can cause inflammation in the gut<br>Higher abundance associated with obesity<br>*Klebsiella* species i.e., *Klebsiella grimontii*, *Klebsiella oxytoca* and *pneumonia* were found to be present in tradionally made cheese	(*14-17*)
6.	Coriobacteriaceae_UCG−003	BMI, Fish Consumption:Yes	Age, Beef Consumption:Yes, Tea Consumption:Yes, Dry Fruits Consumption:Yes, Lunch 5 days/week:Yes, Junk Food Twice/ Week:Yes	Coriobacteriaceae play an important role in conversion of bile salts and steroids and activation of dietary polyphenols.	(*18*)
7.	Finegoldia	BMI, [Province of Birth:AJK; REF(ICT)] [Province of Residence:Punjab; REF(ICT)], [Socioeconomic Status:UMC; (REF(LC)], Mutton Consumption:Yes, Cheese Consumption:Yes, Desighee Consumption:Yes, Pickle Consumption:Yes, Dry Fruits Consumption:Yes, Naan/Roti 5 days/week:Yes, [Deep Fried Food Consumption: Once/Week/ Between 2 to 4 times/Week; REF(Rarely)] [Sugary Foods Consumption: Once/Week/ Between 2 to 4 times/Week/Everyday; REF(Rarely)]	Age, [Province of Birth:Balochistan/KPK/Sindh; REF(ICT)] [Source of Drinking Water: Mineral; REF(Filtered)] [Education:Undergraduate/Masters/Doctorate; REF(Primary)] [Socioeconomic Status:MC; (REF(LC)], Chicken Consumption:Yes, SoftCheese Consumption:Yes, Buttermilk Consumption:Yes, Cream Consumption:Yes, Yogurt Consumption:Yes, Lassi Consumption:Yes, Eggs Consumption:Yes, Fresh Fruits Consumption:Yes	Gram-positive anaerobic cocci, colonizes the skin and other non-sterile body surfaces<br>Found to be associated with high BMI and consumption of sweets	(*19-22*)

Table S2: Literature survey significant genera (top 5 most positive and negative beta coefficients) in GLLVM associated with all sources of variability. The acronyms are as follows: (ICT: Islamabad Capital Territory; AJK: Azad Jammu & Kashmir; KPK: Khyber Pakhtunkhwa; LC: Lower Class; LMC: Lower Middle Class; MC: Middle Class; UMC: Upper Middle Class).

8.	Akkermansia	[Province of Residence:Punjab; REF(ICT)], [Source of Drinking Water: Tap; REF(Filtered)] Yogurt Consumption:Yes, Oatmeal Consumption:Yes, Breakfast 5 days/week:Yes, Lunch 5 days/week:Yes, [Sugary Foods Consumption: Once/Week; REF(Rarely)]	Age, BMI, Gender:Male; REF(Female)] Fried Food Items:Yes	Gram-negative anaerobes, Higher abundance associated with yogurt and sweets/sugary food consumption Decreased abundance observed in obesity and metabolic disorders	(*22-24*)
9.	Anaerococcus	[Source of Drinking Water: Natural_spring; REF(Filtered)] Mutton Consumption:Yes, Milk Consumption:Yes, Oatmeal Consumption:Yes, Pickle Consumption:Yes, Dry Fruits Consumption:Yes, [Sugary Foods Consumption: Once/Week; REF(Rarely)]	Age, Chicken Consumption:Yes, Fish Consumption:Yes, Butter Consumption:Yes, Yogurt Consumption:Yes, Eggs Consumption:Yes, [Deep Fried Food Consumption: Between 2 to 4 times/ Week; REF(Rarely)]	Anaerobic, Gram-positive bacteria commonly associated with gastrointestinal and urogenital disorders No association found with the covariates	(*25*)
10.	Turicibacter	BMI, [Province of Birth:AJK/Balochistan; REF(ICT)], [Socioeconomic Status:MC; REF(LC)], Cheese Consumption:Yes, SoftCheese Consumption:Yes, Lassi Consumption:Yes, Honey Consumption:Yes, [Deep Fried Food Consumption: Everyday; REF(Rarely)]	[Province of Residence:Balochistan/Punjab; REF(ICT)], [Source of Drinking Water: Natural_spring; REF(Filtered)] [Education:Undergraduate/Masters REF(Primary)] Butter Consumption:Yes, Cream Consumption:Yes, Desighee Consumption:Yes, Coffee Consumption:Yes, Breakfast 5 days/week:Yes, [Deep Fried Food Consumption: Once/Week; REF(Rarely)] [Sugary Foods Consumption: Between 2 to 4 times/Week; REF(Rarely)]	Anaerobic gram-positive, butyrate producing bacteria. Higher abundance showed association with high-fat diet and obesity in mice model	(*26*)

Table S2: Literature survey significant genera (top 5 most positive and negative beta coefficients) in GLLVM associated with all sources of variability. The acronyms are as follows: (ICT: Islamabad Capital Territory; AJK: Azad Jammu & Kashmir; KPK: Khyber Pakhtunkhwa; LC: Lower Class; LMC: Lower Middle Class; MC: Middle Class; UMC: Upper Middle Class).

11.	Sarcina	[Province of Residence:AJK; REF(ICT)], Fish Consumption:Yes, Bread Consumption:Yes, Butter Consumption:Yes, Desighee Consumption:Yes, Yogurt Consumption:Yes, Junk Food Twice/ Week:Yes	BMI, Milk Consumption:Yes, Fried Food Items:Yes, [Sugary Foods Consumption: Once/Week; REF(Rarely)]	Gram-positive anaerobic cocci are member of family clostridiaceae<br>Has been reported to maintain BMI	(*27*)
12.	Asteroleplasma	[Province of Birth:Balochistan; REF(ICT)], [Province of Residence:KPK/Sindh REF(ICT)], [Source of Drinking Water: Tap; REF(Filtered)] [Socioeconomic Status:MC; REF(LC)], Bread Consumption:Yes, Eggs Consumption:Yes, Oatmeal Consumption:Yes, Fresh Fruits Consumption:Yes, Breakfast 5 days/week:Yes, Lunch 5 days/week:Yes, Junk Food Twice/ Week:Yes	BMI, [Province of Birth:AJK/KPK/Punjab; REF(ICT)] [Province of Residence:Balochistan; REF(ICT)], [Source of Drinking Water: Bottled; REF(Filtered)] Mutton Consumption:Yes, Buttermilk Consumption:Yes, Cream Consumption:Yes, Naan/Roti 5 days/week:Yes, [Deep Fried Food Consumption: Once/Week/ Between 2 to 4 times/Week; REF(Rarely)]	Obligate anaerobes, decreased abundance has been observed in high fat diet group in Chinese community<br>Role in human body not well studied yet	(*28, 29*)
13.	Barnesiella	[Source of Drinking Water: Natural_spring; REF(Filtered)] Chicken Consumption:Yes, Rice Consumption:Yes, Coffee Consumption:Yes, Tea Consumption:Yes, Breakfast 5 days/week:Yes	BMI, Bread Consumption:Yes, Oatmeal Consumption:Yes, Junk Food Twice/ Week:Yes, [Sugary Foods Consumption: Once/Week; REF(Rarely)]	Protect against the antibiotic-resistant pathogenic bacteria and have been reported abundantly present in healthy group as compared to the subjects with gastrointestinal diseases<br>Increased abundance reported after oatmeal consumption	(*30-32*)

Table S2: Literature survey significant genera (top 5 most positive and negative beta coefficients) in GLLVM associated with all sources of variability. The acronyms are as follows: (ICT: Islamabad Capital Territory; AJK: Azad Jammu & Kashmir; KPK: Khyber Pakhtunkhwa; LC: Lower Class; LMC: Lower Middle Class; MC: Middle Class; UMC: Upper Middle Class).

14.	Erysipelotrichaceae_UCG−003	[Education:Intermediate; REF(Primary)] Beef Consumption:Yes, Bread Consumption:Yes, Butter Consumption:Yes, Milk Consumption:Yes, Pickle Consumption:Yes, Rice Consumption:Yes, Fresh Fruits Consumption:Yes, Breakfast 5 days/week:Yes, [Deep Fried Food Consumption: Once/Week; REF(Rarely)] [Sugary Foods Consumption: Everyday; Reference(Rarely)]	BMI, [Province of Birth:AJK; REF(ICT)], [Province of Residence:KPK; REF(ICT)], [Source of Drinking Water: Bottled/Mineral; REF(Filtered)] Mutton Consumption:Yes, Cheese Consumption:Yes, Lassi Consumption:Yes, Eggs Consumption:Yes, Naan/Roti 5 days/week:Yes, [Deep Fried Food Consumption: Between 2 to 4 times/ Week; REF(Rarely)]	Increased abundance of *Erysipelotrichaceae* observed in mice on western or high-fat diet *Erysipelotrichaceae_UCG−003* highest abundance observed with high fiber intake and negative association with BMI Consumption of dairy products also increased the abundance of this genera	(*33-36*)
15.	Treponema	[Gender:Male; REF(Female)] Fish Consumption:Yes, Milk Consumption:Yes, Eggs Consumption:Yes	[Socioeconomic Status:UMC; REF(LC)], Bread Consumption:Yes, Oatmeal Consumption:Yes, Rice Consumption:Yes, Vegetables Consumption:Yes, [Deep Fried Food Consumption: Once/Week; REF(Rarely)]	Commensals of the oral microflora, Obligate anaerobes but pathogenic species can be microaerophilic Higher abundance has been observed in African population consuming fish and legumes based diet	(*37, 38*)
16.	Lachnospiraceae_UCG−004	[Gender:Male; REF(Female)] [Province of Residence:AJK/Balochistan; REF(ICT)], [Socioeconomic Status:LMC/MC; REF(LC)], Fish Consumption:Yes, Cheese Consumption:Yes, Oatmeal Consumption:Yes, Coffee Consumption:Yes, Tea Consumption:Yes, Lunch 5 days/week:Yes, [Sugary Foods Consumption: Everyday; REF(Rarely)]	[Province of Birth:AJK/Balochistan/KPK/Punjab/Sindh REF(ICT)], [Province of Residence:Sindh; REF(ICT)], [Education:Masters; REF(Primary)] Beef Consumption:Yes, Buttermilk Consumption:Yes, Cream Consumption:Yes, Dry Fruits Consumption:Yes	*Lachnospiraceae* family members have the ability to hydrolyze the starch and carbohydrates and produce butyrate and other SCFAs In vitro based study showed higher abundance of *Lachnospiraceae_UCG−004* after animal based food fermentation Higher abundance found to be associated with high socioeconomic status Positively associated with intake of dietary fiber, carbohydrates and plant protein Negatively associated with animal protein intake	(*36, 39, 40*)

Table S2: Literature survey significant genera (top 5 most positive and negative beta coefficients) in GLLVM associated with all sources of variability. The acronyms are as follows: (ICT: Islamabad Capital Territory; AJK: Azad Jammu & Kashmir; KPK: Khyber Pakhtunkhwa; LC: Lower Class; LMC: Lower Middle Class; MC: Middle Class; UMC: Upper Middle Class).

17.	Oribacterium	[Gender:Male; REF(Female)] [Province of Birth:KPK/Punjab; REF(ICT)], [Education:Masters; REF(Primary)] Chicken Consumption:Yes, Butter Consumption:Yes, Coffee Consumption:Yes, Fried Food Items:Yes, [Sugary Foods Consumption: Once/Week/Between 2 to 4 times/Week; REF(Rarely)]	[Province of Residence:Punjab; REF(ICT)], [Socioeconomic Status:LMC/MC/UMC; REF(LC)], Mutton Consumption:Yes, Lassi Consumption:Yes, Rice Consumption:Yes, Tea Consumption:Yes, Lunch 5 days/week:Yes, Junk Food Twice/ Week:Yes	Strictly anaerobic non-spore forming bacteria in oral cavity. Found abundantly present in males oral microbiome as compared to females	(*41, 42*)
18.	Rikenellaceae_RC9_ gut_group	[Gender:Male; REF(Female)] [Province of Birth:AJK; REF(ICT)], Naan/Roti 5 days/week:Yes, [Sugary Foods Consumption: Everyday; REF(Rarely)]	SoftCheese Consumption:Yes, [Deep Fried Food Consumption: Everyday; REF(Rarely)]	Helps in lipid metabolism and digestion of crude fiber<br>Higher abundance observed in mice with high fat diet	(*43-45*)
19.	Elusimicrobium	[Source of Drinking Water: Bottled; REF(Filtered)], Eggs Consumption:Yes, Rice Consumption:Yes, Coffee Consumption:Yes, Vegetables Consumption:Yes, [Deep Fried Food Consumption: Between 2 to 4 times/ Week; REF(Rarely)]	Gender:Male; REF(Female)], [Province of Birth:Punjab; REF(ICT)] Province of Residence:Balochistan; REF(ICT)], [Source of Drinking Water: Tap; REF(Filtered)] [Education:Undergraduate; REF(Primary)] [Socioeconomic Status:LMC; REF(LC)], Beef Consumption:Yes, Bread Consumption:Yes, Honey Consumption:Yes, Oatmeal Consumption:Yes, Tea Consumption:Yes, [Sugary Foods Consumption: Everyday; REF(Rarely)]	Observed higher abundance in males as compared to females in Pakistani population	(*46*)
20.	Fenollaria	Beef Consumption:Yes, Fried Food Items:Yes	[Gender:Male; REF(Female)] Honey Consumption:Yes	Novel bacteria. Some species are isolated from healthy human fecal samples	(*47*)

Table S2: Literature survey significant genera (top 5 most positive and negative beta coefficients) in GLLVM associated with all sources of variability. The acronyms are as follows: (ICT: Islamabad Capital Territory; AJK: Azad Jammu & Kashmir; KPK: Khyber Pakhtunkhwa; LC: Lower Class; LMC: Lower Middle Class; MC: Middle Class; UMC: Upper Middle Class).

21.	Libanicoccus	[Province of Birth:AJK/Punjab; REF(ICT)], [Province of Residence:KPK/Sindh REF(ICT)], [Source of Drinking Water: Bottled/Tap; REF(Filtered)] [Socioeconomic Status:UMC; REF(LC)], Buttermilk Consumption:Yes, Desighee Consumption:Yes, Eggs Consumption:Yes, Tea Consumption:Yes, Vegetables Consumption:Yes, [Deep Fried Food Consumption: Everyday; REF(Rarely)]	[Province of Residence:AJK; REF(ICT)], [Education:Intermediate; REF(Primary)] Fish Consumption:Yes, Butter Consumption:Yes, Breakfast 5 days/week:Yes, Lunch 5 days/week:Yes, [Sugary Foods Consumption: Everyday; REF(Rarely)]	Novel genus. Some species are isolated from human stool samples. Previously no association found with the covariates	(*48*)
22.	Megamonas	[Province of Birth:Sindh; REF(ICT)], [Source of Drinking Water: Mineral; REF(Filtered)] [Education:Undergraduate; REF(Primary)] [Socioeconomic Status:LMC/UMC; REF(LC)], Mutton Consumption:Yes, Desighee Consumption:Yes, Rice Consumption:Yes, Lunch 5 days/week:Yes, [Deep Fried Food Consumption: Once/Week/Between 2 to 4 times/Week; REF(Rarely)] Junk Food Twice/ Week:Yes	[Province of Birth:AJK; REF(ICT)], [Province of Residence:Sindh; REF(ICT)], [Education:Intermediate; REF(Primary)] Chicken Consumption:Yes, Bread Consumption:Yes, Cheese Consumption:Yes, Coffee Consumption:Yes, Fresh Fruits Consumption:Yes, Naan/Roti 5 days/week:Yes, [Sugary Foods Consumption: Once/Week/Between 2 to 4 times/Week; REF(Rarely)]	Gram-negative bacteria isolated from human feces, involved in fermentation of glucose into acetate and propionate<br>Higher abundance is associated with high calorie intake<br>Western type high fat diet in Filipino adults has showed higher abundance of *Megamonas*<br>Previously higher abundance of *Megamonas* observed in chicken meat consumers<br>Overabundance of *Megamonas* has been reported in females with food addiction and obesity	(*8, 49-52*)
23.	Faecalitalea	[Province of Birth:Balochistan; REF(ICT)],	[Socioeconomic Status:MC; REF(LC)],	Belongs to phylum *Firmicutes* and are SCFA producers	(*53)*

Table S2: Literature survey significant genera (top 5 most positive and negative beta coefficients) in GLLVM associated with all sources of variability. The acronyms are as follows: (ICT: Islamabad Capital Territory; AJK: Azad Jammu & Kashmir; KPK: Khyber Pakhtunkhwa; LC: Lower Class; LMC: Lower Middle Class; MC: Middle Class; UMC: Upper Middle Class).

24.	Terrisporobacter	[Province of Birth:Balochistan/KPK; REF(ICT)], [Province of Residence:Sindh; REF(ICT)], [Socioeconomic Status:LMC/MC/UMC; REF(LC), Cream Consumption:Yes	[Source of Drinking Water: Mineral; REF(Filtered)]	Gram-positive, spore forming bacteria Found to be positively associated with oxidative stress	(*54*)
25.	[Eubacterium]_ruminantium_group	[Province of Residence:Balochistan; REF(ICT)], [Education:Masters; REF(Primary)]	[Province of Birth:Balochistan; REF(ICT)], Pickle Consumption:Yes	SCFA producer, Negative association with high fat diet	(*55)*
26.	Ruminococcaceae;CAG−352	Education:Intermediate; REF(Primary)]	[Province of Birth:Balochistan; REF(ICT)], [Source of Drinking Water: Natural_spring; REF(Filtered)]	*Ruminococcaceae* are SCFA producers *Ruminococcaceae CAG-352* are gram-positive bacteria, their decreased abundance was observed after consumption of lactic acid bacteria in obese people	(*56, 57*)
27.	Weissella	[Province of Birth:KPK; REF(ICT)], [Education:Undergraduate; REF(Primary)] Chicken Consumption:Yes, Beef Consumption:Yes, Bread Consumption:Yes, Buttermilk Consumption:Yes, Cream Consumption:Yes, Fresh Fruits Consumption:Yes, [Deep Fried Food Consumption: Everyday; REF(Rarely)]	[Province of Residence:KPK; REF(ICT)], [Deep Fried Food Consumption: Once/Week; REF(Rarely)]	Gram-positive, non-spore forming bacteria Have anti-inflammatory and probiotic potential Has been isolated from raw milk, feces, urine, sour dough, fermented cereals, meat and meat products, sugar cane, carrot juice, banana leaves and vegetables	(*58-61*)

Table S2: Literature survey significant genera (top 5 most positive and negative beta coefficients) in GLLVM associated with all sources of variability. The acronyms are as follows: (ICT: Islamabad Capital Territory; AJK: Azad Jammu & Kashmir; KPK: Khyber Pakhtunkhwa; LC: Lower Class; LMC: Lower Middle Class; MC: Middle Class; UMC: Upper Middle Class).

28.	Corynebacterium	[Education:Doctorate; REF(Primary)] Tea Consumption:Yes, Vegetables Consumption:Yes, Dry Fruits Consumption:Yes, Lunch 5 days/week:Yes, [Deep Fried Food Consumption: Once/Week; REF(Rarely)]	[Province of Birth:KPK/Punjab/Sindh; REF(ICT)], [Source of Drinking Water: Bottled; REF(Filtered)] [Socioeconomic Status:LMC/MC/UMC; REF(LC)], Cream Consumption:Yes, Rice Consumption:Yes, Fresh Fruits Consumption:Yes, Breakfast 5 days/week:Yes, Fried Food Items:Yes	Gram-positive, non-spore forming bacteria<br>*Corynebacterium* has been found as a causative agent to spoil fruits and vegetables<br>Fresh fruits consumption such as mangoes can increase the abundance of specie *Corynebacterium pyruviciproducens* which is considered as immune modulator<br>Studies have shown that *C. diphtheria*, mostly infects people from low socioeconomic status	*(62-66)*
29.	Peptoniphilus	[Province of Birth:Punjab/Sindh; REF(ICT)], Mutton Consumption:Yes, Pickle Consumption:Yes, Dry Fruits Consumption:Yes, Fresh Fruits Consumption:Yes, [Sugary Foods Consumption: Once/Week/Between 2 to 4 times/Week; REF(Rarely)]	Chicken Consumption:Yes, Fish Consumption:Yes, Yogurt Consumption:Yes, Lassi Consumption:Yes, Eggs Consumption:Yes, Honey Consumption:Yes, Rice Consumption:Yes	Gram-positive cocci, butyrate producing bacteria<br>Commensals of human gut and vagina<br>*Peptoniphilus* species can cause bloodstream, diabetic skin and soft tissue infections	(*67, 68*)
30.	Succinivibrio	[Province of Birth:Sindh; REF(ICT)] [Province of Residence:KPK; REF(ICT)], [Education:Intermediate; REF(Primary)] Chicken Consumption:Yes, Yogurt Consumption:Yes, Lassi Consumption:Yes, Vegetables Consumption:Yes, Fried Food Items:Yes	[Socioeconomic Status:LMC/MC/UMC; REF(LC)], Butter Consumption:Yes, SoftCheese Consumption:Yes, Buttermilk Consumption:Yes, Desighee Consumption:Yes, Pickle Consumption:Yes, Coffee Consumption:Yes, Lunch 5 days/week:Yes, [Deep Fried Food Consumption: Everyday; REF(Rarely)]	Gram-negative, strictly anaerobic, plant polysaccharide-fermenting commensals<br>They can downregulate the total fat intake of host<br>Higher abundance reported to be associated with high fiber diets and protein intake	(*69-71*)

Table S2: Literature survey significant genera (top 5 most positive and negative beta coefficients) in GLLVM associated with all sources of variability. The acronyms are as follows: (ICT: Islamabad Capital Territory; AJK: Azad Jammu & Kashmir; KPK: Khyber Pakhtunkhwa; LC: Lower Class; LMC: Lower Middle Class; MC: Middle Class; UMC: Upper Middle Class).

31.	Porphyromonas	[Province of Birth:Sindh; REF(ICT)], Butter Consumption:Yes, Pickle Consumption:Yes, Dry Fruits Consumption:Yes, [Sugary Foods Consumption: Between 2 to 4 times/Week; REF(Rarely)]	Fish Consumption:Yes, Eggs Consumption:Yes, Honey Consumption:Yes	Gram-negative, obligate anaerobic, non-spore forming bacteria<br>Decreased abundance of *Porphyromonas gingivalis* (Major cause of periodontitis and halitosis) observed after intake of honey	(*72-74*)
32.	[Eubacterium]_siraeum_group	[Province of Residence:Sindh; REF(ICT)], [Education:Masters; REF(Primary)] Beef Consumption:Yes, SoftCheese Consumption:Yes, Buttermilk Consumption:Yes, Honey Consumption:Yes	[Province of Birth:Sindh; REF(ICT)], [Province of Residence:AJK; REF(ICT)], Mutton Consumption:Yes, Pickle Consumption:Yes	Gram-positive, obligate anaerobes, non-spore forming, sulfur reducing and SCFA producers<br>*Eubacterium* species are found in the mammalian intestinal tract and oral cavity	(*75, 76*)
33.	Desulfovibrio	[Province of Residence:AJK/Balochistan; REF(ICT)], [Source of Drinking Water: Mineral/Natural_spring; REF(Filtered)] Lassi Consumption:Yes, Vegetables Consumption:Yes, Naan/Roti 5 days/week:Yes, [Sugary Foods Consumption: Everyday; REF(Rarely)]	[Education:Doctorate; REF(Primary)] Milk Consumption:Yes, Desighee Consumption:Yes, Dry Fruits Consumption:Yes, [Deep Fried Food Consumption: Everyday; REF(Rarely)]	Gram-negative, obligate anaerobic bacteria<br>Higher abundance has been associated with low carbohydrate high fat diet	(*40, 77*)

Table S2: Literature survey significant genera (top 5 most positive and negative beta coefficients) in GLLVM associated with all sources of variability. The acronyms are as follows: (ICT: Islamabad Capital Territory; AJK: Azad Jammu & Kashmir; KPK: Khyber Pakhtunkhwa; LC: Lower Class; LMC: Lower Middle Class; MC: Middle Class; UMC: Upper Middle Class).

34.	[Eubacterium]_xylanophilum_group	[Province of Residence:AJK/Punjab; REF(ICT)], [Source of Drinking Water: Natural_spring/Tap; REF(Filtered)] [Socioeconomic Status:LMC/MC/UMC; REF(LC)], Butter Consumption:Yes, Buttermilk Consumption:Yes, Cream Consumption:Yes, Honey Consumption:Yes, Breakfast 5 days/week:Yes	[Province of Residence:Balochistan; REF(ICT)], [Education:Intermediate; REF(Primary)] Cheese Consumption:Yes, Desighee Consumption:Yes, Vegetables Consumption:Yes, Naan/Roti 5 days/week:Yes, [Sugary Foods Consumption: Everyday; REF(Rarely)]	SCFA producer, positively associated with lipid and glucose metabolism	(*55*)
35.	Alistipes		[Province of Residence:AJK/KPK; REF(ICT)],	Gram-negative, anaerobic bacteria, non-spore forming bacteria<br>Found in healthy human gut and have protective and pathogenic role in human body	(*78*)
36.	Mitsuokella	Cream Consumption:Yes	[Province of Residence:AJK/Sindh; REF(ICT)], Cheese Consumption:Yes, Pickle Consumption:Yes, [Deep Fried Food Consumption: Everyday; REF(Rarely)]	Gram-negative bacteria, SCFA (acetate and propionate) producer	(*49, 79*)
37.	Alloprevotella		[Province of Residence:AJK; REF(ICT)],	Higher abundance has been associated with halitosis<br>*Alloprevotella rava* isolated from the human oral cavity is also associated with oral infections such as periodontitis	(*80, 81*)
38.	Marvinbryantia	[Province of Residence:Balochistan; REF(ICT)], [Source of Drinking Water: Natural_spring; REF(Filtered)]	[Education:Doctorate; REF(Primary)]	SCFA producer and cellulose-degrading bacterial genus<br>Previously associated with intestinal inflammation and bowel dysfunction	(*82, 83*)

Table S2: Literature survey significant genera (top 5 most positive and negative beta coefficients) in GLLVM associated with all sources of variability. The acronyms are as follows: (ICT: Islamabad Capital Territory; AJK: Azad Jammu & Kashmir; KPK: Khyber Pakhtunkhwa; LC: Lower Class; LMC: Lower Middle Class; MC: Middle Class; UMC: Upper Middle Class).

39.	Megasphaera	[[Education:Doctorate; REF(Primary)] [Deep Fried Food Consumption: Everyday; REF(Rarely)]	[Province of Residence:KPK; REF(ICT)], [Source of Drinking Water: Natural_spring; REF(Filtered)] [Socioeconomic Status:UMC; REF(LC)],	Gram-negative, anaerobic cocci, SCFA producer and opportunistic bacteria Higher abundance has been associated with obesity	(*57, 84*)
40.	Lachnospiraceae_FCS020_group		[Province of Residence:KPK; REF(ICT)],	SCFA producer, higher abundance reported in mice with low dose intake of red meat	(*85*)
41.	Enterococcus	[Province of Residence:Punjab; REF(ICT)], Mutton Consumption:Yes, Fish Consumption:Yes, Milk Consumption:Yes, [Sugary Foods Consumption: Between 2 to 4 times/Week; REF(Rarely)]	[Source of Drinking Water: Tap; REF(Filtered)] Dry Fruits Consumption:Yes, [Deep Fried Food Consumption: Once/Week/Between 2 to 4 times/Week; REF(Rarely)] Junk Food Twice/ Week:Yes	Gram-positive commensals in human gut, can cause infections such as urinary tract infection, endocarditis and bacteremia Found as most common lactic acid bacteria in raw and pasteurized milk, also colonize raw meat and fermented food	(*86-88*)
42.	Paraprevotella	[Province of Residence:Punjab; REF(ICT)], [Source of Drinking Water: Mineral; REF(Filtered)] [Education:Undergraduate; REF(Primary)] Beef Consumption:Yes, Cheese Consumption:Yes, Oatmeal Consumption:Yes, Rice Consumption:Yes	[Source of Drinking Water: Tap; REF(Filtered)] [Education:Intermediate/Doctorate; REF(Primary)] Vegetables Consumption:Yes, Dry Fruits Consumption:Yes, Fried Food Items:Yes	Gram-negative, anaerobic, succinic and acetic acid producer Higher abundance found to be associated with oat consumption Previously higher abundance was associated with T2D risk after low fat diet consumption	(*89-91)*
43.	RF39; RF39; RF39	[Education:Intermediate/Undergraduate/Masters; REF(Primary)] Milk Consumption:Yes, Tea Consumption:Yes	[Source of Drinking Water: Bottled; REF(Filtered)] Vegetables Consumption:Yes, [Deep Fried Food Consumption: Between 2 to 4 times/ Week; REF(Rarely)] [Sugary Foods Consumption: Everyday; REF(Rarely)]	Acetate and hydrogen producer Higher abundance associated with healthy diet intake Increase in abundance also found to be associated with tea intake	(*13, 43, 92*)
44.	Butyrivibrio	[Education:Undergraduate/Masters/Doctorate; REF(Primary)] Buttermilk Consumption:Yes	[Source of Drinking Water: Mineral; REF(Filtered)] SoftCheese Consumption:Yes	Gram-negative, strictly anaerobic, non-spore forming, butyrate producer Higher abundance associated with tap drinking water in Pakistan	(*8, 93*)

Table S2: Literature survey significant genera (top 5 most positive and negative beta coefficients) in GLLVM associated with all sources of variability. The acronyms are as follows: (ICT: Islamabad Capital Territory; AJK: Azad Jammu & Kashmir; KPK: Khyber Pakhtunkhwa; LC: Lower Class; LMC: Lower Middle Class; MC: Middle Class; UMC: Upper Middle Class).

45.	Escherichia-Shigella		[Source of Drinking Water: Natural_spring; REF(Filtered)]	Gram-negative bacteria. *Escherichia* is part of normal gut flora but both bacteria can be pathogenic<br>Involved in decreasing the abundance of SCFA producing bacteria	(*94*)
46.	Lachnospiraceae_group-001	[Education:Doctorate; REF(Primary)]	Beef Consumption:Yes	Member of SCFA producing family *Lachnospiraceae*<br>Previously reported to play beneficial role in alleviating T2D	(*95*)
47.	Catenibacterium		[Education:Doctorate, REF(Primary)]	Gram-positive, obligatory anaerobes<br>Showed positive association with dietary animal fat intake	(*96*)
48.	Muribaculaceae	Cream Consumption:Yes		Mostly found in murine gut microbiome, found in humans in lower abundance<br>Functional potential yet to be described	(*97*)
49.	Rikenellaceae;dgA−11_gut_group		Lunch 5 days/week:Yes	Acetate producer, in mouse models found to improve lipid metabolism	(*98*)
50.	[Eubacterium]_eligens_group	Naan/Roti 5 days/week:Yes		Previously reported to be positively associated with healthy diet	(*99*)
51.	Sutterella		[Deep Fried Food Consumption: Everyday; REF(Rarely)]	Gram-negative, non-spore forming commensals<br>Their role in human body is still unkown	(*100*)

Supplementary Questionnaire

Pakistan Microbiome Initiative

A. OFFICIAL USE ONLY

Inspection Date and Time:

Gut Kit ID:

Note: You may decline to answer any question.

B. BIOGRAPHICAL DATA

Name:___________________________ *E*-mail: ___________________________

Phone no:________________________ Gender: ___________________________

Age: ___________________________ Marital Status: _________________________

No. of family members living in the household: ___________________________

City of Birth:_____________________ Current City: __________________________

Ethnicity:________________________ Caste: __________________________

C. VITAL SIGNS

Height (*in*): Weight (*kg*): BMI (kg/m^2):

Blood Glucose (*mg/dl*): Pulse Rate (*per min*):

Temperature (*F°*): Blood Pressure (*mmHg*):

D. <u>EXCLUSION CHECKLIST (If you answer 'Yes' to any question from 1-17, please do not proceed with the questionnaire).</u>

#	**Question**	**Yes**	**No**
1	Are you aged below 18?		
2	Is your BMI <18 or >30 Kg/m^2?		
3	Have you taken any antibiotics in the last 3 months?		
4	Have you taken any multivitamins or dietary supplements in the last 1 month?		

5	Do you have (or had) acute or chronic diarrhea in the last 2 months?		
6	Do you have history of hypertension, colon cancer, or inflammatory bowel disease?		
7	Are you lactose or gluten intolerant?		
8	Are you allergic to any food?		
9	Do you have irregular menstrual cycles (that is less than 21 or more than 35 days apart)?		
10	Are you pregnant or lactating?		
11	Have you been vaccinated for any disease in the last 3 months?		
12	Do you currently have urinary tract infection?		
13	Do you consume any recreational drugs like smoking, marijuana?		
14	Have you travelled internationally in the last 6 months?		
15	Have you had bloody stools, constipation, dizziness, or fever in the last 14 days?		
16	Did you contract COVID-19 in the last 3-6 months?		
17	Have you been diagnosed with any chronic (that lasts one year or more) medical or dental condition(s)?		

E. DIETARY HABITS

#	Question	Yes	No	N/A	No answer
**1**	Do you eat breakfast on at least 5 days each week?				
**2**	Do you eat lunch on at least 5 days each week?				
**3**	Do you eat dinner on at least 5 days each week?				
**4**	Do you consume snacks in between meals?				
**5**	Do you consume honey regularly?				
**6**	Do you eat goat and/or lamb meat regularly?				
**7**	Do you eat chicken regularly?				
**8**	Do you eat fish regularly?				
**9**	Do you eat beef regularly?				
**10**	Do you eat rice regularly?				
**11**	Do you eat naan/roti on at least 5 days each week?				
**12**	Do you eat vegetables regularly?				
**13**	Do you consume “junk food” (e.g. fried chicken burgers, pizza) at least twice every week?				
**14**	Do you consume any of the following probiotics every day? Please tick mark.<br>• Yogurt<br>• Pickles<br>• Soft cheese<br>• Buttermilk				
**15**	Do you consume any of the following prebiotics every day? Please tick mark.<br>• Maple syrup<br>• Dark chocolate<br>• Asparagus				

	• Oatmeal<br>• Legumes				
**16**	Do you consume eggs every day?				
**17**	Do you consume following dairy products every day? please tick mark.<br>• Bread<br>• Milk<br>• Butter<br>• Cream<br>• Lassi<br>• Cheese<br>• Desi ghee				
**18**	Do you eat fresh fruits every day?				
**19**	Do you consume dry fruits 2 or more times a week?				
**20**	Do you smoke regularly?				
**21**	Do a family member or friend you are in constant contact with (>2 hours a day) smoke in your presence?				
**22**	Do you consume alcohol regularly?				
**23**	Do you consume tea every day?				
**24**	How many cups of tea do you consume per day?<br>➢ 0<br>➢ 1<br>➢ 2<br>➢ 3<br>➢ More than 3				
**25**	Do you consume coffee every day?				
**26**	How many cups of coffee do you consume per day?<br>➢ 0<br>➢ 1<br>➢ 2<br>➢ 3<br>➢ More than 3				

27. How often do you eat dinner outside instead of eating/cooking at home?

(a) Once a week (b) Between 2-4 times a week
(c) Everyday (d) Rarely

28. What best describe your diet?

(a) Vegetarian only and no meat
(b) No beef and no goat but consume chicken/fish
(c) All kinds of meat and vegetables
(d) Eat vegetables and meat but no dairy products

29. How often do you eat Samosa, pakora and other deep-fried food items in a week?

(a) Once a week (b) Between 2-4 times a week
(c) Everyday (d) Rarely

30. How often do you eat sugary foods (e.g. halwa, desserts, kheer, ice-cream, etc.)

in a week?

(a) Once a week (b) Between 2-4 times a week

(c) Everyday (d) Rarely

31. **How often do you take juices or other sugary drinks (e.g. lemonade, Rooh Afza, soft drinks, etc.) in a week?**

(a) Once a week (b) Between 2-4 times a week

(c) Everyday (d) Rarely

F. MEDICAL HEALTH QUESTIONAIRE

#	Question	Yes	No	N/A	No answer
1	Did you gain or lost weight recently?				
2	Did you get your appendix removed?				
3	Do you experience acid reflux?				
4	Do you experience gut flare-ups (Sudden pain, or outburst in gut)?				
5	Have you had your colon cleansed in the past week?				
6	Have you ever used any medication or home remedies to prevent or treat constipation? Please mention if known.				
7	Do you experience lethargy or general malaise?				
8	Did you suffer from parasitic infection (tapeworm etc.) during childhood?				
9	If so, had you undergone any treatment for parasitic infection?				
10	Did you suffer from anemia in the past?				
11	Did you have any craving for soil/chalk/sand/ or other non-food items in the past?				
12	Are you allergic to any of the following food items? If yes, please tick mark<br>• Soybeans.<br>• Peanuts.<br>• Milk.<br>• Wheat.<br>• Eggs.<br>• Fish (bass, flounder and cod)<br>• Shellfish (crab, crayfish, lobster and shrimp)<br>• Tree nuts (almonds, walnuts and pecans)				
13	Do you have any of the following problems?<br>➢ Celiac disease<br>➢ Lactose sensitivity				

**14**	Were you given antibiotics as a child?				
**15**	Were you breastfed as an infant?				
**16**	What was your mode of birth (C-section or natural), if known.				
**17**	Do you have trouble biting or chewing certain foods (apples, meat)?				
**18**	Do you have trouble swallowing?				
**19**	Have you had your tonsils removed?				
**20**	Do you regularly experience throat issues (e.g. sore throat, flu?				
**21**	Is your mouth dry?				
**22**	Do you have bad breath?				

G. GENERAL QUESTIONS

#	Question	Yes	No	N/A	No answer
**1**	Do you exercise regularly?				
**2**	Do you have children?				
**3**	Do you have pets?				
**4**	Do you have contact with livestock?				
**5**	Do you go to the dentist at least once a year?				
**6**	Do you share your toothbrush with anyone?				
**7**	Do you share your bathroom/toilet?				
**8**	Do you feel you are becoming healthier?				
**9**	Do you feel you are becoming unhealthier?				

10. How often do you poop?

(a) Once in 2-3 days (b) Once every day (c) 2-3 times a day

11. How do you feel during bowel movement?

(a) Good or very good (b) Moderate (c) Poor or painful

12. Have you felt abdominal pain in the last 2 days?

(a) Yes-extreme (b) Yes-moderate
(c) Yes-Slight (d) No-not recently

13. What is your main source of drinking water?

(a) Tap (b) Filtered
(c) Bottled (d) Mineral

14. During sampling, did you have any of the following symptoms?

(a) Bloody stools (b) Constipation (c) Dizziness
(d) Fever (e) Diarrhea (f) Insomnia

(g) Migraine (h) Stomach pain (i) Rash
(j) Headache

15. How often do you get sick?
(a) Never (b) Rarely (c) Sometimes
(d) Frequently (e) All the time

16. What best describes your employment status?
(a) Full-time (b) Self-employed (c) Part-time
(d) Unemployed (e) Student (f) Retired

17. How many hours per week do you work?
(a) Less than 20 (b) 20-40
(c) 40-60 (d) more than 60

18. What best describes your relationship status?
(a) Single (b) Married (c) In a relationship/Engaged
(d) Separated/Divorced (e) Widowed

19. How do you identify yourself as a person?
(a) Very social and friendly (b) Sometimes social and sometimes friendly
(c) Shy and introvert (d) Social only with very close friends
(e) Stressed at the prospect of social gatherings

20. What is your occupation (or occupation of parent/legal guardian)?
(a) Academia (b) Labor force
(c) Military (d) Personal business
(e) Agriculture (f) Unemployed

21. What is your monthly income (or income of parent/guardian)?
(a) <20,000 PKR (b) Between 20,000 and 60,000 PKR
(c) Between 60,000 and 120,000 PKR (d) >120,000 PKR

22. What best describes your socioeconomic status?
(a) Lower class (b) Lower middle class (c) Middle class
(d) Upper middle class (e) Upper Class

23. What is the highest level of education you completed?
(a) Did not go to school (b) Matriculation (c) Intermediate
(d) Bachelor degree (e) Masters (f) Doctorate

24. In general, how would you describe your health?
(a) Excellent (b) Good (c) Moderately healthy
(d) Poor (e) Terrible

25. Do you take naps?
(a) Yes-usually (b) Yes-sometimes (c) No-never

26. How much sleep do you get every night, on average?
(a) Less than 4 hours (b) 4-6 hours
(c) 6-8 hours (d) More than 8 hours

27. Do you have trouble falling asleep?
(a) Yes-always (b) Yes-sometimes
(c) Yes-Usually (d) No-never

28. Do you have trouble getting back to sleep once you're awake?
(a) Yes-always (b) Yes-sometimes
(c) Yes-usually (d) No-never

29. How much anxious have you felt in the past 1 week?
(a) Extremely (b) Moderately
(c) Slightly (d) Not at all

30. How much happy have you felt in the past 1 week?
(a) Extremely (b) Moderately
(c) Slightly (d) Not at all

31. How much stressed have you felt in the past 1 week?
(a) Extremely (b) Moderately
(c) Slightly (d) Not at all

32. How much healthy have you felt in the past 1 week?
(a) Extremely (b) Moderately
(c) Slightly (d) Not at all

33. How much tired have you felt in the past 1 week?
(a) Extremely (b) Moderately
(c) Slightly (d) Not at all

34. Have you had any headaches in the past 1 week?
(a) Yes-regularly (b) Yes-sometimes (c) No

35. How often do you brush your teeth?
(a) Once a day (b) Twice a day (c) More than twice a day
(d) Occasionally (e) Never

36. What do you use for cleaning your teeth/Mouth ?
(a) Miswak (b) Powder (c) Toothpaste
(d) Gargling (e) Mouthwash (f) None of the above

37. Have you been diagnosed with any of these sexually transmitted diseases?
(a) Chlamydia (b) Gonorrhea (c) Genital herpes
(d) Genital warts (e) HIV (f) Syphilis
(g) None of the above

38. How many times have you visited your doctor in the past year?

(a) 0-3 (b) 3-6 (c) >6

39. How many major surgeries (requiring anesthesia) have you had in your life?

(a) 0-3 (b) 3-6

(c) 6-9 (d) >10

40. Have you been vaccinated with following vaccines? If yes, then please mention the vaccination date in the text box if known.

(a) BCG (b) MMR (c) Polio

(d) Hepatitis B (e) COVID-19 (f) Rabies

(g) Other (h) None of the above

References

1. de la Cuesta-Zuluaga J, Corrales-Agudelo V, Velásquez-Mejía EP, Carmona JA, Abad JM, Escobar JS. Gut microbiota is associated with obesity and cardiometabolic disease in a population in the midst of Westernization. Scientific reports. 2018;8(1):11356.
2. Abdugheni R, Wang WZ, Wang YJ, Du MX, Liu FL, Zhou N, et al. Metabolite profiling of human-originated Lachnospiraceae at the strain level. iMeta. 2022:e58.
3. Bolte LA, Vila AV, Imhann F, Collij V, Gacesa R, Peters V, et al. Long-term dietary patterns are associated with pro-inflammatory and anti-inflammatory features of the gut microbiome. Gut. 2021;70(7):1287-98.
4. Wu F, Guo X, Zhang J, Zhang M, Ou Z, Peng Y. Phascolarctobacterium faecium abundant colonization in human gastrointestinal tract. Experimental and therapeutic medicine. 2017;14(4):3122-6.
5. Ariefdjohan MW, Dilk A, Brown-Esters ON, Savaiano DA. Intestinal Microbiota and Diet in Health. Nutrition in the Prevention and Treatment of Disease: Elsevier; 2017. p. 811-34.
6. Paliy O, Rajakaruna S. Development of Microbiota-Is the Process Continuing Through Adolescence? 2022.
7. Chaudhari DS, Dhotre DP, Agarwal DM, Gaike AH, Bhalerao D, Jadhav P, et al. Gut, oral and skin microbiome of Indian patrilineal families reveal perceptible association with age. Scientific Reports. 2020;10(1):5685.
8. Batool M, Keating C, Javed S, Nasir A, Muddassar M, Ijaz UZ. A Cross-Sectional Study of Potential Antimicrobial Resistance and Ecology in Gastrointestinal and Oral Microbial Communities of Young Normoweight Pakistani Individuals. Microorganisms. 2023;11(2):279.
9. Barrak I, Stájer A, Gajdács M, Urbán E. Small, but smelly: the importance of Solobacterium moorei in halitosis and other human infections. Heliyon. 2020;6(10):e05371.
10. Liu S, Wang Y, Zhao L, Sun X, Feng Q. Microbiome succession with increasing age in three oral sites. Aging (Albany NY). 2020;12(9):7874.
11. Amritha G, Meenakshi N, Selvabai RAP, Shanmugam P, Jayaraman P. A comparative profile of oropharyngeal colonization of Streptococcus pneumoniae and Hemophilus influenzae among HealthCare Workers (HCW) in a tertiary care hospital and non-healthcare individuals. Journal of preventive medicine and hygiene. 2020;61(3):E379.
12. Murugesan S, Al Ahmad SF, Singh P, Saadaoui M, Kumar M, Al Khodor S. Profiling the Salivary microbiome of the Qatari population. Journal of Translational Medicine. 2020;18(1):1-16.
13. Wang L, Shu X-O, Cai H, Yang Y, Xu W, Wu J, et al. Tea Consumption and Gut Microbiome in Older Chinese Adults. The Journal of Nutrition. 2023;153(1):293-300.
14. Rahman B, Al-Marzooq F, Saad H, Benzina D, Al Kawas S. Dysbiosis of the Subgingival Microbiome and Relation to Periodontal Disease in Association with Obesity and Overweight. Nutrients. 2023;15(4):826.
15. Balakrishnan B, Selvaraju V, Chen J, Ayine P, Yang L, Ramesh Babu J, et al. Ethnic variability associating gut and oral microbiome with obesity in children. Gut Microbes. 2021;13(1):1882926.

16. Pasquali F, Valero A, Possas A, Lucchi A, Crippa C, Gambi L, et al. Occurrence of foodborne pathogens in Italian soft artisanal cheeses displaying different intra-and inter-batch variability of physicochemical and microbiological parameters. Frontiers in Microbiology. 2022;13.
17. MACİT E. Some Pathogenic Bacteria Isolated and Identified from Traditionally Produced Turkish White Cheese. Black Sea Journal of Agriculture. 2023;6(2):190-6.
18. Charrier TCPLC. 11 The Family Coriobacteriaceae. 2014.
19. Neumann A, Björck L, Frick I-M. Finegoldia magna, an anaerobic Gram-positive bacterium of the normal human microbiota, induces inflammation by activating neutrophils. Frontiers in microbiology. 2020;11:65.
20. Brandwein M, Katz I, Katz A, Kohen R. Beyond the gut: skin microbiome compositional changes are associated with BMI. Human Microbiome Journal. 2019;13:100063.
21. Vongsa R, Hoffman D, Shepard K, Koenig D. Comparative study of vulva and abdominal skin microbiota of healthy females with high and average BMI. BMC microbiology. 2019;19(1):1-9.
22. Ali I, Liu K, Long D, Faisal S, Hilal MG, Ali I, et al. Ramadan fasting leads to shifts in human gut microbiota structured by dietary composition. Frontiers in microbiology. 2021;12:642999.
23. González S, Fernández-Navarro T, Arboleya S, de Los Reyes-Gavilán C, Salazar N, Gueimonde M. Fermented dairy foods: impact on intestinal microbiota and health-linked biomarkers. Frontiers in microbiology. 2019;10:1046.
24. Everard A, Belzer C, Geurts L, Ouwerkerk JP, Druart C, Bindels LB, et al. Cross-talk between Akkermansia muciniphila and intestinal epithelium controls diet-induced obesity. Proceedings of the national academy of sciences. 2013;110(22):9066-71.
25. Tong KPS, Green SJ, Ortiz J, Wu SC. Association between hemoglobin A1c, Vitamin C, and microbiome in diabetic foot ulcers and intact skin: A cross-sectional study. Health Science Reports. 2022;5(5):e718.
26. Zhang Y, Zuo J, Yan L, Cheng Y, Li Q, Wu S, et al. Sargassum fusiforme fucoidan alleviates high-fat diet-induced obesity and insulin resistance associated with the improvement of hepatic oxidative stress and gut microbiota profile. Journal of Agricultural and Food Chemistry. 2020;68(39):10626-38.
27. Gutiérrez-Repiso C, Moreno-Indias I, de Hollanda A, Martín-Núñez GM, Vidal J, Tinahones FJ. Gut microbiota specific signatures are related to the successful rate of bariatric surgery. American journal of translational research. 2019;11(2):942.
28. Qian L, Gao R, Hong L, Pan C, Li H, Huang J, et al. Association analysis of dietary habits with gut microbiota of a native Chinese community. Experimental and therapeutic medicine. 2018;16(2):856-66.
29. De D, Nayak T, Chowdhury S, Dhal PK. Insights of host physiological parameters and gut microbiome of indian type 2 diabetic patients visualized via metagenomics and machine learning approaches. Frontiers in Microbiology. 2022;13:914124.
30. Weiss GA, Chassard C, Hennet T. Selective proliferation of intestinal Barnesiella under fucosyllactose supplementation in mice. British Journal of Nutrition. 2014;111(9):1602-10.
31. Mancabelli L, Milani C, Lugli GA, Turroni F, Cocconi D, van Sinderen D, et al. Identification of universal gut microbial biomarkers of common human intestinal diseases by meta-analysis. FEMS microbiology ecology. 2017;93(12):fix153.

32. Xu D, Pan D, Liu H, Yang C, Yang X, Wang X, et al. Improvement in cardiometabolic risk markers following an oatmeal diet is associated with gut microbiota in mildly hypercholesterolemic individuals. Food Research International. 2022;160:111701.
33. Kaakoush NO. Insights into the Role of Erysipelotrichaceae in the Human Host. Front Cell Infect Microbiol. 2015;5:84.
34. Golloso-Gubat MJ, Ducarmon QR, Tan RCA, Zwittink RD, Kuijper EJ, Nacis JS, et al. Gut Microbiota and Dietary Intake of Normal-Weight and Overweight Filipino Children. Microorganisms. 2020;8(7).
35. Li Y, Kang Y, Du Y, Chen M, Guo L, Huang X, et al. Effects of Konjaku Flour on the Gut Microbiota of Obese Patients. Front Cell Infect Microbiol. 2022;12:771748.
36. Lerma-Aguilera AM, Pérez-Burillo S, Navajas-Porras B, León ED, Ruíz S, Pastoriza S, et al. Effects of different foods and cooking methods on the gut microbiota: an in vitro approach. 2022.
37. Senghor B, Sokhna C, Ruimy R, Lagier J-C. Gut microbiota diversity according to dietary habits and geographical provenance. Human Microbiome Journal. 2018;7:1-9.
38. BELKACEMI S, ALOU MT, MILLION M, LEVASSEUR A, KHELAIFIA S, RAOULT D. Prevalence of Treponema species in the Gut Microbiome is Linked to Bifidobacterium sp. and Bacteroides sp. 2020.
39. Craven H, McGuinness D, Buchanan S, Galbraith N, McGuinness DH, Jones B, et al. Socioeconomic position links circulatory microbiota differences with biological age. Scientific Reports. 2021;11(1):1-10.
40. Šik Novak K, Bogataj Jontez N, Petelin A, Hladnik M, Baruca Arbeiter A, Bandelj D, et al. Could Gut Microbiota Composition Be a Useful Indicator of a Long-Term Dietary Pattern? Nutrients. 2023;15(9):2196.
41. Liu X, Tong X, Jie Z, Zhu J, Tian L, Sun Q, et al. Sex differences in the oral microbiome, host traits, and their causal relationships. iScience. 2023;26(1):105839.
42. Willis JR, González-Torres P, Pittis AA, Bejarano LA, Cozzuto L, Andreu-Somavilla N, et al. Citizen science charts two major "stomatotypes" in the oral microbiome of adolescents and reveals links with habits and drinking water composition. Microbiome. 2018;6(1):1-17.
43. Wang Y, Huang J-M, Zhou Y-L, Almeida A, Finn RD, Danchin A, et al. Phylogenomics of expanding uncultured environmental Tenericutes provides insights into their pathogenicity and evolutionary relationship with Bacilli. BMC genomics. 2020;21:1-12.
44. Jiang F, Song P, Wang H, Zhang J, Liu D, Cai Z, et al. Comparative analysis of gut microbial composition and potential functions in captive forest and alpine musk deer. Applied Microbiology and Biotechnology. 2022;106(3):1325-39.
45. Huang C, Ge F, Yao X, Guo X, Bao P, Ma X, et al. Microbiome and metabolomics reveal the effects of different feeding systems on the growth and ruminal development of yaks. Frontiers in Microbiology. 2021;12:682989.
46. Batool M, Ali SB, Jaan A, Khalid K, Ali SA, Kamal K, et al. Initial sequencing and characterization of the gastrointestinal and oral microbiota in urban Pakistani adults. Frontiers in Cellular and Infection Microbiology. 2020;10:409.
47. Lo CI, Niang EHA, Sarr M, Durand G, Tall ML, Caputo A, et al. Fenollaria timonensis sp. nov., a new bacterium isolated from healthy human fresh stool. Current Microbiology. 2020;77:3780-6.

48. Bilen M, Cadoret F, Richez M, Tomei E, Daoud Z, Raoult D, et al. Libanicoccus massiliensis gen. nov., sp. nov., a new bacterium isolated from human stool. New Microbes and New Infections. 2018;21:63-71.
49. Palmas V, Pisanu S, Madau V, Casula E, Deledda A, Cusano R, et al. Gut microbiota markers associated with obesity and overweight in Italian adults. Scientific reports. 2021;11(1):1-14.
50. Watanabe M, Sianoya A, Mishima R, Therdtatha P, Rodriguez A, Ramos DC, et al. Gut microbiome status of urban and rural Filipino adults in relation to diet and metabolic disorders. FEMS microbiology letters. 2021;368(20):fnab149.
51. Shi J, Zhao D, Zhao F, Wang C, Zamaratskaia G, Li C. Chicken-eaters and pork-eaters have different gut microbiota and tryptophan metabolites. Scientific Reports. 2021;11(1):11934.
52. Dong TS, Mayer EA, Osadchiy V, Chang C, Katzka W, Lagishetty V, et al. A Distinct Brain-Gut-Microbiome Profile Exists for Females with Obesity and Food Addiction. Obesity. 2020;28(8):1477-86.
53. Ma Q, Li Y, Wang J, Li P, Duan Y, Dai H, et al. Investigation of gut microbiome changes in type 1 diabetic mellitus rats based on high-throughput sequencing. Biomedicine & Pharmacotherapy. 2020;124:109873.
54. Li H, Shang Z, Liu X, Qiao Y, Wang K, Qiao J. Clostridium butyricum alleviates enterotoxigenic Escherichia coli K88-induced oxidative damage through regulating the p62-Keap1-Nrf2 signaling pathway and remodeling the cecal microbial community. Frontiers in Immunology. 2021;12:771826.
55. Miao Z, Du W, Xiao C, Su C, Gou W, Shen L, et al. Gut microbiota signatures of long-term and short-term plant-based dietary pattern and cardiometabolic health: a prospective cohort study. BMC medicine. 2022;20(1):1-15.
56. Cui J, Yang X, Wang F, Liu S, Han S, Chen B. Effects of ammonia on growth performance, lipid metabolism and cecal microbial community of rabbits. Plos one. 2021;16(6):e0252065.
57. Burakova I, Smirnova Y, Gryaznova M, Syromyatnikov M, Chizhkov P, Popov E, et al. The effect of short-term consumption of lactic acid bacteria on the gut microbiota in obese people. Nutrients. 2022;14(16):3384.
58. Fusco V, Quero GM, Cho G-S, Kabisch J, Meske D, Neve H, et al. The genus Weissella: taxonomy, ecology and biotechnological potential. Frontiers in microbiology. 2015;6:155.
59. Teixeira CG, Fusieger A, Milião GL, Martins E, Drider D, Nero LA, et al. Weissella: An emerging bacterium with promising health benefits. Probiotics and Antimicrobial Proteins. 2021;13(4):915-25.
60. Björkroth J, Dicks LM, Endo A. The genus Weissella. Lactic acid bacteria: Biodiversity and taxonomy. 2014:417-28.
61. Kamboj K, Vasquez A, Balada-Llasat J-M. Identification and significance of Weissella species infections. Frontiers in Microbiology. 2015;6:1204.
62. Bernard KA, Funke G. Corynebacterium. Bergey's Manual of Systematics of Archaea and Bacteria. 2015:1-70.
63. Alibi S, Ferjani A, Boukadida J. Implication of Corynebacterium species in food's contamination. Journal of Coastal Life Medicine. 2016;4(5):416-9.
64. Asuncion P, Liu C, Castro R, Yon V, Rosas Jr M, Hooshmand S, et al. The effects of fresh mango consumption on gut health and microbiome–Randomized controlled trial. Food Science & Nutrition. 2023.

65. Cieplewicz P, Szymoniak-Lipska M, Jałowska M, Polańska A, Bowszyc-Dmochowska M, Tomczak H, et al. Corynebacterium diphtheriae infection as a dermatological problem–case report and review of the literature. Dermatology Review/Przegląd Dermatologiczny. 2019;106(1):25-33.
66. Zasada AA. Nontoxigenic highly pathogenic clone of Corynebacterium diphtheriae, Poland, 2004–2012. Emerging infectious diseases. 2013;19(11):1870.
67. Aujoulat F, Mazuet C, Criscuolo A, Popoff MR, Enault C, Diancourt L, et al. Peptoniphilus nemausensis sp. nov. A new Gram-positive anaerobic coccus isolated from human clinical samples, an emendated description of the genus Peptoniphilus and an evaluation of the taxonomic status of Peptoniphilus species with not validly published names. Systematic and Applied Microbiology. 2021;44(5):126235.
68. Brown K, Church D, Lynch T, Gregson D. Bloodstream infections due to Peptoniphilus spp.: report of 15 cases. Clinical Microbiology and Infection. 2014;20(11):O857-O60.
69. Bryant MP. Succinivibrio. Bergey's Manual of Systematics of Archaea and Bacteria. 2015:1-3.
70. Tan H, Nie S. Deciphering diet-gut microbiota-host interplay: Investigations of pectin. Trends in Food Science & Technology. 2020;106:171-81.
71. Tang M, Frank DN, Tshefu A, Lokangaka A, Goudar SS, Dhaded SM, et al. Different gut microbial profiles in sub-Saharan African and South Asian women of childbearing age are primarily associated with dietary intakes. Frontiers in Microbiology. 2019;10:1848.
72. Turchini GM, Francis DS, Du Z-Y, Olsen RE, Ringø E, Tocher DR. The lipids. Fish nutrition: Elsevier; 2022. p. 303-467.
73. Shiga H, Jo A, Terao K, Nakano M, Oshima T, Maeda N. Decrease of halitosis by intake of Manuka honey. General Session of IADR Barcelona. 2010;14(2):234-8.
74. Eick S, Schäfer G, Kwieciński J, Atrott J, Henle T, Pfister W. Honey–a potential agent against Porphyromonas gingivalis: an in vitro study. BMC oral health. 2014;14:1-9.
75. Wade WG. The genus Eubacterium and related genera. Prokaryotes. 2006;4:823-35.
76. Mukherjee A, Lordan C, Ross RP, Cotter PD. Gut microbes from the phylogenetically diverse genus Eubacterium and their various contributions to gut health. Gut Microbes. 2020;12(1):1802866.
77. Kuever J, Rainey FA, Widdel F. Desulfovibrio. Bergey's Manual of Systematics of Archaea and Bacteria. 2015:1-17.
78. Parker BJ, Wearsch PA, Veloo AC, Rodriguez-Palacios A. The genus Alistipes: gut bacteria with emerging implications to inflammation, cancer, and mental health. Frontiers in immunology. 2020;11:906.
79. Malan-Muller S, Valles-Colomer M, Foxx CL, Vieira-Silva S, van den Heuvel LL, Raes J, et al. Exploring the relationship between the gut microbiome and mental health outcomes in a posttraumatic stress disorder cohort relative to trauma-exposed controls. European Neuropsychopharmacology. 2022;56:24-38.
80. Ye W, Zhang Y, He M, Zhu C, Feng X-P. Relationship of tongue coating microbiome on volatile sulfur compounds in healthy and halitosis adults. Journal of Breath Research. 2019;14(1):016005.
81. Toprak NU, Duman N, Sacak B, Ozkan M, Sayın E, Mulazimoglu L, et al. Alloprevotella rava isolated from a mixed infection of an elderly patient with chronic mandibular osteomyelitis mimicking oral squamous cell carcinoma. New Microbes and New Infections. 2021;42:100880.

82. Wang Y, Qi W, Song G, Pang S, Peng Z, Li Y, et al. High-fructose diet increases inflammatory cytokines and alters gut microbiota composition in rats. Mediators of inflammation. 2020;2020.
83. Han D-S, Wu W-K, Liu P-Y, Yang Y-T, Hsu H-C, Kuo C-H, et al. Differences in the gut microbiome and reduced fecal butyrate in elders with low skeletal muscle mass. Clinical Nutrition. 2022;41(7):1491-500.
84. Marchandin H, Juvonen R, Haikara A. Megasphaera. Bergey's Manual of Systematics of Archaea and Bacteria. 2015:1-16.
85. Liu X, Tan F, Cui M, LI D, Yao P. Effects of red meat diet on gut microbiota in mice. Food Science and Technology. 2021;42.
86. García-Solache M, Rice LB. The Enterococcus: a model of adaptability to its environment. Clinical microbiology reviews. 2019;32(2):e00058-18.
87. Dapkevicius MdLE, Sgardioli B, Câmara SP, Poeta P, Malcata FX. Current trends of enterococci in dairy products: A comprehensive review of their multiple roles. Foods. 2021;10(4):821.
88. Giraffa G. Functionality of enterococci in dairy products. International journal of food microbiology. 2003;88(2-3):215-22.
89. Bressa C, Bailén-Andrino M, Pérez-Santiago J, González-Soltero R, Pérez M, Montalvo-Lominchar MG, et al. Differences in gut microbiota profile between women with active lifestyle and sedentary women. PloS one. 2017;12(2):e0171352.
90. Xu D, Feng M, Chu Y, Wang S, Shete V, Tuohy KM, et al. The prebiotic effects of oats on blood lipids, gut microbiota, and short-chain fatty acids in mildly hypercholesterolemic subjects compared with rice: a randomized, controlled trial. Frontiers in immunology. 2021;12:787797.
91. Camargo A, Vals-Delgado C, Alcala-Diaz JF, Villasanta-Gonzalez A, Gomez-Delgado F, Haro C, et al. A diet-dependent microbiota profile Associated with incident type 2 diabetes: from the CORDIOPREV study. Molecular Nutrition & Food Research. 2020;64(23):2000730.
92. Yu D, Nguyen SM, Yang Y, Xu W, Cai H, Wu J, et al. Long-term diet quality is associated with gut microbiome diversity and composition among urban Chinese adults. The American Journal of Clinical Nutrition. 2021;113(3):684-94.
93. Willems A, Collins MD. Butyrivibrio. Bergey's Manual of Systematics of Archaea and Bacteria. 2015:1-20.
94. Baltazar-Díaz TA, González-Hernández LA, Aldana-Ledesma JM, Peña-Rodríguez M, Vega-Magaña AN, Zepeda-Morales ASM, et al. Escherichia/Shigella, SCFAs, and Metabolic Pathways—The Triad That Orchestrates Intestinal Dysbiosis in Patients with Decompensated Alcoholic Cirrhosis from Western Mexico. Microorganisms. 2022;10(6):1231.
95. Gryaznova M, Dvoretskaya Y, Burakova I, Syromyatnikov M, Popov E, Kokina A, et al. Dynamics of changes in the gut microbiota of healthy mice fed with lactic acid bacteria and bifidobacteria. Microorganisms. 2022;10(5):1020.
96. Shin J-H, Sim M, Lee J-Y, Shin D-M. Lifestyle and geographic insights into the distinct gut microbiota in elderly women from two different geographic locations. Journal of physiological anthropology. 2016;35:1-9.
97. Smith BJ, Miller RA, Schmidt TM. Muribaculaceae genomes assembled from metagenomes suggest genetic drivers of differential response to acarbose treatment in mice. Msphere. 2021;6(6):e00851-21.

98. Deng L, Chen S, Meng W, Zhou Z, Liu H, Zhong Z, et al. Changes in Gut Microbiota Composition Associated with the Presence of Enteric Protist Blastocystis in Captive Forest Musk Deer (Moschus Berezovskii). Microbiology Spectrum. 2022;10(4):e02269-21.
99. Latorre-Pérez A, Hernández M, Iglesias JR, Morán J, Pascual J, Porcar M, et al. The Spanish gut microbiome reveals links between microorganisms and Mediterranean diet. Scientific Reports. 2021;11(1):1-12.
100. Hiippala K, Kainulainen V, Kalliomäki M, Arkkila P, Satokari R. Mucosal prevalence and interactions with the epithelium indicate commensalism of Sutterella spp. Frontiers in microbiology. 2016;7:1706.
